# The population context is a driver of the heterogeneous response of epithelial cells to interferons

**DOI:** 10.1038/s44320-024-00011-2

**Published:** 2024-01-25

**Authors:** Camila Metz-Zumaran, Zina M Uckeley, Patricio Doldan, Francesco Muraca, Yagmur Keser, Pascal Lukas, Benno Kuropka, Leonie Küchenhoff, Soheil Rastgou Talemi, Thomas Höfer, Christian Freund, Elisabetta Ada Cavalcanti-Adam, Frederik Graw, Megan Stanifer, Steeve Boulant

**Affiliations:** 1 Department of Molecular Genetics and Microbiology, University of Florida, College of Medicine, 1200 Newell Drive, 32610 Gainesville, FL USA; 2 Department of Infectious Disease, Virology, University Hospital Heidelberg, Im Neuenheimer Feld 344, 60120 Heidelberg, Germany; 3 BioQuant-Center for Quantitative Biology, Heidelberg University, 60120 Heidelberg, Germany; 4 Institute of Chemistry and Biochemistry, Protein Biochemistry, Freie Universität Berlin, Thielallee 63, 14195 Berlin, Germany; 5 Laboratory of Systems Pharmacology, Department of Systems Biology, Harvard Medical School, Boston, MA USA; 6 Division of Theoretical Systems Biology, German Cancer Research Center (DKFZ), Heidelberg, Germany; 7 Max Planck Institute for Medical Research, Heidelberg, Germany; 8 Cellular Biomechanics, University of Bayreuth, Bayreuth, Germany; 9 Interdisciplinary Center for Scientific Computing, Heidelberg University, Heidelberg, Germany; 10 Department of Medicine 5, Friedrich-Alexander-Universität Erlangen-Nürnberg, 91054 Erlangen, Germany

**Keywords:** Interferons, Heterogeneity, Population Context, Epithelium, Polarity, Immunology, Signal Transduction

## Abstract

Isogenic cells respond in a heterogeneous manner to interferon. Using a micropatterning approach combined with high-content imaging and spatial analyses, we characterized how the population context (position of a cell with respect to neighboring cells) of epithelial cells affects their response to interferons. We identified that cells at the edge of cellular colonies are more responsive than cells embedded within colonies. We determined that this spatial heterogeneity in interferon response resulted from the polarized basolateral interferon receptor distribution, making cells located in the center of cellular colonies less responsive to ectopic interferon stimulation. This was conserved across cell lines and primary cells originating from epithelial tissues. Importantly, cells embedded within cellular colonies were not protected from viral infection by apical interferon treatment, demonstrating that the population context-driven heterogeneous response to interferon influences the outcome of viral infection. Our data highlights that the behavior of isolated cells does not directly translate to their behavior in a population, placing the population context as one important factor influencing heterogeneity during interferon response in epithelial cells.

## Synopsis

The heterogeneous response to interferons in epithelial cells is determined by the population context (i.e. a cell’s positions within a population) through cell polarization and basolateral localization of the interferon receptor.

- Isogenic epithelial cells respond in a heterogeneous manner to type I and type III interferons.
- The population context (position of a cell within a population) drives the heterogeneous response to interferons, with cells located at the edge of a population being more responsive than cells located in the center of a population.
- Cells in the center of a population are refractive to interferon treatment from their apical side due to the polarized basolateral distribution of the interferon receptors.
- By controlling the response to interferon treatment, the population context affects susceptibility to viral infection.

## Abstract (web summary)

The heterogeneous response to interferons in epithelial cells is determined by the population context (i.e. a cell’s positions within a population) through cell polarization and basolateral localization of the interferon receptor.

## Graphical abstract

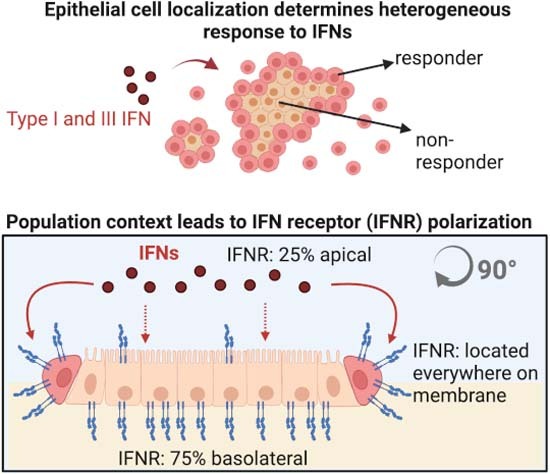

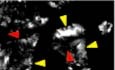

## Introduction

Interferons (IFNs) are the first line of antiviral innate immune defense. There are three types of IFNs, type I, II, and III. While type II IFNs are mostly produced by immune cells (Kawai and Akira, 2006; Koyama et al, 2008), type I and type III IFNs are produced by all cell types. Type I IFNs and type III IFNs bind to the heterodimeric receptors IFN-alpha receptor (IFNAR) IFNAR1/IFNAR2 (Novick et al, 1994) and IFN-lambda receptor (IFNLR) IFNLR1/IL10Rβ (Kotenko et al, 2003; Sheppard et al, 2003), respectively. The IFNLR1 subunit of the type III IFN receptor is mostly expressed in epithelial cells and in some immune cells conferring the type III IFNs a key role to protect mucosal surfaces against viral infection (Sommereyns et al, 2008; Pott et al, 2011; Mordstein et al, 2010). In response to virus infection, IFNs are produced and secreted from infected cells and bind to their respective receptors inducing the activation of the Janus kinase (JAK)-Signal Transducer and Activator of Transcription Proteins (STAT) signaling pathway (Levy et al, 2011). Following activation of STAT1 and STAT2 via phosphorylation, these proteins associate with Interferon Regulatory Factor 9 (IRF9) to form the Interferon Stimulated Gene Factor 3 (ISGF3) complex, which translocates into the nucleus, leading to transcription of interferon stimulated genes (ISGs) that combat viral replication and spread (Schindler et al, 2007; Stanifer et al, 2019).

Most studies aiming at understanding regulation of signal transduction during IFN-mediated signaling have classically used bulk analysis approaches, where the measured parameters represent an average of an entire cell population. For example, when monitoring the kinetics of STAT1/STAT2 activation following IFN treatment, bulk approaches will only provide the time for STAT1/STAT2 to be phosphorylated within the cell population (average phosphorylation time). This does not provide information related to the proportion of cells that responded to IFNs and activated STAT1/STAT2 and similarly, does not address whether all cells responded with the same kinetics to the IFN treatment. Recent studies have discovered cell-to-cell variability to be a central feature of cell populations, even for genetically identical cells (isogenic cells) growing in the same environment (Altschuler and Wu, 2010). This cell-to-cell variability in response to various stimuli is often referred to as single cell heterogeneity within a cell population. The effects of single cell heterogeneity are wide-ranging, and affect central cellular pathways (Spencer et al, 2009; Tay et al, 2010), phenotypic outcomes (Roesch et al, 2010; Gupta et al, 2011), and even drug sensitivity (Slack et al, 2008; Sharma et al, 2010). Intriguingly, cell-to-cell variability is a prevalent characteristic of IFN-dependent signaling. Early work from Rand et al (Rand et al, 2012) directly addressed the heterogeneous response to IFNs. They treated murine fibroblasts Swiss 3T3 with IFNβ and observed distinct cell subpopulations with some cells responding to IFNs and expressing ISGs, and other cells non-responding to IFN and as a consequence not expressing ISGs. Importantly, if the non-responder population is sorted, recultured and treated again with IFNs, the same heterogeneous response characterized by a responder and a non-responder subpopulation is observed. This finding excludes the existence of a stable fraction of unresponsive cells in the isogenic cell line. Importantly, when Rand et al (Rand et al, 2012) analyzed the induction of an antiviral state, the non-responder population was permissive to virus infection, while the responder population was protected. Similar follow-up studies supported a heterogonous response to IFNs, including to IFNβ (type I), IFNα (type I) and IFNλ3 (type III), in a variety of cell types (human airway epithelial cell line A549, hepatocyte-derived epithelial-like cell line Huh7.5, primary human hepatocytes, and murine intestinal epithelial cells) (Schmid et al, 2015b; Bhushal et al, 2017a; Maier et al, 2022; Bauhofer et al, 2012). Rand et al (Rand et al, 2012) developed a mathematical model and suggested that cell intrinsic stochasticity is responsible for a heterogeneous response to IFNβ in murine fibroblasts. Stochastic events are a probabilistic distribution of behavior rather than deterministic phenotypes regulated by the molecular machinery. Stochastic events arise from ‘noise’, a term describing some randomness of molecular interactions in the cellular environment (Andrews et al, 2009). This model of stochastic origin of cell-to-cell variability was supported with experimental data (stimulation of Huh7.5 with IFNα) and mathematical modeling simulations conducted by Maier et al (Maier et al, 2022). Altogether, stochastic events are pivotal factors contributing to cell-to-cell variability during IFN-signaling.

A major determinant for cellular variability in adherent cell culture systems is the population context (Snijder and Pelkmans, 2011). The parameters that constitute the population context of an individual cell are the local cell density, cell-to-cell contacts, and relative location within the population. Various molecular mechanisms sense these parameters and translate them to a population-dependent behavior including changes in polarization state, proliferation rate, sensitivity to apoptosis, metabolic state, and cell motility (Snijder and Pelkmans, 2011). Population-dependent behavior thereby could shape the distribution of single-cell phenotypic properties, leading to the population heterogeneity in genetically identical cells. The population context has a large impact on molecular and cell biology. Snijder et al (Snijder et al, 2009) showed that virus infectivity, endocytic events, and cellular lipid composition were determined by adaptation of cells to their population context. It was further demonstrated that cell confluence, a central parameter of the population context, induces major changes at the molecular level, leading to differential lipid distribution (Kavaliauskiene et al, 2014) or protein expression (Trajkovic et al, 2019) when cells are grown at high vs. low density. As such, IFN-dependent signaling in isogenic populations is not only dependent on stochastic events within the cell, but also affected by the population context. We here aim to address which role the population context plays on the heterogenous IFN-dependent response in a cell population in adherent intestinal epithelial cells (IECs).

The intestinal epithelium separates host tissue from microbiota and is required to act as a barrier to maintain homeostasis. IECs polarize and are organized in an impenetrable monolayer. Adjacent cells form junctional complexes as intercellular attachment structures which prevents molecule diffusion (Chelakkot et al, 2018). This results in a highly dense tissue, in which microbiota is in contact with the apical membrane of IECs and cannot trespass to the *lamina propria*, which is in contact with the basolateral side of IECs. Therefore, in vivo, the population context of IECs is characterized by high local density, polarization, and cells being embedded in a monolayer.

It was reported that treatment of a clonal population of mouse-derived IECs with type I or type III IFNs induced a heterogeneous response characterized by a responder and a non-responder sub-population independent on the cytokine concentration (Bhushal et al, 2017b). This heterogeneity in IFN response was also seen in human IECs, where even very high concentrations of type III IFNs were never able to fully protect all cells from virus infection while type I IFN was (Pervolaraki et al, 2018a). Despite the extensive study of antiviral innate immunity in the intestinal epithelium as part of the mucosal barrier, little emphasis has been put on understanding how the population context affects the response to IFNs. In our study, we aim to better understand the origins of a heterogeneous response to IFNs in human IECs. We combined spatial and quantitative analysis of IFN-mediated signaling with micropatterning approaches to address how the population context impacts response of IECs to IFN treatment. Micropatterning of defined adhesion areas for cell populations allow to tune geometry and size while keeping control over cell density (Zambarda et al, 2022). We observed that only a fraction of the cells seeded in partially confluent monolayer responded to apical IFN treatment, and that responsive cells were positioned at the edge of the cell population. Accordingly, cells seeded in a confluent monolayer were less responsive to IFNs as compared to sparsely seeded cells, in which most of the cells had no neighbors and had a similar population context to cells located at edges of a population. This spatial regulation of IFN response disappeared when cells were treated from the basolateral side, which we identified was due to a polarization of the IFN receptor. Furthermore, using IECs which would not form tight barriers, we could demonstrate that the heterogeneity in IFN response during apical treatment is caused by restricted accessibility of the IFN to the respective receptor. Together our results highlight that the population context is one factor impacting IFN signaling in epithelial cells, and is a key parameter to consider when performing experiments in polarized cells.

## Results

### Cell location within a population influences its response to IFN

**Figure 1:**
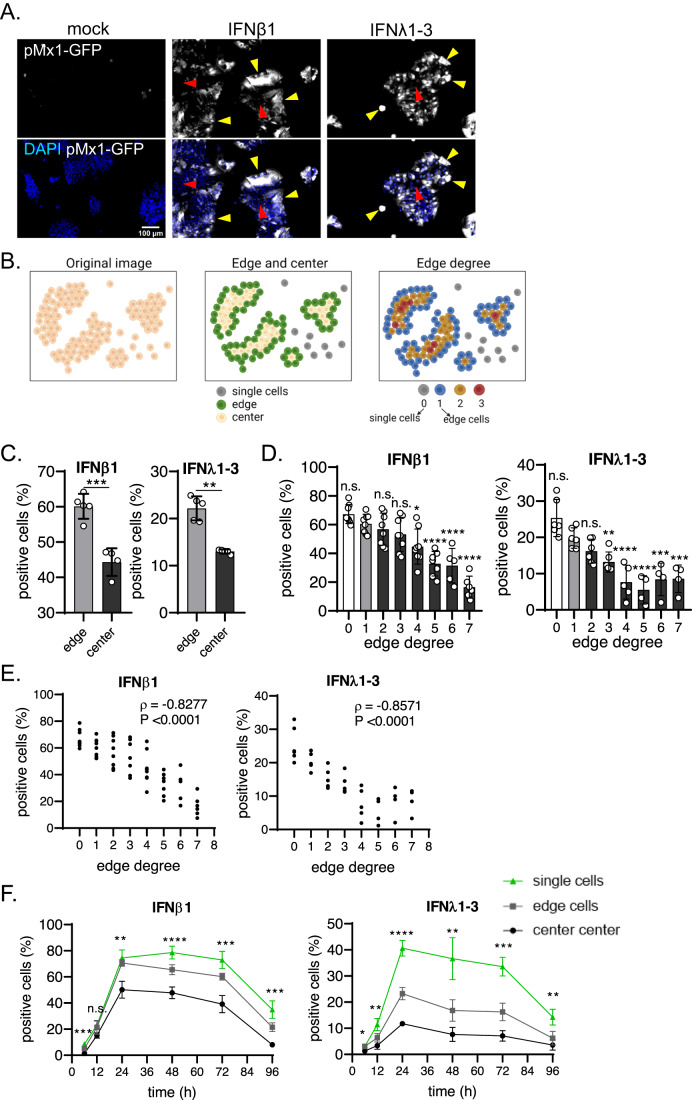
Location of a cell within a population determines its responsiveness to IFN treatment. (**A**–**E**) T84-prom-Mx1-fp seeded at medium density were mock treated or treated with 2000 IU/mL IFNβ1 or 300 ng/mL IFNλ1-3 for 24 h. Cell nuclei were stained with DAPI and fluorescence microscopy was performed. (**A**) Representative images of cells (nuclei stained with DAPI are blue) expressing the fluorescent reporter (white). Yellow arrows point at IFN responder single cells or cells located at the colony edges. Red arrows point at non-responder cells in the colony center. (**B**) Correlation between single cell location and IFN-responsiveness was assessed using DBSCAN-CellX. Schematics depicting how the tool annotates cells according to their location at the edge or at the center of a cluster, or according to their edge degree are shown. (**C**, **D**) Quantification of the percentage of positive fluorescent cells as compared to mock-treated cells. (**C**) Edge vs. center cells. (**D**) Percentage of positive fluorescent cells dependent on the edge degree. An edge degree of 0 define single cells (no neighbors) and edge degree 1 are cells at the border of a colony. The higher the edge degree, the larger the distance from the edge. Each dot represents the percentage of positive cells dependent on the edge degree for a single well averaged over 12 individual fields of view per well. (**E**) Regression analysis and coefficient of correlation (ρ) calculated for (**D**) using a two-tailed nonparametric Spearman correlation. (**F**) T84-prom-Mx1-fp seeded at medium density were mock treated or treated with 2000 IU/mL IFNβ1 or 300 ng/mL IFNλ1-3 for 6, 12, 24, 48, 72, and 96 h. Quantification of the percentage of positive fluorescent cells as compared to mock-treated cells for single, edge and center cells. (**C**, **D**, **F**) Error bars indicate standard deviations. *n* ≥ 3 biological replicates. n.s. = not significant. *P* < 0.05 *, *P* < 0.01 **, *P* < 0.001 ***, *P* < 0.0001 **** as determined by (**C**) Unpaired *t* test with Welch’s correction, (**D**) ordinary one-way ANOVA with Dunnett’s multiple comparison test using edge degree 1 as reference and (**F**) ordinary one-way ANOVA within each time-point. Source data are available online for this figure.

Previous work has shown that within a cell population, a fraction of the cells do not respond to IFNs despite being genetically identical to the responding cells and having fully functional signal transduction pathways downstream of the receptors (Patil et al, 2015; Rand et al, 2012; Zhao et al, 2012; Wimmers et al, 2018; Schmid et al, 2015a). To address whether the population context (i.e., location of a cell within a population) can modulate IFN-mediated signaling, we exploited our previously described human-colon carcinoma T84 cells expressing a fluorescent protein (fp) under the transcriptional control of the interferon-stimulated gene (ISG) MX1 promoter (T84-prom-Mx1-fp) (Doldan et al, 2022a). With this reporter system, cells are only fluorescent upon IFN-mediated signaling (Fig. EV1A,B), thereby allowing the visualization of the response of each individual cell within a population. T84-prom-Mx1-fp cells were mock-treated or treated with saturating concentration of type I and type III IFNs. These concentrations (2000 IU/mL IFNβ1 (type I IFN) or 300 ng/mL IFNλ1-3 (type III IFN)) were determined from our previous studies as concentrations inducing the maximum ISGs and conferring the maximum antiviral property to T84 cells (Pervolaraki et al, 2018b; Metz-Zumaran et al, 2022a; Doldan et al, 2022b). This ensures the heterogeneity of the cellular response to IFN can be investigated without concerns that differences in responsiveness between cells might be caused by insufficient cytokine levels. 24 h after treatment, T84-prom-Mx1-fp cells were fixed and analyzed using fluorescence microscopy. When seeded at a “medium” density, IECs often form small cellular colonies, instead of attaching to the substrate as individual cells uniformly distant from each other (Fig. 1A). This property is likely due to the intrinsic function of IECs to form a cellular epithelial monolayer through tight junction formation. Interestingly, analysis of the location of the cells that became fluorescent upon IFN treatment revealed that mostly isolated cells (cells lacking neighboring cells) and cells located at the edge of a small cellular colony respond to IFNs (Fig. 1A, yellow arrows). In contrast, cells in the center of a colony remained unresponsive (Fig. 1A, red arrows). This suggests that the position of a cell in a population may influence its responsiveness to IFNs.

**Figure EV1:**

IFN-sensing reporter cell line and intestinal organoids seeded in 2D show heterogeneity during IFN treatment. (**A**) Schematic depicting the T84 prom-Mx1-fp reporter cell line. Upon interaction of IFNs with their receptor, downstream signaling induces nuclear translocation of the transcription complex ISGF3. This leads to expression of the fluorescent protein under control of the ISG Mx1 promoter. The fluorescent protein accumulates in the cytosol and can be visualized by fluorescence microscopy. (**B**) Representative images showing expression of the fluorescent reporter (white) after mock, 2000 IU/mL IFNβ1, or 300 ng/mL IFNλ1-3 treatment. Nuclei are stained with DAPI (blue). *n* = 3 biological replicates. Scale bar = 100 µm. (**C**) T84-prom-Mx1-fp seeded at medium density were mock treated or treated with 2000 IU/mL IFNβ1 or 300 ng/mL IFNλ1-3 for 24 h. The positive fluorescent cells were determined for each edge degree and the mean fluorescence intensity (MFI) was measured within each positive cell. The MFI was normalized to the mock-treatment MFI of the corresponding edge degree (normalized fluorescence). (**D**) T84-prom-Mx1-fp seeded at medium density were mock treated or treated with 2000 IU/mL IFNβ1 or 300 ng/mL IFNλ1-3 for 6, 12, 24, 48, 72, and 96 h. Quantification of the percentage of total positive fluorescent cells as compared to mock-treated cells. (**E**, **F**) Ileum-derived organoids were seeded in 2-dimensions (2D) and treated apically with 2000 IU/mL IFNβ1 or 300 ng/mL IFNλ1-3. 24 h post-treatment, samples were fixed and indirect immunofluorescence was performed against ISG15 (green). Nuclei were stained with DAPI (blue). (**E**) Representative images are shown. Yellow arrows point at IFN responder cells located at the colony edges. Red arrows point at non-responder cells in the colony center. Scale bar = 100 µm. (**F**) Quantification of the ISG mean fluorescence intensity (arbitrary units (a.u.)) at the edge or the center of cell clusters. (**D**, **F**) *n* ≥ 3 biological replicates, error bars indicate the standard deviation. n.s. = not significant. *P* < 0.05 *, *P* < 0.01 **, *P* < 0.001 ***, *P* < 0.0001 **** as determined by (**D**) ordinary one-way ANOVA with Dunnett’s multiple comparison test using edge degree 1 as reference and (**F**) Unpaired *t* test with Welch’s correction.

To quantify how the location of a cell within a population impacts its response to IFN, we performed an unbiased analysis of the cell positioning using DBSCAN-CellX (URL: https://github.com/GrawLab/DBSCAN-CellX/) (Küchenhoff et al, 2023). DBSCAN-CellX is a density-based clustering algorithm allowing us to determine the spatial distribution and positioning of cells in a 2-dimension (2D) plane. In brief, our analytic pipeline allows for the registration of the XY-coordinates of each individual cell and for the unbiased determination of whether cells are located at the edge or the center of a colony (Fig. 1B, center panel). In addition, the relative location of an individual cell with regard to the edge or center of a cell population is quantified by the “edge degree”, which represents the distance of a cell from the edge of its colony. The higher the edge degree, the larger the distance from the edge. Cells at the edge are defined by an edge degree of 1, while an edge degree of 0 represents single cells that have no neighbors and cells with an edge degree of 2 or higher are fully embedded in the cell population (Fig. 1B, right panel).

Analysis of the location of the IFN responsive cells within the cell population using the DBSCAN-CellX algorithm revealed that, independent of whether cells were treated with type I or type III IFNs, a significantly higher percentage of edge cells responded to IFNs as compared to center cells (Fig. 1C). Analysis of IFN-dependent signaling in correlation to the edge degree showed that the most responsive cells are those lacking neighboring cells (edge degree 0) (Fig. 1D). Importantly, our data show that cells that are more embedded in a cell population (higher edge degree) respond less to both IFNs compared to cells located closer to the edge of the population (lower edge degree) (Fig. 1D). We observed a significant (*p* < 0.0001) negative correlation between the edge degree and the percentage of cells responding to both IFNs (Spearman’s correlation coefficient *ρ* = 0.8266 for IFNβ1 and *ρ* = 0.8571 for IFNλ1-3 treatment) (Fig. 1E), further reinforcing that cells located inside a population are less responsive to both type I and type III IFNs. To test whether cell localization within a population also affects the magnitude of the response of cells to IFNs, we calculated the normalized fluorescence intensities of our reporter cell line for each edge degree. Upon IFNβ1 treatment, the magnitude of IFN signaling significantly decreased in the responder cells embedded within the population (higher edge degree) (Fig. EV1C). On the contrary, treatment with IFNλ1-3 did not elicit a differential IFN signaling magnitude among responsive cells within the population (same intensity for all edge degrees) (Fig. EV1C). The absence of a differential magnitude of IFN signaling upon IFNλ1-3 treatment between cells located at the edge or embedded within the population is likely due to the fact that cellular response to IFNλ1-3 follows more an on/off mechanism than a dose-dependent response (Pervolaraki et al, 2018a). Together, our data suggest that the cellular location within a population influences whether a cell will respond to type I and III IFNs or not.

Finally, to rule out the possibility that the reduced response of center cells to IFNs is the result of different response kinetic to IFN treatments (earlier or delayed response), we assessed the number of IFN responsive cells overtime for isolated, edge, and center cells. We observed that the percentage of responsive cells (expressing prom-Mx1-fp) peaked at 24 h post-treatment for both type I and III IFN (Fig. EV1D). Importantly, analyses revealed that at all time points, single cells were the most responsive to both IFNs, while center cells where the least responsive (Fig. 1F). To address whether the population context also impacts IFN signaling in primary epithelial cells, we used human ileum-derived organoids. Organoids seeded in 2D were treated apically with IFNβ1 and IFNλ1-3. Activation of IFN signaling was monitored by immunostaining of ISG15. Similar to T84 cells, in ileum-derived organoids, cells at the edge were responding significantly stronger to IFNs (Fig. EV1E,F, yellow arrows) than cells localized in the center of a cell cluster (Fig. EV1E,F, red arrows). Altogether, by correlating IFN responsiveness of individual cells to their locations within a population, our data show that IECs located at the edge of a cellular population are overall more responsive than IECs embedded in the population.

### Micropatterning results in standardized IEC populations, revealing spatial segregation of immune signaling

**Figure 2:**
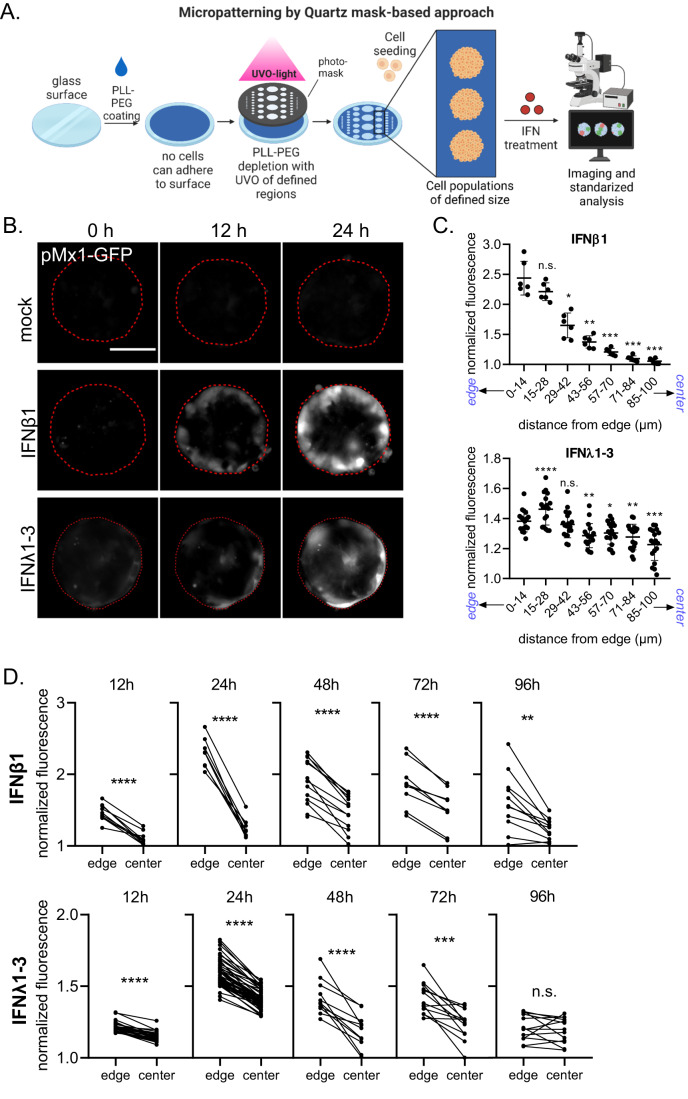
Cells located in the center of a cellular colony are non-responsive to IFNs. (**A**) Schematic depicting the glass micropatterning approach using a Quartz-mask. (**B**–**D**) T84-prom-Mx1-fp cells seeded on circular micropatterns (200 µm diameter) were mock treated or treated with 2000 IU/mL IFNβ1 or 300 ng/mL IFNλ1-3. Fluorescent imaging was performed at 0 h, 12 h, 24 h, 48 h, 72 h, and 96 h post treatment. (**B**) Representative images at 0 h, 12 h, and 24 h post-treatment. The red line represents the edge of the patterns. Expression of the fluorescent reporter is depicted in white. Scale bar = 100 µm. (**C**) The radial distribution of immune response was determined for the 24 h post-treatment. Each population was segmented in rings (14 µm radius) and the mean fluorescence intensity (MFI) was measured within these rings. The MFI was normalized to the mock-treatment MFI of the corresponding ring. Each dot is one cell population (seeded on one micropattern). (**D**) The reporter expression for each single population was quantified by measuring the MFI at the edge and the center of a population at 12, 24, 48, 72, and 96 h post-treatment, and normalizing it to the mock-treatment of the respective time-point. Each dot is one cell population (seeded on one micropattern), lines connect edge and center of the same cell population. (**C**, **D**) *n* ≥ 3 biological replicates, in (**C**) error bars indicate the standard deviation. n.s. = not significant. *P* < 0.05 *, *P* < 0.01 **, *P* < 0.001 ***, *P* < 0.0001 **** as determined by (**C**) RM one-was ANOVA using the edge (0–14 µm) as reference and (**D**) Paired *t* test. Source data are available online for this figure.

To fully address whether there is a correlation between a cell location within its population and the extent by which it responds to IFN, we need to establish standardized methods that allow us to control how many cells in a population are located at the center or edge of this population. For this, we exploited a micropatterning method enabling us to create cell populations of defined and uniform sizes. In this method, a glass surface is passivated with poly-L lysin/poly-ethylene glycol (PLL-PEG), an antifouling agent to which cells cannot adhere (Fig. 2A). A Quartz-Mask imprinted with transparent patterns is then overlaid on the passivated surface and illuminated with UV-light in the presence of ozone. As Quartz reflects light, the UV-light can only pass through the transparent areas, thereby depleting the PLL-PEG at discrete locations creating size- and shape-specific patterns on which cells can grow. Using this approach, cells can be grown on controlled micropatterns that provide cells with the same population context.

T84 cells were seeded on micropatterns. Visual observation of the rate of cell growth on these micropatterns did not reveal any differences compared to cells grown on non-micropatterned glass surfaces. Immunostaining of the tight junction protein Zonula occludens-1 (ZO1) confirmed that the T84 epithelial cells formed tight junctions when grown on micropatterns (Fig. EV2A). As expected, cells located at the edge of the pattern did not display ZO1-positive tight junctions as they are not fully embedded inside a cellular population (Fig. EV2A, red arrows). Immunostaining of the *trans* Golgi network (TGN) confirmed its asymmetric distribution toward the apical moiety of the cytosol (Fig. EV2B), which is typical for polarized epithelial cells (Rodriguez-Boulan and Macara, 2014).

**Figure EV2:**

Protein expression and protein localization in IEC populations seeded on micropatterns. T84 WT cells were seeded on micropatterns as populations and fixed. Immunostaining was performed against a variety of proteins. Cells were imaged with spinning disc microscopy at different focal-planes (Z-stack), and visualized as apical view (with z-projection) or transversal view (xy-projection). (**A**) Representative images showing ZO1 protein (green) and Phalloidin-647 (magenta) which stains for F-actin. Red arrows point out edge cells lacking ZO1 protein, and yellow arrows show localization of ZO1 and F-actin to the apical side of the populations. (**B**) Representative images showing the mitochondria marker Cytochrom C (CytC), Trans-Golgi Network (TGN) and ZO1 in epithelial cell populations along the Z-axis. (**A**, **B**) Cell nuclei were stained with DAPI (blue). Scale bar = 50 µm.

To address how cellular localization within these micropatterns affects the response of IECs to IFNs, T84-prom-Mx1-fp cells were seeded on micropatterned glass and treated with type I or III IFNs. Analysis of IFN response by fluorescence microscopy at 0, 12, and 24 h post-treatment showed that mostly cells located at the edge of the pattern responded to type I and type III IFN treatment (Fig. 2B). Importantly, we observed a clear spatial heterogeneity in responsiveness of cells to IFNs, with an increase in the magnitude of pMx1-GFP expression from the center towards the edge of the population (Fig. 2B). Unbiased quantification of this spatial heterogeneity in responsiveness at 24 h post-treatment confirmed our original observation that the more a cell is embedded in a cell population, the less a cell respond to IFNs (Fig. 2C). To ensure that this spatial heterogeneity in responsiveness was not due to different kinetics of IEC response to IFN treatment depending on their position within the population, we analyzed the response to IFNs at the edge and the center of the population over a period of 96 h. For all tested time-points, edge cells were significantly more responsive to IFNs compared to center cells (Fig. 2D). At 96 h post IFNλ1-3 treatment no difference between edge and center cells were observed, but this was due to the fact that at 96 h post type III IFN treatment, expression of ISG in IECs is almost back to basal levels. Together, our data strongly suggest that IECs located at the edge of a cell population respond more efficiently to IFNs compared to cells embedded within the cell population.

### Cellular density impacts response of IECs to IFN treatment

To address which part of the IFN-mediated signaling pathway is impaired in cells located at the center of a population, we compared the response of T84 cells seeded at high (205,000 cells/cm^2^) vs. low (27,000 cells/cm^2^) cellular density. At high density, cells form a continuous intact monolayer, in which each cell is in contact with neighboring cells from all sides, representing the center of a population (Fig. EV3A, right panel). On the contrary, at low cell density most of the cells can be considered “edge” cells as they are isolated or are part of small cellular colonies with at least one side lacking a neighboring cell (Fig. EV3A, left panel). To quantitatively verify that at high density most cells will be found in the center of the cell population while, at low density, most cells will be situated at the edge of a population, we employed our DBSCAN-CellX-based approach to determine the position of cells within a population unbiasedly (Fig. 1B). Results confirmed that at low density, most cells were isolated (single cell) or situated at the edge of a population and conversely, at high density, most cells were located in the center of the cell population (Fig. EV3B).

**Figure EV3:**

Cell seeding at high and low density. T84 WT cells were seeded at high and low density. (**A**) Representative images showing cell nuclei stained with DAPI (blue). Scale bar = 100 µm. (**B**) The DBSCAN-CellX App was used to determine the percentage of cells localized at the edge and the center for low and high density seeding conditions. *n* ≥ 3 biological replicates, error bars indicate the standard deviation.

**Figure 3:**
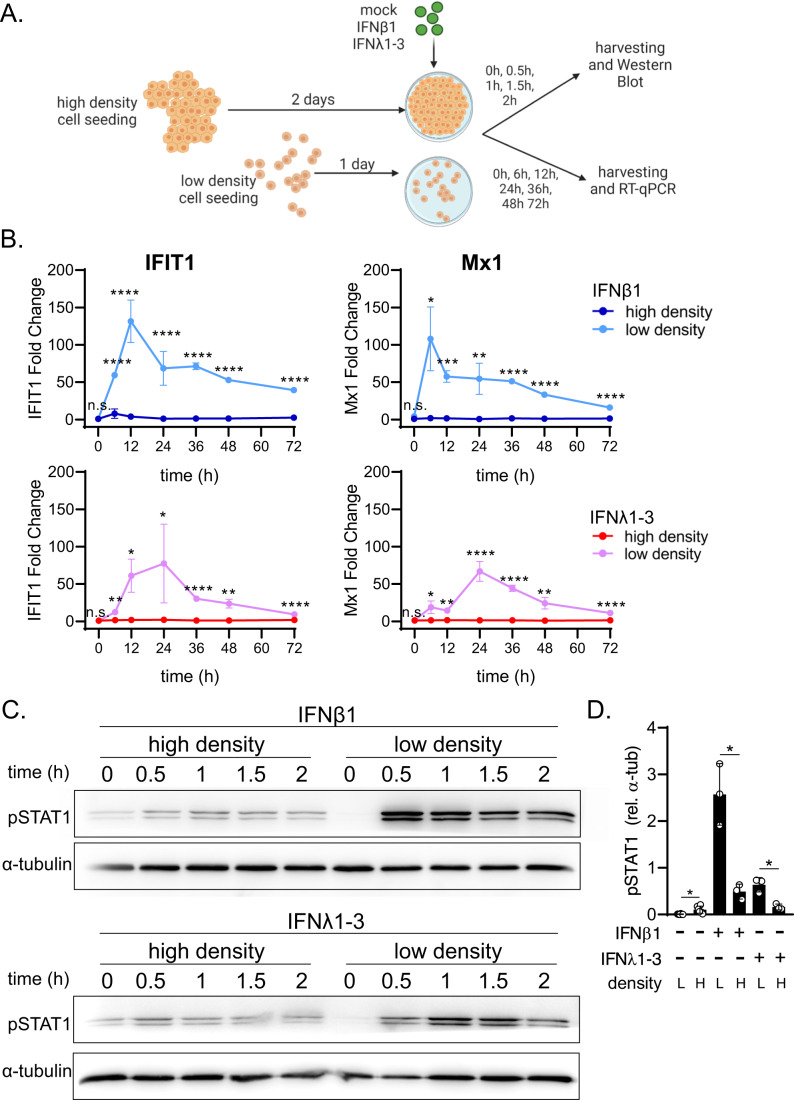
Cell density negatively correlates with IFN-dependent signaling. T84 cells seeded at high and low density were mock treated, or treated with 2000 IU/mL IFNβ1 or 300 ng/mL IFNλ1-3. (**A**) Schematic depicting the experimental setup. (**B**) At 0, 6, 12, 24, 48, 36, and 72 h post IFN treatment, RNA was harvested to evaluate the transcription of the representative ISGs IFIT1 and Mx1 using RT-q-PCR. ISG relative expression was normalized to the mock-treated cells of the respective time-point (fold change). (**C**, **D**) At 0, 0.5, 1, 1.5, and 2 h post treatment, cellular protein extracts were collected to assess the phospho-STAT1 (pSTAT1) abundance by Western Blot. (**D**) For the 1 h post-treatment samples, pSTAT1 was quantified relative to the housekeeping protein α-tubulin. (**B**, **D**) *n* = 3 biological replicates, error bars indicate the standard deviation. n.s. = not significant. *P* < 0.05 *, *P* < 0.01 **, *P* < 0.001 ***, *P* < 0.0001 **** as determined by Unpaired *t* test with Welch’s correction. Source data are available online for this figure.

T84 cells seeded at high and low density were treated with IFNβ1 or IFNλ1-3 (Fig. 3A). The IFN-mediated expression of the ISGs IFIT1 and Mx1 were measured overtime post-IFN treatment using reverse transcriptase quantitative PCR (RT-q-PCR) (Fig. 3B). Analysis revealed that cells at low density induce significantly higher ISG transcription compared to cells seeded at high density for all time-points, with almost no transcriptional upregulation of ISGs in cells seeded at high densities (Fig. 3B). These results were confirmed using our T84-prom-Mx1-fp reporter cell line. T84 prom-Mx1-fp cells expressing an H2B-turquoise plasmid (to visualize cell nuclei) were seeded at high and low density and treated 24 h post-seeding with increasing concentrations of IFNβ1 or IFNλ1-3. Following treatment, single cell expression of the fluorescent reporter was followed using live fluorescence imaging for 24 h (Fig. EV4A). Interestingly, we observed a dose dependent response to both IFN treatments in cells seeded at low density but not in cells seeded at high density (Fig. EV4B,C). Importantly, cells seeded at high density only responded minimally to both IFN treatments independently of the concentrations used (Fig. EV4B,C). These results mirror the intrinsic ISG expression levels as measured using RT-q-PCR (Fig. 3B) further demonstrating that the population context significantly impacts the response of IECs to IFN treatment.

**Figure EV4:**

Temporal response of cells at high and low density to IFN treatment. T84-prom-Mx1-fp cells at high or low density were treated with increasing concentrations of IFNβ1 and IFNλ1-3. Live cell fluorescence imaging was performed at an interval of 2 h for 24 h. (**A**) Representative images for selected time-points showing expression of the reporter prom-Mx1-mCherry (pMx1-mCh) in white. Nuclei are visualized by expression of H2B-turqiouse. Scale bar = 100 µm. (**B**, **C**) The mean fluorescence intensity (MFI) of the reporter expression within each cell was averaged for each density and normalized to the mock MFI of each time-point (fold change) for (**B**) IFNβ1 and (**C**) IFNλ1-3. *n* = 3 biological replicates. n.s = not significant, error bars indicate the standard deviation. *P* < 0.05 *, *P* < 0.01 **, P < 0.001 ***, *P* < 0.0001 **** as determined by Unpaired *t* test with Welch’s correction between high and low density for each time-point.

**Figure EV5:**
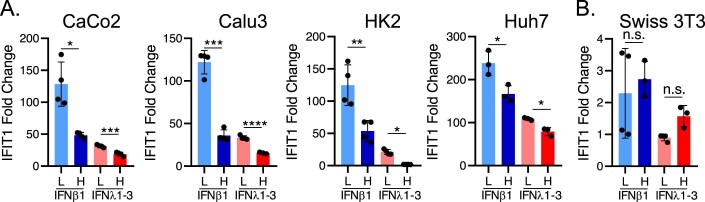
Effect of density and basolateral IFN receptor localization in epithelial and non-epithelial cells. (**A**) Epithelial and (**B**) non-epithelial cells were seeded at high (H) and low (L) density. Cells were mock treated, or treated with 2000 IU/mL IFNβ1 or 300 ng/mL IFNλ1-3. 24 h post IFN treatment, RNA was harvested to evaluate the transcription of the representative ISGs IFIT1 using RT-q-PCR. ISG relative expression was normalized to the mock-treated cells (fold change). *n* ≥ 3 biological replicates, error bars indicate the standard deviation. n.s. = not significant. *P* < 0.05 *, *P* < 0.01 **, *P* < 0.001 ***, *P* < 0.0001 **** as determined by Unpaired *t* test with Welch’s correction.

To control that the impact of the population context on response to IFN treatment was not specific to our T84 epithelial cells, we compared the response from other cells of epithelial origin. Interestingly, we observed that all tested epithelial cells (colon derived CaCo2 cells, lung derived Calu3 cells, kidney derived HK2 cells, and hepatoma derived Huh7 cells) responded significantly less to both IFNs when seeded at high density compared to when seeded at low density (Fig. EV5A). Importantly, cells from non-epithelial origin (mouse-derived fibroblast Swiss 3T3 cells) showed no difference in their response to IFN treatment between cells seeded at high and low densities (Fig. EV5B). Together, these results strongly suggest that cell confluency renders specifically epithelial cells less responsive to IFN treatment.

The first step in IFN-mediated signaling is binding of IFN to its receptor, which induces the activation of JAK1 that in turn phosphorylates STAT1/STAT2 (Schindler et al, 2007). To address whether cell density can impact the phosphorylation of STATs following IFN stimulation, T84 cells seeded at low and high densities were stimulated with either type I IFN (IFNβ1) or type III IFN (IFNλ1-3). At different time-points post stimulation, the phosphorylation status of STAT1 was addressed by Western Blot analysis. For all time-points, treatment of IECs seeded at low density with either type of IFN induced higher STAT1 phosphorylation as compared to cells seeded at high density (Fig. 3C). Quantification of the amount of phosphorylated STAT1 (pSTAT1) confirmed that sparse cells are significantly more responsive to both IFNs than confluent cells (Fig. 3D). Altogether, we observed a negative correlation between cell density and response to IFNs: confluent cells are almost unresponsive to IFNs while sparse cells show high levels of STAT1 phosphorylation and downstream ISG expression upon IFN treatment.

### Basolateral treatment of IECs with IFN suppresses the spatial heterogeneity of IFN-mediated signaling

**Figure 4:**
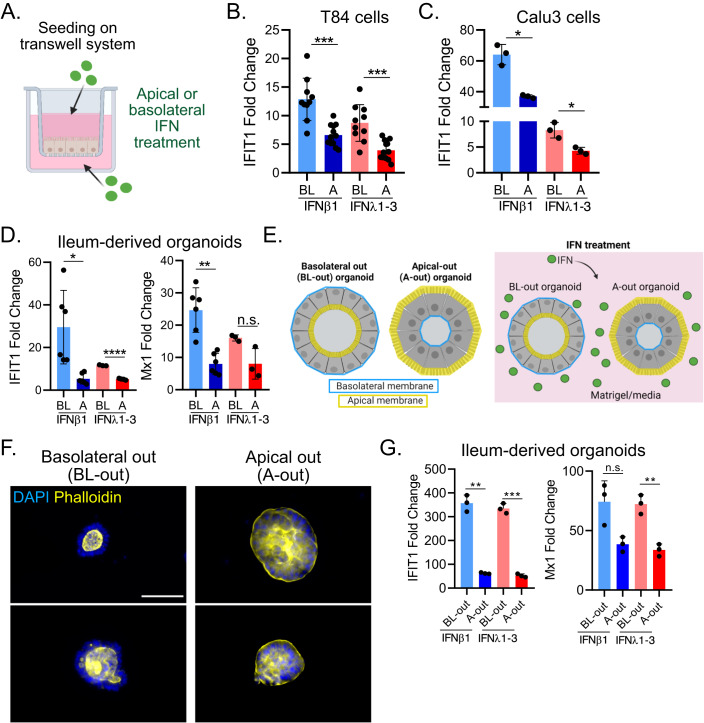
Epithelial cell lines and primary IECs respond better to IFN when stimulated from their basolateral side. (**A**–**D**) T84, Calu3, and human ileum-derived organoids seeded on transwell inserts were mock treated or treated from the apical (A) or basolateral (BL) side with 2000 IU/mL IFNβ1 or 300 ng/mL IFNλ1-3. (**A**) Schematic depicting the treatment of cells seeded on transwell inserts. (**B**–**D**) 24 h post treatment, RNA was harvested, and RT-q-PCR was used to evaluate the expression of the ISG IFIT1 and Mx1 for (**B**) T84 cells, (**C**) Calu3 cells, and (**D**) human ileum-derived organoids. Data is normalized to mock (fold change). (**E**–**G**) Organoids were grown in three-dimensionsional (3D) structures, either in a basolateral out (BL-out) or an apical out (A-out) conformation. (**E**) Schematic depicting BL-out and A-out organoids, and which membrane interacts with the IFNs during treatment. (**F**) BL-out and A-out organoids were stained for actin using Phalloidin (yellow), to mark the apical membrane of cells. Nuclei were stained with DAPI (blue). Scale bar = 100 µm. (**G**) BL-out and A-out organoids were treated with 2000 IU/mL IFNβ1 or 300 ng/mL IFNλ1-3. 24 h post treatment, RNA was harvested, and RT-q-PCR was used to evaluate the expression of the ISGs IFIT1 and Mx1. Data are normalized to mock (fold change). (**B**–**D**, **G**) *n* ≥ 3 biological replicates, error bars indicate the standard deviation. n.s. = not significant. *P* < 0.05 *, *P* < 0.01 **, *P* < 0.001 ***, *P* < 0.0001 **** as determined by Unpaired *t* test with Welch’s correction. Source data are available online for this figure.

**Figure EV6:**
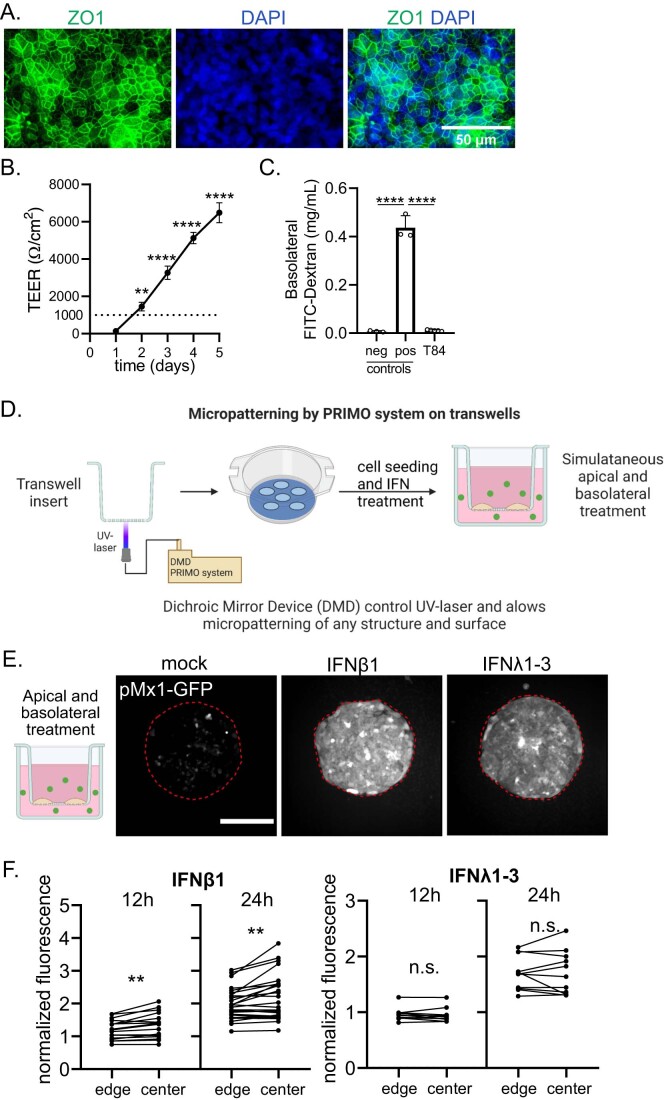
Transwell system to grow a semipermeable monolayer of polarized cells and to seed populations that are accessible from the basolateral side. (**A**–**C**) T84 cells were seeded on transwell inserts to allow for a polarized monolayer formation. (**A**) 5 days post seeding, cells were fixed, and indirect immunofluorescence was performed against the junctional complex protein ZO1 (green). Nuclei were stained with DAPI (blue). Representative image is shown. Scale bars = 50 µm. *n* = 3 biological replicates. (**B**) Formation and integrity of the monolayer was followed by measuring the transepithelial electrical resistance (TEER) (Ω/cm^2^) over 5 days. Values > 1000 Ω/cm^2^ (dotted line) shows that cells established a polarized monolayer formation. *n* = 3 biological replicates. (**C**) 5 days post seeding, after reaching a polarized monolayer, the integrity of the monolayer was confirmed by the FITC-Dextran permeability assay. Diffusion of FITC-Dextran from the apical to the basolateral compartment was measured and expressed as concentration (mg/mL) of FITC-Dextran in the basolateral compartment after 3 h incubation. Positive control (pos) was the maximum diffusion possible and the negative control (neg) was medium only without FITC-Dextran. (**D**) Micropatterning of transwell inserts: Schematic depicting the micropatterning on transwell membranes using the PRIMO system (Alvéole Lab, www.alveolelab.com). (**E**, **F**) T84 prom-Mx1-fp cells were seeded on micropatterned transwell membranes. Cells were mock treated, or treated simultaneously from the apical and basolateral side with 2000 IU/mL IFNβ1 or 300 ng/mL IFNλ1-3. Cells were fixed at 0 h, 12 h, and 24 h post treatment and fluorescent imaging was performed. (**E**) Representatives images showing treated T84 cell populations. The red line represents the edge of the patterns. Expression of the fluorescent reporter is depicted in white. Scale bar = 100 µm. (**F**) The reporter expression was quantified by measuring the mean fluorescence intensity (MFI) at the edge and the center of a population at 12 h or 24 h post treatment, and normalizing it to the corresponding 0 h post treatment at the edge and center, respectively. Each dot is one cell population (seeded on one micropattern), lines connect edge and center of the same cell population. (**B**, **C**, **F**) *n* ≥ 3 biological replicates, in (**B**, **C**) error bars indicate the standard deviation. n.s. = not significant, *P* < 0.05 *, *P* < 0.01 **, *P* < 0.001 ***, *P* < 0.0001 **** as determined by (**B**, **C**) ordinary one-way ANOVA with Dunnett’s multiple comparison test using (**B**) day 1 or (**C**) the positive control as reference, and (**F**) Paired *t* test.

IECs are polarized, meaning that they have both an apical and a basolateral membrane (Fig. 4A). The basolateral side represents the bottom of the cell, which is in contact with the cell culture vessel in vitro and in contact with the *lamina propria* in vivo. The apical membrane represents the top of the cell facing the cell culture medium in vitro and the lumen of the gut in vivo. A possible explanation to account for the greater response to IFNs of IECs located at the edge of a population of IECs seeded at low density could be if the IFN receptors are mostly localized on the basolateral side of IECs. IFN treatment of cells seeded on glass or plastic surfaces would not lead to IFN-mediated signaling for the cell located at the center of a population or in a confluent monolayer as IFNs would not be able to access the basolateral side of the cells where the IFN receptors may be localized. On the contrary, IFNs can stimulate cells located at the edge of a colony as edge cells are not polarized (Cao et al, 2012) and likely do not show an asymmetric basolateral distribution of their receptors. To directly challenge this model, we seeded T84 cells on transwell inserts to allow for the formation of a polarized cell monolayer characterized by the formation of tight junctions. Polarization and formation of tight junctions preventing diffusion of molecules across the epithelium barrier was controlled by immunofluorescence staining of the tight junction belt using an antibody against the tight junction protein ZO1, monitoring of the trans-epithelial electrical resistance (TEER), and restriction of FITC-dextran free diffusion from the apical to the basolateral transwell compartment (Fig. EV6A–C). As expected, T84 cells grown on transwell inserts formed a tight junction belt between individual epithelial cells (Fig. EV6A), established a TEER-value characteristic of polarized T84 cells (Fig. EV6B) (Benson et al, 2013), and formed a tight monolayer of cells that prevents diffusion of molecules between cells (Fig. EV6C). Polarized IECs on transwell inserts were treated with IFNs either from the apical or basolateral side for 24 h (Fig. 4A). RT-q-PCR analysis of the expression of the ISG IFIT1 showed that basolateral treatment of cells with IFNs induced a significantly higher response as compared to apical treatment (Fig. 4B). Similar results were obtained with lung derived Calu3 cells polarized on transwell inserts (Fig. 4C).

To address whether this polarized response to IFN treatment also takes place in primary human epithelial cells, we employed ileum-derived organoids and seeded them on transwell inserts. Basolateral IFN treatment resulted in a more pronounced expression of ISGs compared to apical treatment (Fig. 4D), suggesting that IFN-dependent signaling is also preferentially induced from to the basolateral membrane in primary cells. To conclusively demonstrate this, we cultured organoids in matrigel as three-dimensional (3D) structures. Organoids cultured by traditional 3D protocols grow in a “basolateral-out” (BL-out) conformation, in which the apical membrane is facing inwards towards the organoid lumen, while the basolateral membrane faces the matrigel and is therefore in contact with the culturing/treatment media (Fig. 4E). However, a recent study developed a method to reverse the polarity of classical organoids to grow them “apical-out” (A-out) (Sato et al, 2011). In this system, the apical membrane is in contact with the culturing/treatment media while the basolateral membrane faces inwards (Fig. 4E). First, we confirmed that polarity was reversed in apical-out ileum-derived organoids by actin staining using Phalloidin-647 (Fig. 4F) and then treated A-out and BL-out organoids with IFN (Fig. 4E, right panel). Excitingly, type I and III IFN treatment of BL-out organoids induced a significantly higher ISG expression than treatment of A-out organoids (Fig. 4G).

To address whether basolateral treatment of cells seeded on micropatterns can render the center cells responsive to IFNs, we micropatterned transwell inserts (Fig. EV6D) and seeded our T84-prom-Mx1-fp reporter cell line on them. With this technique the cells will grow at restricted (micropatterned) areas, leaving the rest of the transwell with no cells on it. At those cell-free areas IFNs can freely diffuse between the apical to the basolateral compartments. Therefore, within this setup the cells are simultaneously treated with IFNs from the apical and basolateral side. Analysis using fluorescent microscopy revealed that the prom-Mx1-fp reporter expression was not restricted to the edge cells anymore, but was instead also found at the center of the patterns (Fig. EV6E). Quantification of the fluorescent signal relative to mock treated cells showed that center cells are inducing identical (IFNλs) or slightly higher (IFNβ1) Mx1 levels compared to edge cells (Fig. EV6F). This is opposite to when micropatterned cells were stimulated with IFNs only from their apical side (Fig. 2D). Our data show that the spatial restriction of immune response following IFN treatment can be bypassed by stimulating cells from their basolateral side. Together these findings show that epithelial cells better respond to IFN treatment from their basolateral side, suggesting that the IFN receptors might be enriched at the basolateral side of polarized epithelial cells.

### IFNAR2 and IL10RB are predominantly localized at the basolateral side of polarized T84 cells

**Figure 5:**
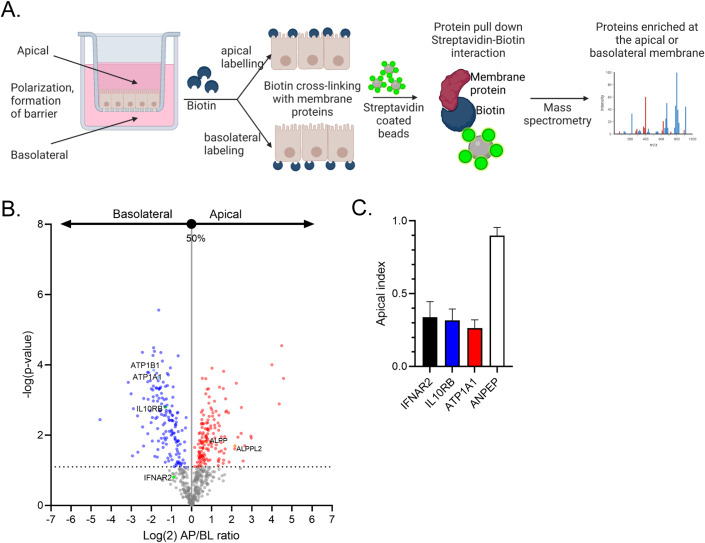
Mass spectrometry of the apical and basolateral proteome confirms the polarized localization of IFN receptors. T84 WT cells were grown as a polarized monolayer on transwell inserts. Apical or basolateral surface proteins were biotinylated by addition of cell non-permeable reactive NHS-biotin to the apical or basolateral compartment of the transwell insert, respectively. Biotinylated proteins were pulled down using streptavidin beads and identified using mass spectrometry. (**A**) Schematic showing the method. (**B**, **C**) The log(2) LFQ (Label-Free Quantification) signal between apical- and basolateral-biotinylated samples was calculated using pairwise *t*-tests coupled with sample randomization with false discovery rate (FDR) = 0.05, *n* = 4 biological replicates. (**B**) Volcano plot showing apical/basolateral log_2_ ratios for detected surface proteins in T84 cells. IFNAR2 and IL10BR are both present on the basolateral side of polarized T84 cells. Known apical and basolateral markers of polarized gut epithelial cells are also highlighted. (**C**) The apical index, indicating the percentage of protein enriched at the apical membrane, was calculated using following equation: (Apical LFQ value)/(Apical LFQ value + Basolateral LFQ value). Apical index of IFNAR2, IL10BR, the basolateral protein ATP1A1 and the apical protein ANPEP. Error bars indicate the standard deviation.

**Figure EV7:**
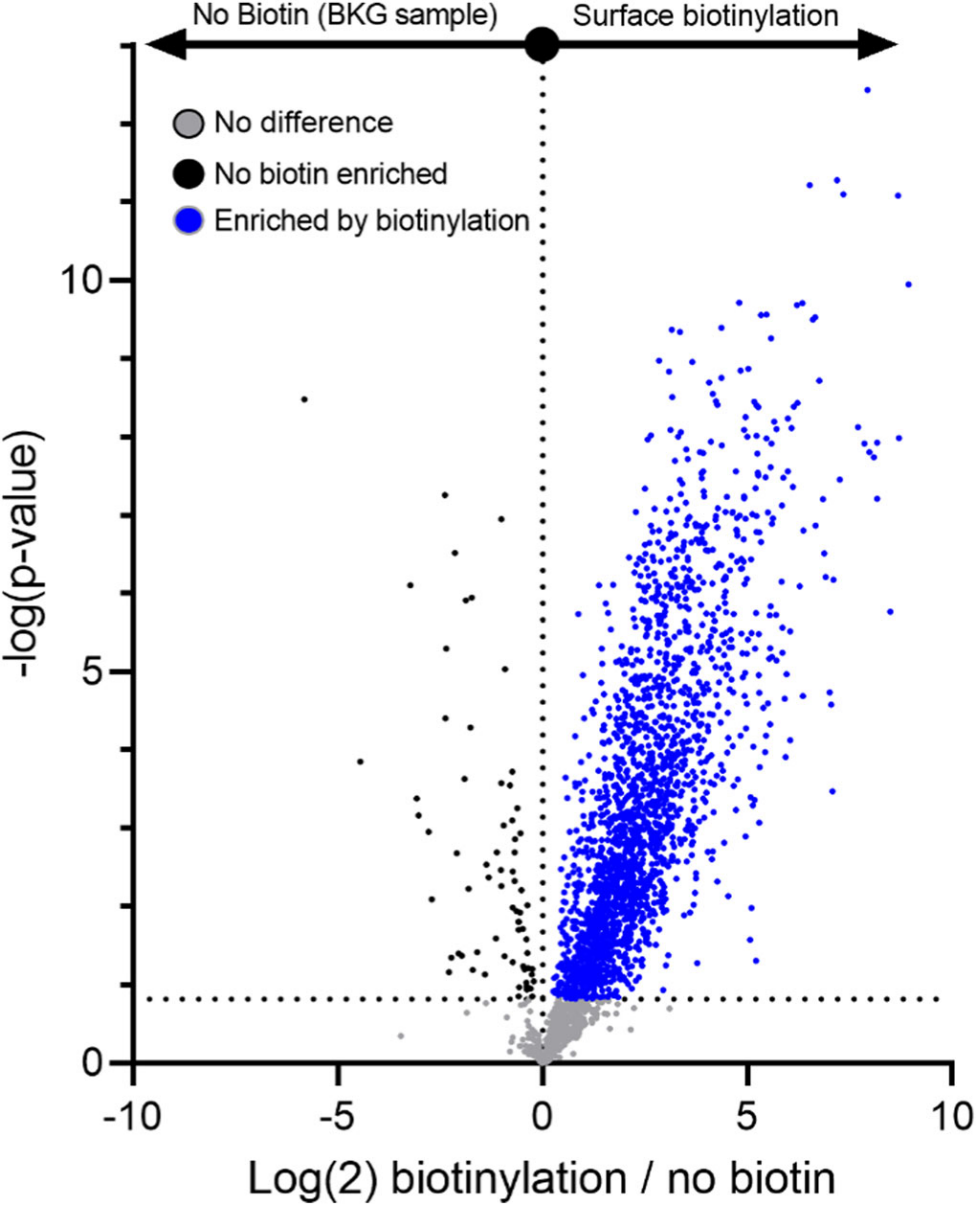
Enrichment via biotinylation of the surface proteome. T84 WT cells were grown as a polarized monolayer on transwell inserts. Apical or basolateral surface proteins were biotinylated by addition of cell non-permeable reactive NHS-biotin to the apical or basolateral compartment of the transwell insert, respectively. Biotinylated proteins were pulled down using streptavidin beads and identified by mass spectrometry. For the analysis, biotinylated proteins were filtered by using a non-biotinylated control sample by the volcano plot function of the Perseus software, where Biotinylated apical and Biotinylated basolateral samples are matched against the non-biotinylated control using a *t*-test with FDR of 0.05 and an s0 = 0.1. Volcano plot showing enrichment via biotinylation of the surface proteome. *n* = 4 biological replicates.

To directly address whether the IFN receptors are asymmetrically distributed in polarized T84 cells and located at their basolateral side, T84 cells were grown as a monolayer on transwell inserts. Apical or basolateral surface proteins were biotinylated by addition of cell non-permeable reactive NHS-biotin to the apical or basolateral compartment of the transwell inserts, respectively. Biotinylated proteins were pulled down using streptavidin beads and identified using mass spectrometry (Fig. 5A) (raw data available in Dataset EV1 and at ProteomeXchange with identifier PXD047936). To analyze the polarized enrichment of membrane proteins, first biotinylated proteins were filtered by using a non-biotinylated control sample. Mass spectrometry results showed a significant enrichment of biotinylated surface proteins, while non-specific binding to beads was minimal (Fig. EV7), thereby confirming efficiency of the pulldown. The log(2) LFQ (Label-Free Quantification) signal between apical- and basolateral-biotinylated samples was then calculated using pairwise *t*-tests coupled with sample randomization with false discovery rate (FDR) = 0.05, and visualized in a volcano plot as Log(2) AP/BL ratio (Fig. 5B). Moreover, the apical index was calculated using following equation: (Apical LFQ value)/(Apical LFQ value + Basolateral LFQ value) (Fig. 5C). The apical index indicates the percentage of the protein enriched at the apical membrane.

We controlled the specificity of our assay by determining the correct localization of known apical (ALPP and ALPPL2) and basolateral (ATP1B1 and ATP1A1) polarized intestinal epithelial cells markers (Fig. 5B highlighted in green and Fig. 5C). Analysis of the surface proteome of the apical and basolateral membranes of IECs revealed that both, IFNAR2 and IL10RB, were readily detectable at the surface of T84 cells with a specific distribution of 75% basolateral vs. 25% apical (Fig. 5B,C). The *p*-value for IFNAR2 was found to be not significant (Fig. 5B), which is probably due to the low protein expression level of IFNAR2 and the method detection limit, leading to high variability within the replicates. Importantly, while the protein was only detected at the apical membrane in 2 out of 4 total replicates, all replicates showed IFNAR2 at the basolateral membrane. IFNAR1 and IFNLR1 were not detectable using mass spectrometry likely due to their low expression levels. The distribution of IFNAR2 and IL10RB is in agreement with our findings where we stimulated T84s with IFNs from either the apical or basolateral membrane (Fig. 4). Together our data strongly suggest a model where the spatial restriction of IFN response to cells located at the edge of a cellular colony is due to the distribution of at least one subunit of the IFN receptors (IFNAR2 and IL10RB) mostly at the basolateral side of T84 cells.

### Tight junctions in polarized T84 cells restrict IFN access to their basolateral receptors

**Figure 6:**
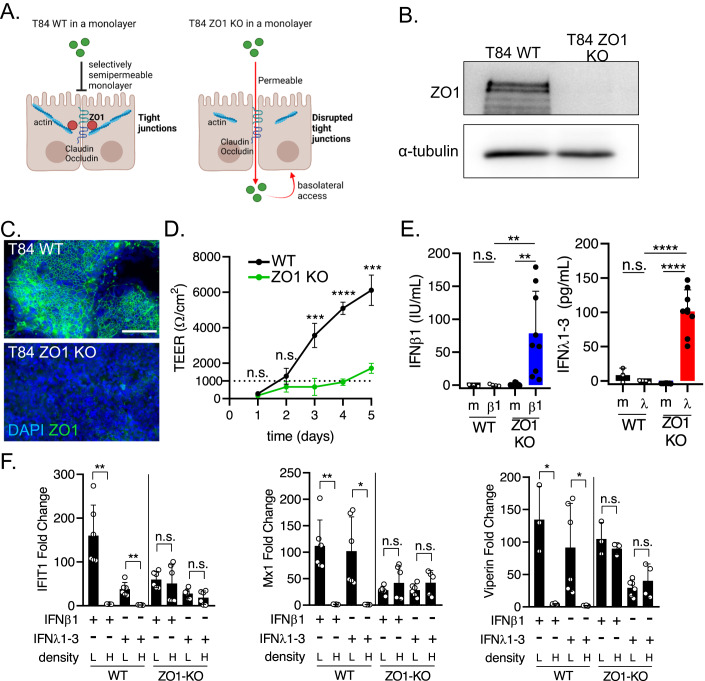
Disruption of tight junctions allows for IFN response in cells at high density. (**A**) Schematic depicting paracellular diffusion in a monolayer of T84 WT and T84 ZO1 KO cells with disrupted junctional complexes. (**B**) T84 WT and T84 ZO1 KO cell protein extracts were harvested to control the absence of ZO1 protein in the KO cells by Western Blot. α-tubulin served as a housekeeping protein. Representative image is shown. (**C**) T84 WT and T84 ZO1 KO cells were fixed and indirect immunofluorescence was performed against the junctional complex protein ZO1 (green). Nuclei were stained with DAPI (blue). Representative image is shown. Scale bar = 100 µm. (**D**) T84 WT and T84 ZO1 KO cells were seeded on transwell inserts and grown as a polarized monolayer. Transepithelial electrical resistance (TEER) was measured over a period of 5 days. Dotted line shows a TEER of 1000 Ω/cm^2^ corresponding to the resistance formed by confluent polarized T84 cells (Stanifer et al, 2016). (**E**) T84 WT and ZO1 KO cells were seeded on transwell inserts and grown as a dense monolayer, in which T84 WT cells reached a TEER > 1000 Ω/cm^2^. Cells were treated apically with 2000 IU/mL IFNβ1 and 300 ng/mL IFNλ1-3. 3 h after treatment, medium in the basolateral compartment was retrieved and IFN amount diffused from the apical to the basolateral transwell compartment was assessed by the HEK-blue assay. Depicted is the IFN concentration detected in the basolateral compartment for mock (m), IFNβ1 (β1) and IFNλ1-3 (λ) treated samples. (**F**) T84 WT and T84 ZO1 KO cells at high (H) and low (L) density were treated apically with 2000 IU/mL IFNβ1 or 300 ng/mL IFNλ1-3. 24 h post treatment, RNA was harvested, and RT-q-PCR was used to evaluate the expression of the ISG IFIT1. Data is normalized to mock (fold change). (**D**–**F**) *n* ≥ 3 biological replicates, error bars indicate the standard deviation. n.s. = not significant, *P* < 0.05 *, *P* < 0.01 **, *P* < 0.001 ***, *P* < 0.0001 **** as determined by (**D**) multiple *t* tests using the Bonferroni-Dunn method and analyzing WT and ZO1 KO conditions for each time-point individually, and (**E**, **F**) Unpaired *t* test with Welch’s correction. Source data are available online for this figure.

Tight junctions control paracellular permeability (Fig. 6A, left panel), and ZO1 has a central role as a scaffold protein within junctional complexes (Hartsock and Nelson, 2008). To address whether tight junctions can prevent the diffusion of IFN to the basolateral side of T84 cells when grown as a polarized monolayer, we created a cell line depleted of the master tight junction protein ZO1. We reasoned that knock-out of ZO1 should disrupt tight junctions, leading to uncontrolled paracellular diffusion between cells, allowing IFN to access the basolateral receptors (Fig. 6A, right panel). Knock-out of ZO1 was validated at the protein level by Western Blot analysis (Fig. 6B) and immunofluorescence staining (Fig. 6C). Importantly, depletion of ZO1 impaired the formation of a tight monolayer as ZO1 KO cells were significantly impaired in their establishment of a TEER when seeded on transwell inserts as compared to WT cells (Fig. 6D). To confirm that tight junctions control paracellular diffusion of IFNs, T84 WT and ZO1 KO cells were seeded on transwell inserts until WT cells polarized as measured by TEER. Cells were treated apically with 2000 IU/mL IFNβ1 or 300 ng/mL IFNλ1-3. 3 h post-treatment, the medium from the basolateral side of the transwell compartment was retrieved to measure the amount of IFNs that diffused across the cell monolayer from the apical to the basolateral side of the cells using the HEK-blue assay. While no IFN was detected in the basolateral transwell compartment for T84 WT cells, type I and III IFNs were found to have paracellularly diffused across the T84 ZO1 KO cell monolayer and could be detected in the basolateral compartment (Fig. 6E). These data show that tight junctions between polarized T84 cells block the paracellular diffusion of IFNs.

To directly address whether this blockade of IFN paracellular diffusion is responsible for the lack of response of confluent cells to IFN treatments, T84 WT and T84 ZO1 KO cells seeded at high (H) and low density (L) were treated apically with IFNs. In line with previous results, IFN treatment of sparse T84 WT cells induced significantly higher ISG expression as compared to IFN treatment of confluent T84 WT cells (Fig. 6F). In stark contrast, no difference could be observed in the response to IFNs at low vs. high density in T84 cells depleted of ZO1 (Fig. 6F). These results demonstrate that the tight junction protein ZO1 restricts the paracellular diffusion of IFN between polarized T84 cells, and that access of IFN to the basolateral membrane of a polarized T84 cellular monolayer is a crucial determinant to induce an IFN-mediated response.

### IEC density significantly affects the IFN-induced protection from virus infection

When performing traditional 2D cell culture experiments, seeding densities are chosen traditionally around 70% confluence or slightly adapted to accommodate for extended culturing times. On the contrary, when working with epithelial cells, high cell density is often employed to better mimic the physiological growing conditions of these cells and to induce cell polarization. Given the localization of the IFN receptors at the basolateral side of T84, we wondered whether cellular density can impact the outcome of viral infection during prophylactic treatment of epithelial cells with IFNs.

**Figure 7:**
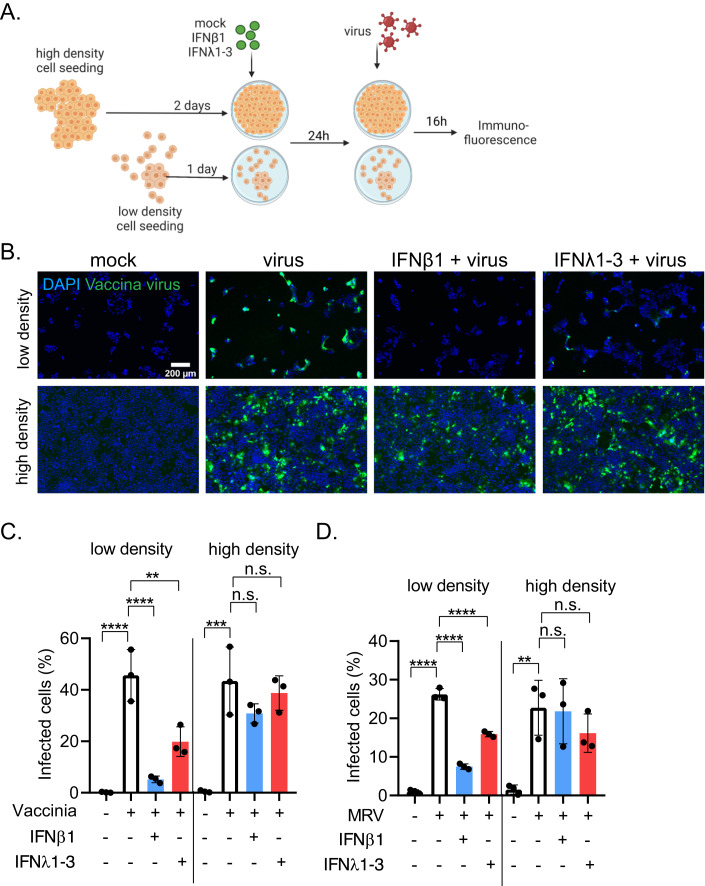
Polarized IFN receptor localization affects induction of an antiviral state in confluent cells. T84 cells seeded at high and low density were mock-treated or pre-treated with 2000 IU/mL IFNβ1 or 300 ng/mL IFNλ1-3. 24 h post treatment, cells were infected with Vaccinia virus-eGFP (VV) or Mammalian Reovirus (MRV) at an MOI of 1 (as determined in T84 WT cells). Infection media was supplemented with the respective IFN. 16 h post infection, cells were fixed, immunostained for viral protein and fluorescence imaging analysis was performed. (**A**) Schematic of the experimental setup. (**B**) Representative images showing Vaccinia virus eGFP (green) infected cells. Nuclei were stained with DAPI (blue). Scale bar = 200 µm. (**C**, **D**) Quantification of the number of (**C**) Vaccinia virus eGFP infected cells and (**D**) MRV infected cells. n = 3 biological replicates, error bars indicate the standard deviation. n.s. = not significant, *P* < 0.01 **, *P* < 0.001 ***, *P* < 0.0001 **** as determined by ordinary one-way ANOVA with Dunnett’s multiple comparison test. Testing was performed within high or low density groups, using only virus infected cells (no pretreatment) as reference. Source data are available online for this figure.

T84 cells were seeded at high and low density, and pre-treated with IFNβ1 and IFNλ1-3 for 24 h (Fig. 7A). Cells were then infected with two unrelated viruses, Vaccinia virus (VV) or Mammalian Reovirus (MRV), for 16 h and infection levels were assessed by immunofluorescence microscopy (Fig. 7A). Interestingly, IECs seeded at low cell density pre-treated with IFNs were able to control VV infection better than cells treated at high density (Fig. 7B). When quantifying the number of VV-infected cells, we observed that for both, high and low cell density, infection levels were around 50% (Fig. 7C). However, pre-treatment of cells seeded at low density with IFNβ1 strongly reduced the number of VV-infected cells to ~5%, and IFNλ1-3 reduced it to ~20% (Fig. 7C). In contrast, for cells seeded at high density, IFN pre-treatment had no significant effect on VV infection levels when compared to mock-treated infected cells (Fig. 7C). Similar results were observed for MRV infection, in which IFN pre-treatment of cells at low density significantly reduced the number of infected cells as compared to non-treated cells while no protective effect of IFN pretreatment was observed for cells seeded at high density (Fig. 7D). Together, we could show that the accessibility of the IFN receptor affects the antiviral priming of IECs. This has detrimental consequences for experimental outcomes, since cells at low confluence, where an antiviral state was induced, were able to restrict virus infection. On the contrary, cells at high confluence with less receptors accessible on the apical side, induced a lower response to IFN pre-treatment and therefore were not protected from virus infection.

Altogether, our results show that IECs differently respond to IFNs according to their population context. Isolated cells and cells located at the edge of a cellular colony are much more responsive to IFNs compared to cells embedded within a cellular colony. We could show that this spatial restriction of IFN-mediated signaling was due to the basolateral location of the IFN receptors in epithelial cells. Within a cellular colony, central embedded cells only have their apical plasma membrane accessible and as such respond very poorly to IFN treatment. Finally, we could show that this differential response of IECs to IFNs depending on the population context is critical to define whether IECs would be protected or not upon IFN treatment against viral infection. Our work highlights the importance of considering the population context when studying susceptibility of cells to viral infection and efficacy of antiviral measures, as the location of a cell within a population or whether the experimental set-up use partially vs. fully confluent cells can severely impact the experimental outcomes.

## Discussion

Determining at the molecular level how IFNs induce a protective antiviral state in IECs is a prerequisite to better understand infectious disease in the gut and to develop novel antiviral therapeutic strategies. Employing single cell and population analysis pipelines, we demonstrated that both, type I and type III IFNs induce a heterogeneous response in isogenic human IECs. This response is characterized by cells at the edge of a cellular colony mounting a significantly higher immune response as compared to cells localized in the center of the cell population. We identified that the origin of this cell-to-cell variability is an asymmetric distribution of the IFN-receptors toward the basolateral side of IECs. Cells localized in the center of a colony form a polarized monolayer, and IFNs coming from the cell culture medium (apical side) cannot access the basolateral receptors. On the contrary, cells at the edge of a colony are not polarized and the receptors are localized around the entire cell allowing interaction with IFNs present in the cell culture medium. In accordance with this observation, basolateral IFN treatment induced signaling in all cells within a population, independent on their location at the edge or the center of the colony. Importantly, this impact of the population context on the responsiveness of cells to IFN treatment was observed across multiple epithelial cells from different origins and in primary human intestinal cells (human ileum-derived organoids). Critically, we demonstrated that this polarized IFN-receptor localization can greatly affect the outcome of infection when addressing the protective state induced by IFNs during virus infection. Pre-treatment of confluent IECs with IFNs provides limited protection against viral infection. This finding highlights the importance of considering the population context when studying host cell pathogen interactions and when addressing the potency of the antiviral function of IFNs in epithelial cells.

### Heterogeneous response of cells within a population to IFN treatment

The heterogeneous response during IFN signaling in isogenic clonal cell populations has been widely observed. However, our understanding of the molecular basis for this cell-to-cell heterogeneity is only at its infancy. It has been reported that individual cells within a population induce ISG expression at different times post type I IFN treatment (Schmid et al, 2015a; Maier et al, 2022). Moreover, low concentrations of type I IFNs are known to induce a heterogeneous pattern of ISG expression levels with highly responsive, less responsive, and non-responder cell subpopulations (Rand et al, 2012; Bhushal et al, 2017b; Maier et al, 2022; Schmid et al, 2015a). Importantly, a similar subpopulation of non-responsive cells was observed in conditions where cells were treated with saturating type I IFN concentrations (Schmid et al, 2015b; Bhushal et al, 2017b). Altogether this demonstrates that, within a clonal cell population, some cells, although fully equipped at the molecular level to respond to IFNs, are not responsive to these cytokines. This non-responsiveness of a subpopulation of cells to IFNs is not terminally determined. When the non-responder population is isolated and re-treated with type I IFNs, the same heterogeneous pattern of ISG expression (responder and non-responder cells) was induced, thereby excluding the existence of a stable fraction of unresponsive clones (Schmid et al, 2015b; Bhushal et al, 2017b).

Using in silico modeling and single cell data from the murine fibroblast cell line Swiss 3T3 and the hepatocyte-derived epithelial-like cell line Huh7-5, the cell-to-cell variability was explained by stochastic events rooted in ‘biochemical noise’ (Maier et al, 2022; Rand et al, 2012). To point out, in these studies single cell behavior was assessed mostly by flow cytometry, in which it is not possible to trace the spatial context of each cell (Maier et al, 2022; Rand et al, 2012). In line with previous studies, we also observed that an isogenic population of adherent human IECs treated with either type I or type III IFNs from the apical side results in a strongly heterogeneous immune response, ranging from highly responding to non-responder subpopulations (Figs. 1, 2 and EV1). By using tools that integrate the effect of spatial components in cell behavior, we identified that the population context of single cells (position of a cell with respect to its neighbors within a population) partially explains the heterogeneous response of epithelial populations to IFNs. Precisely, we observe that cells at the edge of a population are more responsive to IFNs compared to cells in the center of the population. Importantly, when evaluating the edge or center cell populations independently, we can observe that individual cells within the same population context display a very heterogeneous response to IFN. This suggests that, within a given population context, additional cell driven mechanisms participate in shaping the responsiveness of cells to IFN (e.g., stochastic events).

Importantly, multiple epithelial cell lines as well as primary human-derived mini-gut organoids showed the same heterogeneous pattern during IFN-signaling, in which confluent cells and cells embedded in population are less responsive as compared to edge and sparse cells. Contrary to this, non-epithelial cells did not show decreased responsiveness to IFNs upon confluency. This observation reinforces the notion that the here described density-dependent sensitivity to IFNs is conserved among cells that undergo polarization along the apical-basolateral axis. In parallel to stochastic events, we hereby propose that the polarized distribution of the IFN receptors to the basolateral side of epithelial cells is a deterministic factor that contributes to the observed spatial restriction of IFN response. Of note, this only applies to cells that polarize along the apical-basolateral axis and therefore have distinctive membrane composition. In line with this, we demonstrated that IFN signaling in the non-epithelial cell line Swiss 3T3 is not affected by the population context in the same way as epithelial cells (Fig. EV5B). Interestingly, a study by Bhushal et al (Bhushal et al, 2017b) also observed the presence of a non-responsive subpopulation upon type III IFN treatment in murine IECs. In this study the number of reactive cells increased upon cell confluence. They further demonstrated that cell polarization and the epigenetic status determine the size of the non-responder population, thereby explaining that the heterogeneous response to type III IFNs in mouse IECs is also partially dependent on the population context. This and our study thereby highlight the role of cell confluence and the population context during IFN sensing and signaling, which can be incorporated as a deterministic factor playing a role during cell-to-cell variability during IFN signaling in epithelial cells.

Although the responsiveness of epithelial cells in this study can be partially ascribed to the basolateral localization of IFN receptors, this explanation does not fully account for the observed variations in the amplitude of individual cell responses (reflected by the intensity of fluorescent ISG reporter expression) when subjected to apical and basolateral treatments (Figs. 1, 2, and Fig. EV1). Moreover, although within our model we anticipate that every edge and single cell would respond to apical IFN treatment, a considerable proportion of these cells still do not exhibit a response even at saturating dose of stimuli (Fig. 1). For example, around 20% of single cells did not respond to apical IFNβ1 treatment and 60% did not respond to apical IFNλ1-3 treatment (Fig. 1F). These observations strongly suggest that additional mechanisms drive a heterogeneous response in epithelial cells, which could originate from stochastic events as described before (Maier et al, 2022; Rand et al, 2012). For example, abundance of signaling molecules as the IFN receptor or STATs could play a role in making cells in a population refractory to IFN signaling. We suggest that, in addition to the population context, other factors, whether of deterministic or stochastic origin, influence cell-to-cell variability in isogenic epithelial cell populations.

With our study we identified the population context as an important parameter driving the response of epithelial cells to IFNs. We propose that the population context might also be an important driving parameter to be considered for cellular responses that are known to display heterogeneity between cells. This outlines that non-genetic-based heterogeneity can sometimes be largely explained by yet unmeasured differences in biology, and thus some biological processes could be more deterministic than initially thought.

### Spatial and temporal determinants of heterogeneity to extracellular stimuli

Studying cell-to-cell heterogeneity in isogenic populations has been facilitated by single-cell transcriptomic and flow-cytometry, however these methods do not integrate both the spatial and temporal determinants that may characterize responder and non-responder cells. In contrast, high content imaging enables the collection of spatially and temporally resolved data with single cell resolution. Here, we combined high-content imaging using a fluorescent reporter cell line with (a) a bioinformatics method (DBSCAN-CellX) (Fig. 1) and (b) a micropatterning method (Figs. 2 and EV6) to address how the population context (spatial heterogeneity) impacts IFN-mediated immune response and antiviral response in IECs. Using our recently developed DBSCAN-CellX approach (https://github.com/GrawLab/DBSCAN-CellX/) (Küchenhoff et al, 2023), we could quantify the relative location of individual cells within a population with respect to their neighboring cells (Fig. 1), providing us with a tool to address single cell behavior in their population context. This allowed us to identify a spatially-dependent heterogeneous response pattern, in which significantly more cells at the edge of a population induced ISG expression as compared to cells in the center of a population. These results were confirmed using our micropatterning approaches that allowed us to create cell populations in which all population context parameters (population size, local density, polarization status) were manipulatable and reproducible (Figs. 2 and EV6), enabling us to study cell population behavior in an unbiased and controlled manner. Using these tools in combination with high content imaging pipelines promise to improve our understanding of cell-to-cell heterogeneity and its origin.

### The population context impacts cell ability to mount an antiviral response upon IFN treatment

We demonstrated that cell confluence can greatly affect experimental outcomes while testing the sensitivity of several viruses to IFN treatment. To address whether IFNs are protective against those pathogens, we apically pre-treated cells at high and low density with IFNs prior to virus infection (Fig. 7). The conclusions that are drawn from these two different experimental setups (high vs. low cell density) are opposing: Results obtained from low density suggest that IFNs induce a strong antiviral state against the tested viruses. On the contrary, results from confluent cells show that IECs cannot be protected from viruses by IFNs. These experiments highlight the importance of considering cell density when determining the experimental setups, especially in the context of the intestinal epithelium and antiviral immune response. Previous studies demonstrated that cell density is involved in major cellular molecular pathways, and thereby affects lipid composition (Kavaliauskiene et al, 2014), endocytic events (Snijder et al, 2009) and the expression of central molecules including autophagy markers p62 and LC3II, lysosomal cathepsin D as well as nuclear proteins HDAC1 and Lamin B1 (Trajkovic et al, 2019). Interestingly, Trajkovic et al (Trajkovic et al, 2019) treated cells with widely used compounds that lead to undesired changes in cell density, and compared the effect of the compounds to non-density matched or density matched controls, which lead to ambiguous conclusions. This demonstrated that cell density is a potent experimental variable, and they emphasize that a rational experimental design including cell density controls will minimize erroneous interpretation of cell culture data. Importantly, cell density is assumed to be associated with drug resistance and various studies showed that cells embedded in a confluent monolayer are significantly less susceptible to drug treatment (Fang et al, 2007; Meli et al, 2012), which has far reaching effects in the area of drug screening and development within the biomedical industry. We propose that cell density is underestimated during the evaluation of results and must be more actively addressed when planning experiments. Moreover, joined effort must be invested in recognizing population factors involved in biological processes.

### Polarized distribution of IFN receptors

We demonstrate that both type I and type III IFN receptors are enriched on the basolateral membrane of polarized IECs. Polarized IFN-alpha receptor (Jaspers et al, 2009) and IFN-gamma receptor (Humlicek et al, 2007) localization to the basolateral membrane has been reported before for airway epithelial cells. However, to the best of our knowledge, no report has focused on IFN receptor localization in the gut. A polarized receptor localization might have a physiological relevance, since in vivo IECs are in contact with the *lamina propria* from the basolateral side, where immune cells are also situated. On the contrary, the apical membrane faces the gut lumen containing the commensal microbiota system. Sensing IFNs from the sterile basolateral side could be a mechanism to selectively sense IFNs provided by immune cells. IECs also express and secrete IFNs to act in an autocrine and paracrine manner, and to propagate an antiviral immune response. Interestingly and in line with our results, it was demonstrated that after virus infection of polarized human IECs in vitro, IFNλ was secreted predominantly to the basolateral side (Stanifer et al, 2016). Further studies must address whether IFN secretion in vivo by IECs occurs on the apical or basolateral side, and how this is relevant in the context of a basolateral IFN receptor localization.

With our study we provide a novel approach to understand the origins of heterogeneity in isogenic populations. We demonstrated that the spatial heterogeneity during IFN response in epithelial cells is originated by a basolateral receptor localization in polarized cells. As our results show, the population context determining the polarized receptor localization can have wide-ranging effects on the experimental outcome, and we suggest that experiments need to be planned accordingly to obtain accurate results.

## Methods

### Cell lines, cell culture media, and viruses

Wild type (WT) T84 (ATCC CCL-248) as well as T84 reporter and knock-out (KO) cells were cultured in a 50:50 mixture of Dulbecco’s Modified Eagle’s Medium (DMEM) and F12 (Gibco #11320033). Calu3 (ATCC #HTB-55), CaCo2 (ATCC #HTB-37), Swiss 3T3, HEK-blue™ IFN-α/β cells (Invivogen #hkb-ifnab) and HEK-Blue™ IFN-λ cells (Invivogen #hkb-ifnl) cells were grown in DMEM (Gibco #31965). Huh-7 cells were cultured in DMEM (Gibco #31965) supplemented with Non-Essential Amino Acids (Thermo Fisher Scientifi #11140050). HEK293T cells (ATCC #CRL-3216) were maintained in Iscove’s modified Dulbecco’s medium (IMDM) (Gibco #124400-053). HK-2 cells were grown in Keratinocyte SFM supplemented with 0.05 mg/mL bovine pituitary extract (BPE) and 5 ng/mL human recombinant epidermal growth factor (EGF) (Thermo Fisher Scientific #17005042). All media, except the HK-2 cell media, were additionally supplemented with 10% fetal bovine serum (Sigma Aldrich #12306 C) and 100 U/mL penicillin and 100 μg/mL streptomycin (Gibco #15140122). HK-2 cells were maintained in serum-free media without penicillin and streptomycin (only supplemented with BPE and EGF as specified above). All cell lines were authenticated by STR profiling and tested for mycoplasma contamination. Importantly, T84, Caco2, and Calu3 cells must be cultured on collagen coated surfaces. Plastic surfaces (including culturing flasks, multi-well plates, and transwell inserts) were coated with 0.01 mg/mL rat tail collagen (Sigma Aldrich #C7661). Glass surfaces (including glass coverslips and 8-well chamber slide) (IBIDI #80827)) were coated with 0.04 mg/mL human collagen (Sigma Aldrich #C5533) diluted in water.

The IFN-sensing reporter T84 cell lines expressing prom-Mx1-mCherry or prom-Mx1-eGFP were previously generated in our laboratory and described in Doldan et al (Doldan et al, 2022a). The T84 ZO1 KO cell line was generated using a lentivirus-based CRISPR-Cas9 gene editing system. First, the guideRNA with the sequence gttttagagctagaaatagcaagttaaaataaggctagtccgttatcaacttgaaaaagtggcaccgagtcggtgc was inserted into the plasmid lentiCRISPRv2 containing a Blasticidin resistance. A lentivirus vector system was used to efficiently deliver the CRISPR-Cas9 plasmid to the T84 cells. To first package the plasmid in lentivirus, HEK293T cells at 80% confluence in a 10 cm^2^ dish were transfected with 8 µg of the CRISPR-Cas9 plasmid containing the guideRNA targeting ZO1, 4 µg pMDG.2 plasmid and 4 µg psPAX plasmid by using the transfection reagent Polyethylenimine (PEI) (Polysciences #23966-100) at a PEI:DNA ratio of 4:1. Three days post transfection, the supernatant containing lentivirus was collected, spun down to separate it from cell debris at 4000 rcf for 10 min, and filtered through a 0.45 µm syringe filter (Lab Unlimited #W10462100). To pellet the lentivirus, the supernatant was spun down at 125,000 rcf for 1:40 h using a SW40 Ti rotor. The lentivirus pellet was resuspended in 100 µL OptiMem (Gibco #31985062) (per yield of one 10 cm^2^ dish) and used for transduction. For transduction, 300,000 WT T84 cells per well in a 6-well plate were treated with 20 µL lentivirus using 3 µL Polybrene transfection reagent (Sigma Aldrich #TR-1003-G) diluted in 3 mL media. After 3 days of incubation, transduced cells were selected with Blasticidin (0.1 mg/mL) (Invivogen #ant-bl-1). Single cell cloning was performed using a limited serial dilution approach to obtain a monoclonal population knocked out for ZO1.

Mammalian Reovirus (MRV) type 3 clone 9 was derived from stocks originally obtained from Bernard N. Fields and was grown and purified by standard protocols (Stanifer et al, 2016). Vaccinia virus eGFP is a Western Reserve Vaccinia Virus strain that expresses EGFP under the control of a synthetic Early/Late virus promoter and was first described by Mercer and Helenius (Mercer and Helenius, 2008). Vaccinia virus eGFP was kindly provided by Jason Mercer and was grown and purified by standard protocols (Cotter et al, 2015).

### Cell culture

Cell seeding on multiwell plates: For high density, 225,000 cells per well were seeded in 48-well plates. One day post-seeding, medium was exchanged with 0.5 mL fresh culturing medium, and two days post-seeding cells were treated with IFNs. For low density, 30,000 cells per well were seeded in 48-well plates. One day post-seeding cells were treated with IFNs.

Cell seeding on glass bottom 8-well chamber slides (IBIDI): 100,000 T84 cells per well were seeded on glass bottom 8-well chamber slides coated with 2.5% human collagen (Sigma #C5533-5MG) diluted in water. One day post seeding cells were treated with IFNs.

Cell seeding on transwell inserts: 120,000 cells were seeded on rat-collagen (Sigma-Aldrich #C7667-25MG) coated 6.5 mm transwell 3.0 µm Pore Polycarbonate Membrane Inserts (Corning, #3415). Media was exchanged every second day until a polarized cell monolayer was formed. Monolayer permeability and integrity was assessed by measurement of the Transepithelial electrical resistance (TEER) using the EVOM3 Epithelial Volt/Ohm Meter with STX2-PLUS (Word Precision Instruments). When a TEER of ≥1000 Ω/cm^2^ was reached, cells were considered polarized forming a tight monolayer.

### Human organoid cultures and ethical approval

Human tissue samples were obtained from ileum biopsies at the University Hospital Heidelberg with informed written consent from all participants in accordance with the Declaration of Helsinki. This research adhered to the guidelines of the University Hospital Heidelberg and all samples were collected and stored in an anonymized manner. The protocol received approval from the “Ethics Commission of the University Hospital Heidelberg” under the reference number S-443/2017. Ileum organoids derived from two different donors were established as described in (Sato et al, 2011). Briefly, tissues were dissociated using 2 mM EDTA and stem cell-containing crypts were separated and filtered through 70 µm filters (Greiner). The fractions containing the highest numbers of crypts were combined and seeded into Matrigel (Corning #354230). Organoid culturing and passaging was done as previously described (Stanifer et al, 2020). In short, organoid 3D structures were embedded in 100% Matrigel, grown in basal media (Table 1) at 37 °C in 5% CO_2_, and partial media changes were performed every 2 days. Organoids were subcultured depending on growth rate and size, typically they were split weekly in a 1:2–1:6 ratio.

**Table 1:** Compounds and concentrations for human organoid basal and differentiation media.

Basal media		Differentiation media	
Compound	Final concentration	Compound	Final concentration
Advanced DMEM/F12 + 2 mM GlutaMAX + 10 mM HEPES + 100 U/mL penicillin and 100 μg/mL streptomycin		Advanced DMEM/F12 + 1x GlutaMAX + 10 mM HEPES + 100 U/mL penicillin and 100 μg/mL streptomycin	
L-WRN cell conditioned supernatant (WNT, R-Spondin, Noggin)	62.5% (v/v)	R-Spondin cell conditioned supernatant (WNT, R-Spondin, Noggin)	10.5% (v/v)
B-27 Supplement	1x	B-27 Supplement	1x
EGF (recombinant mouse)	50 ng/mL	EGF (recombinant mouse)	50 ng/mL
A83-01	500 nM	A83-01	500 nM
IGF-1 (recombinant human)	100 ng/mL	IGF-1 (recombinant human)	100 ng/mL
FGF-basic (recombinant human)	50 ng/mL	FGF-basic (recombinant human)	75 ng/mL
Noggin (recombinant mouse)	25 ng/mL	Noggin (recombinant mouse)	50 ng/mL
Gastrin	10 nM	Gastrin	10 nM
N-acetyl-cysteine	1 mM		

### Interferon treatment

Human recombinant IFN-beta 1a (IFNβ1) was obtained from Biomol (#86421) and cells were treated with 2000 IU/mL or as described in the figure legend. Human recombinant IFNλ1 (IL-29) (#300-02L), IFNλ2 (IL28A) (#300-2K), and IFNλ3 (IL-28B) (#300-2K) were purchased from Peprotech, and cells were treated by a cocktail of all three type III IFNs in a ratio of 1:1:1, resulting in a final concentration of 300 ng/mL or as described in the figure legend. Cells were treated with IFNs diluted in culturing media (250 µL for 48-well plate, 200 µL for Labtec, 200 µL for apical transwell treatment, 800 µL for basolateral transwell treatment, 1 mL for patterned coverslips) and the duration of the treatment is stated in the figure legends.

### Western Blot

Cells were harvested and lysed with 1X RIPA buffer (150 mM sodium chloride, 1.0% Triton X-100, 0.5% sodium deoxycholate, 0.1% sodium dodecyl sulfate (SDS), 50 mM Tris, pH 8.0) with cOmplete™ Mini EDTA-free Protease Inhibitor Cocktail (Sigma Aldrich #11836170001) and phosphatase inhibitor PhosSTOP (Millipore Sigma #PHOSS-RO) for 5 min at 37 °C. Lysates were collected and protein concentration was measured using the Pierce BCA Protein Assay Kit assay (Thermo Scientific #23225) according to the manufacturer’s protocol. 8 µg protein per condition were separated by SDS-PAGE and blotted onto a 0.2 µm nitrocellulose membrane (Bio-Rad, #1704158) using a Trans-Blot® Turbo™ Transfer System (Bio-Rad). Membranes were blocked with Tris Buffer saline (TBS)-tween (0.5% Tween in TBS) containing 5% Bovine Serum Albumin (BSA) (blocking buffer) for 2 h at room temperature (RT). Primary antibodies against alpha-Tubulin (Sigma #T9026), phospho-STAT1 (BD Transductions #612233) and ZO1 (Invitrogen #33-9100) were diluted 1:1000 in the same blocking buffer and nitrocellulose membranes were incubated with the antibodies diluted in the blocking buffer overnight at 4 °C. Membranes were then washed three times with TBS-T for 5 min at room temperature (RT) while rocking. Anti-mouse antibodies coupled with horseradish peroxidase (HRP) (GE Healthcare #NA934V) were used at 1:5000 dilution in blocking buffer and incubated at RT for 1 h while rocking. Membranes were washed three times with TBS-T for 5 min at RT while rocking. The Pierce ECL Western Blotting Substrate (Fisher #32209) was used for detection according to manufacturer instructions. The nitrocellulose membrane was imaged with the ImageQuant™ LAS 4000 (GE Healthcare). Quantification was done using the open image analysis software ImageJ. Relative abundance of phospho-STAT1 was normalized to the loading control protein alpha-Tubulin.

### RNA isolation, cDNA synthesis, and RT-q-PCR

Cells were harvested at different times post IFN treatment, and RNA was isolated using RNAeasy RNA extraction kit (Qiagen) as per manufacturer’s instructions. DNA was synthesized using iSCRIPT reverse transcriptase (BioRad) from 250 ng of total RNA per 20 µL reaction according to the manufacturer’s instructions. Quantitative RT-PCR assay was performed using iTaq SYBR green (BioRad) as per manufacturer’s instructions. The expression of the various ISGs was normalized to the housekeeping gene *TBP* for human cells or *GAPDH* for mouse cells. The expression levels of the various ISG were then normalized to mock of each time-point, to obtain the fold change expression to mock treated cells. Primer sequences are listed below (Table 2).

**Table 2:** Primer sequences for RT-q-PCR.

Target gene	Species	Forward sequence	Reverse sequence
TBP	Human	CCACTCACAGACTCTCACAAC	CTGCGGTACAATCCCAGAACT
GAPDH	Mouse	AGGTCGGTGTGAACGGATTTG	GAAGATGGTGATGGGATTTC
IFIT1	Human	AAAAGCCCACATTTGAGGTG	GAAATTCCTGAAACCGACCA
IFIT1	Mouse	CAGCTACCACCTTTACAGCAACC	CCTGGTCACCATCAGCATTCT
Mx1	Human	GAGCTGTTCTCCTGCACCTC	CTCCCACTCCCTGAAATCTG
Viperin	Human	GAGAGCCATTTCTTCAAGACC	CTATAATCCCTACACCACCTCC

### Analysis of spatial heterogeneity using image analysis software

T84 prom-Mx1-eGFP cells seeded on glass bottom 8-well chamber slides (IBIDI) were mock treated or treated with IFNs for 24 h and fixed in 2% paraformaldehyde (PFA) (in PBS) for 20 min at RT. Cells were washed in 1X PBS and permeabilized in 0.5% Triton-X-100 (in PBS) for 15 min at RT. Cell nuclei were stained with DAPI (BD Biosciences #564907) diluted 1:1000 in PBS for 20 min. Cells were washed in 1X PBS three times and maintained in PBS. Cells were imaged on a ZEISS Celldiscoverer 7 Widefield microscope using a 20 ×0.5 magnification (Numerical Aperture NA = 0.5).

To analyze the spatial heterogeneity of IFN-dependent immune response, we first generated masks from DAPI images representing each nucleus as an individual object with the segmentation software Ilastik 1.2.0. These masks were then used in CellProfiler 3.1.9 to determine (a) the XY-localization of each object (nucleus) within its 2-dimensional plane and (b) to measure the prom-Mx1-eGFP fluorescence intensity within each object (nucleus). Using the information on the XY-localization, we applied the DBSCAN-CellX-App (https://github.com/GrawLab/DBSCAN-CellX/) (Küchenhoff et al, 2023) to the data to assess whether a cell is localized at the edge or the center of a cluster, and to determine the edge degree of a cell. The cell localization and cell edge degree were plotted against the percentage of prom-Mx1-eGFP positive cells (as compared to the mock-treated samples) within each sub-population group, resulting in the visualization of the immune response of single cells within their population context.

### Surface micropatterning, cell seeding, and image analysis

For glass micropatterning by Quartz mask-based approach (ultraviolet light-Ozone (UVO)-based micropatterning of glass surfaces using a Quartz-mask), a quartz chromium photomask containing 200 µm diameter clear circles was custom made by Toppan Photomasks Inc. (Mask type = 1X Master, mask size = 4” × 4” × 0.06”). The UVO-based micropatterning protocol was adapted from Pitaval et al (Pitaval et al, 2010). Briefly, glass coverslips of 25 mm diameter (Marienfeld # 0117650) were pre-cleaned with 100% ethanol for 15 min while sonicating, rinsed twice with deionized water, and dried with compressed air. The glass coverslips were activated in the UVO-Cleaner® Model 30 (Jelight Company Inc.) for 10 min and then passivated for 45 min at room temperature with 100 µL 0.1 mg/mL poly-L lysin/poly-ethylene glycol PLL(20)-g[3.5]-PEG(2) (SuSoS Surface Technology) in water. After passivation, the coverslips were washed twice with deionized water for 10 min. Before the micropatterning step, the photomask was washed with acetone and isopropanol, dried with a stream of compressed air and cleaned in the UVO cleaner for 5 min. Directly after cleaning, the passivated glass coverslips were sandwiched with the photomask using 8 µL of deionized water to create an intimate contact between the chromium side of the photomask and the passivated surface of the coverslip. The photomask with the coverslips was placed in the UVO cleaner (quartz side facing towards UVO light) for 5 min for the micropatterning step. After UVO exposure, the coverslips were carefully detached from the photomask and stored in PBS at 4 °C until further use.

Transwell micropatterning using maskless photolithography system (Fig. EV6): 6.5 mm Transwell® with 3.0 µm Pore Polycarbonate Membrane Inserts (Costar #CLS3415) were used. The transwell membrane was activated in the plasma cleaner (Tepla 100-E Plasma System) at 0.4 mbar O_2_-pressure and 200 W for 1 min. The surface was then incubated with 0.1% (w/v) Poly-L-Lysin (PLL) solution (in H_2_O) (Sigma #P8920) for 30 min at RT, washed four times with deionized water and dried with compressed air. The surface was passivated with 90 µL 90 mg/mL Methoxy-Poly (Ethylene Glycol)-Succinimidyl Valerate (mPEG-SVA) (5000 Da) (Laysan Bio Inc. #MPEG-SVA-5000) in 0.1 M HEPES buffer (pH 8.4) for 1 h at RT. During this reaction the SVA ester covalently binds to the amines of the PLL, resulting in a homogenous passivation of the glass surface with a PLL-PEG polymer. The surface was washed four times with deionized water and dried with compressed air. 0.5 µL photoactivator PLPP-gel (Alvéole Lab, www.alveolelab.com) was put in the center of the surface. Immediately after, 16 µl of 100% EtOH were added on the top of the PLPP-gel and the mixture was homogenized by manual rotation and the surface was dried at RT. This system is able to micropattern any previously designed pattern on any surface. To design a pattern, the open-source software Inkscape (inkscape.org) was used with the following scale: 1 px corresponded to 0.28 µm. We designed circles with 200 µm diameter, which was then loaded into the Leonardo software (Alvéole Lab) for micropatterning. The micropatterning was performed on a Nikon Eclipse Ti2 Microscope with a 20x S Plan Fluor ELWD Objective (NA = 0.45). The passivated surface coated with the photoactivator PLPP was placed on the microscope stage. The photo-micropatterning was controlled with the Leonardo software and executed by the PRIMO optical module (Alvéole Lab) using the stitching mode and a 375 nm laser at a dose of 30 mJ/mm^2^. The patterned surface was then washed six times with deionized water and stored in PBS at 4 °C until further use.

For cell seeding, the patterned surface was coated with 2.5% human collagen (Sigma #C5533-5MG) diluted in water for 1 h at RT. An excess of T84 pMx1-mCherry or T84 pMx1-eGFP cells were then seeded and incubated for 2 h at 37 °C. Precisely, 1,000,000 cells in 2 mL culturing media were used for the 25 mm diameter coverslips (in a 6-well cell culture plate Greiner Bio-One #657160) and 200,000 cells in 200 µL culturing media were added to the apical compartment of transwell inserts (the basolateral compartment of transwells was filled with 600 µl culturing media). Non-adherent cells were then washed away 2 h post seeding with two times PBS, and then fresh culturing medium was added. One day post-seeding, a medium change was performed, and on the second day post-seeding cells were treated with IFNs.

For cells seeded on micropatterned 25 mm diameter coverslips: Immediately after treating, live cell imaging was performed using a ZEISS Celldiscoverer 7 Widefield microscope using a 20 ×1 magnification (NA = 0.8) at 37 °C and 5% CO_2_ for 24 h, taking an image every 12 h starting at 0 h post treatment. For cells seeded on micropatterned transwell: Cells were fixed in 2% PFA at 0, 12, and 24 h post treatment and mounted with DAPI (Invitrogen, #P36935). Imaging was performed using the ZEISS Celldiscoverer 7 Widefield microscope using a 20 ×1 magnification (NA = 0.8).

To analyze the spatial heterogeneity of immune response in the radial direction, CellProfiler 3.1.9 was used to segment each population in 14 µm-radius rings (6 rings in total and the center area), and then measure the prom-Mx1-eGFP mean fluorescence intensity (MFI) within each ring. The MFI was normalized to the mock-treatment of the respective ring. To analyze the spatial heterogeneity of IFN-dependent immune response at the edge and the center, CellProfiler 3.1.9 was used to generate masks that divide each population into an edge (28 µm ring) and a center, and to measure the prom-Mx1-eGFP fluorescence intensity within the edge or the center of the population. The fluorescence intensity was normalized to the mock-treatment of the respective time-point and population region (edge or center).

### Surface biotinylation and surface proteome analysis

T84 cells were cultured in Corning transwell inserts (1.5 × 10^5^ cells/insert) in 1% O_2_ atmosphere until fully polarized (typically 2–4 days). Cells were then washed thrice in PBS and treated with 1 mg/mL Sulfo NHS-SS-Biotin (ThermoFisher) in biotinylation buffer (10 mM HEPES, 130 mM NaCl, 2 mM MgSO_4_, 1 mM CaCl_2_, pH 7.9) on the apical or basolateral side for 15 min on ice (biotinylation buffer was added to the opposite side to prevent drying). After incubation, cells were washed with 100 mM glycine for three times (last wash was left on cells for 10 min) to remove and quench excess biotin. Membranes were then cut and added to cell lysis buffer (50 mM HEPES, 150 mM NaCl, 5 mM EDTA, 1% Triton x-100, 0.1% SDS, pH 7.4) for 30 min in ice. After brief sonication and sedimentation of insoluble fragments, the protein amount was quantified using the DC protein assay kit (Biorad) and the same amount of total lysate for each sample (apical, basolateral and no biotinylation sample (background)) was loaded with High Capacity Neutravidin Agarose beads (ThermoFisher) and incubated overnight at 4 °C on an orbital shaker. The day after, supernatant was removed and beads were washed twice with high salt buffer (1 M NaCl, 50 mM HEPES, 0.1% Triton x-100, pH 7.4) and twice in 50 mM HEPES (pH 7.4). Beads were then incubated in Laemmli buffer for 20 min at RT on an orbital shaker. The supernatant containing the biotinylated surface proteins was then harvested, and loaded and ran on an SDS-PAGE for purification. Bands were excised and digested wit trypsin using a standard protocol (Shevchenko et al, 1996). After digestion, peptides were extracted and dried for LC-MS analysis. Peptides were reconstituted in 15 µL of 0.05% trifluoroacetic acid, 4% acetonitrile, and 6.6 µL were analyzed by an Ultimate 3000 reversed-phase capillary nano liquid chromatography system connected to a Q Exactive HF mass spectrometer (Thermo Fisher Scientific). Samples were injected and concentrated on a trap column (PepMap100 C18, 3 µm, 100 Å, 75 µm i.d. × 2 cm, Thermo Fisher Scientific) equilibrated with 0.05% trifluoroacetic acid in water. LC separations were performed on a capillary column (Acclaim PepMap100 C18, 2 µm, 100 Å, 75 µm i.d. × 25 cm, Thermo Fisher Scientific) at an eluent flow rate of 300 nl/min. Mobile phase A contained 0.1% formic acid in water, and mobile phase B contained 0.1% formic acid in 80% acetonitrile / 20% water. The column was pre-equilibrated with 5% mobile phase B followed by an increase of 5–44% mobile phase B in 100 min. Mass spectra were acquired in a data-dependent mode utilizing a single MS survey scan (*m/z* 350–1650) with a resolution of 60,000 and MS/MS scans of the 15 most intense precursor ions with a resolution of 15,000. The dynamic exclusion time was set to 20 s and automatic gain control was set to 3 × 10^6^ and 1 × 10^5^ for MS and MS/MS scans, respectively.

MS and MS/MS raw data were analyzed using the MaxQuant software package (version 1.6.14.0) with implemented Andromeda peptide search engine (Tyanova et al, 2016). Data were searched against the human reference proteome downloaded from Uniprot (75,074 sequences, taxonomy 9606, last modified March 10, 2020) using the default parameters except for the following changes: label-free quantification (LFQ) enabled, match between runs enabled, iBAQ enabled, max missed cleavages: 3.

Perseus downstream analysis was performed as follows: Proteins were cross referenced with the UniProt human database for gene ontology terms (Plasma membrane, plasma membrane part, cell surface, cell outer membrane), then filtered out if they had less than 3 replicates or if they had no GO term matching the above-mentioned search. Background samples were used to filter out any protein nonspecifically bound to the Neutravidin beads. Significantly enriched proteins on the apical or basolateral side were assigned based on their log(2) LFQ signal between apical- and basolateral-biotinylated samples, using pairwise *t*-tests coupled with sample randomization with false discovery rate (FDR) = 0.05.

### FITC-dextran permeability assay

T84 cells were grown on transwell inserts as a monolayer, with 600 µL media in the basolateral compartment and 200 µL media in the apical compartment. Media was removed from the apical compartment of the transwell and replaced by 200 µL of fresh medium containing of 2 mg/mL fluorescein isothiocyanate (FITC)-labeled dextran (4 kDa) (Sigma-Aldrich, # 46944-500MG-F). As a negative control and to calculate the background, culture media alone was used on a well without cells. For the positive control (maximum diffusion of FITC-Dextran from apical to basolateral compartment) 200 µL of 2 mg/mL of FITC-Dextran was added to the apical side of a well without cells and 600 µL culturing media were added to the basolateral compartment. Cells and controls were incubated for 3 h at 37 °C and then media was collected from the basolateral compartment. Fluorescent signal was measured using an 800TS Microplate Reader (BioTek) at an excitation wavelength of 495 nm. A standard curve by serial dilution of the FITC-Dextran in culturing media was done to assess the basolateral FITC-Dextran concentration.

### IFN diffusion assay and detection of IFNs by HEK-blue assay

T84 WT and ZO1-KO cells were grown on transwell inserts as a monolayer, with 600 µL media in the basolateral compartment and 200 µL media in the apical compartment. Media was removed from the apical compartment of the transwell and treated with 200 µL of fresh medium containing no IFNs (mock treatment), or either 2000 IU/mL IFNβ1 or 300 ng/mL IFNλ1-3. Cells were incubated for 3 h at 37 °C and then media was collected from the basolateral compartment.

To measure diffusion of IFNs from the apical to the basolateral transwell compartment, the HEK-blue assay was performed. Shortly, the HEK-blue™ IFN-α/β cells (Invivogen #hkb-ifnab) and HEK-Blue™ IFN-λ cells (Invivogen #hkb-ifnl) were used as IFN reporter cell lines. Importantly, since HEK-blue™ IFN-α/β cells are also able to respond to IFN-λ, previously in our laboratory cells were transfected with a CRISPR K.O vector targeting the IFNLR1, generating a reporter cell line that can only sense type I IFNs (described in (Metz-Zumaran et al, 2022b)). HEK-blue IFN reporter cells were seeded in FBS-inactivated DMEM medium at a density of 30,000 cells per well in 96-well plates 1 day before the experiment. Next, 50-μL portions of media from the basolateral transwell compartment were added to HEK-Blue cells for 24 h, and the levels of secreted embryonic alkaline phosphatase was measured using QUANTI-Blue (InvivoGen, catalog no. rep-qbs). Fluorescent signal was measured using an 800TS Microplate Reader (BioTek) at an excitation wavelength of 800 nm. A standard curve by serial dilution of both type of IFNs in culturing media was done to assess the basolateral IFN concentration.

### Viral infections

All virus infections were performed with a multiplicity of infection (MOI) of 1 as determined in T84 cells. Cells were mock treated or pre-treated with type I and type III IFNs for 24 h. Pre-treatment culture medium was replaced with fresh medium containing MRV or VV at an MOI of 1 and supplemented with the same interferons as the pre-treatment. 16 h post-infection, cells were fixed in 2% PFA for immunofluorescent staining.

### Indirect immunofluorescence assay

Cells were seeded on glass coverslips for ZO1 staining and on plastic bottom multiwells for viral protein staining. Cells were fixed in 2% paraformaldehyde (PFA) for 20 min at RT. Cells were washed in 1X PBS and permeabilized in 0.5% Triton-X100 diluted in PBS for 15 min at RT. Cells were blocked using 3% BSA in PBS for 30 min at RT. Antibodies against ZO1 (Thermo Fisher Scientific #40-2200), Non-Structural Mammalian Reovirus Protein µNS (Shah et al, 2017), Cytochrome C (BD Biosciences #556432), TGN46 (Sigma Aldrich # T7576) and ISG15 (Santa Cruz Biotechnology #166755) were diluted in 1% BSA (in PBS) and incubated for 1 h at RT. Cells were washed with PBS three times, and incubated with DAPI (BD Biosciences, #564907) and secondary antibody conjugated to AF488 (Molecular Probes) diluted 1:1000 in 1% BSA (in PBS) for 30 min at RT. For cells infected with Vaccinia virus, immunostaining was omitted since the virus strains expresses eGFP and only the DAPI staining was done for visualization of cell nuclei. Cells were washed in 1X PBS three times. Cells seeded on coverslips were mounted, and cells seeded in multiwells were maintained in PBS until imaging. Cells were imaged on a ZEISS Celldiscoverer 7 Widefield microscope using a 20 ×0.5 magnification (Numerical Aperture NA = 0.5) or a confocal spinning disc Nikon Ti Andor microscope using the CFI Pl Apo 40× objective with a N.A. = 0.95.

### Organoid seeding in 2D

To assess the population context influence on IFN signaling in primary epithelial cells, 3D organoids were trypsinized and seeded in basal media into 8-well IBIDI chamber slides coated with 0.04 mg/mL human collagen (Sigma Aldrich #C5533). One day post seeding, medium was exchanged to differentiation medium (Table 1). When organoids flattened down into monolayers (typically on day 3/4), the cells were treated with IFNβ1 or IFNλ1-3 for 24 h before fixation and immunofluorescence staining as described above.

To compare basolateral and apical IFN treatment, organoids were seeded in basal media on transwell inserts coated with 10% matrigel (Corning #354230). To obtain dense monolayers on the transwell membranes, 3–5 well of 3D organoids were pooled per insert and after seeding, partial media changes were performed every two days. Depending on cell density, on day 4 or 6 post seeding air liquid interface (ALI) culturing was started by removing the media from the insert to induce organoid cell differentiation (Wang et al, 2019). TEER measurements were performed and upon a TEER of ≥450–1000 Ω/cm^2^, typically around 11 days post-seeding, organoids were treated with IFNβ1 or IFNλ1-3 either from the apical or the basolateral side for 24 h as described before.

### 3D basolateral-out and apical-out organoids

Traditionally cultured 3D organoids have a “basolateral-out” (BL-out) conformation, enabling IFN treatment of the basolateral membrane. To this end, organoids were trypsinized and cultured in 100% Matrigel in basal media before changing to differentiation media. To treat the apical membrane of 3D organoids, we employed a recently published protocol to induce polarity reversal of 3D organoids resulting in “apical-out” (A-out) organoids (Co et al, 2021). Briefly, when ileum organoids were ready to be split, they were dissociated in 5 mM EDTA for 1 h at 4 °C and then seeded into differentiation media in suspension into ultra-low-attachment plates (Corning). BL-out or A-out organoids were treated with IFNβ1 or IFNλ1-3 5 d post seeding either by adding IFN-containing differentiation media onto the Matrigel (BL-out organoids) or by treating in suspension (A-out organoids). 24 h post treatment, organoids were washed in cold PBS and then fixed in 2% paraformaldehyde for 30 min at room temperature for immunofluorescent stainings or lysed for RT-q-PCR as previously described. To confirm the correct orientation (BL-out vs. A-out), organoids were stained using Phalloidin-AF647 and DAPI in 0.1% saponin and 2% FBS in PBS for 2 h at room temperature and imaging was performed using a ZEISS Celldiscoverer 7 microscope. RNA extraction, cDNA synthesis, and RT-q-PCR to explore ISG induction were performed as described above.

### Statistics, computational analyses, and softwares

All statistical analyses were performed by statistical tests as specified in figure legends using the GraphPad Prism software package (Version 8.0.1).

To quantify the number of Vaccinia virus eGFP or MRV infected cells, ilastik 1.2.0 was used on DAPI images to generate a mask representing each nucleus as an individual object. These masks were used on CellProfiler 3.1.9 to measure the fluorescence intensity coming from the virus infection within each nucleus. A threshold was set based on the basal fluorescence of non-infected samples, and all nuclei with a higher fluorescence were counted as infected cells.

Illustrations were generated with Biorender.com and data was plotted using the GraphPad Prism software package (Version 8.0.1).

## Supplementary information

Dataset EV1

Source Data Fig. 1

Source Data Fig. 2

Source Data Fig. 3

Source Data Fig. 4

Source Data Fig. 6

Source Data Fig. 7

Peer Review File

Expanded View Figures

## Data Availability

The dataset produced in this study is available in the following database: Mass spectrometry proteomics data: ProteomeXchange via the PRIDE database PXD047936.
