# Supplementary figures and images for: The population context is a driver of the heterogeneous response of epithelial cells to interferons
Source: Mol Syst Biol. 2024 Jan 25;20(3):7. doi: 10.1038/s44320-024-00011-2 (PMC10912784; doi:10.1038/s44320-024-00011-2)

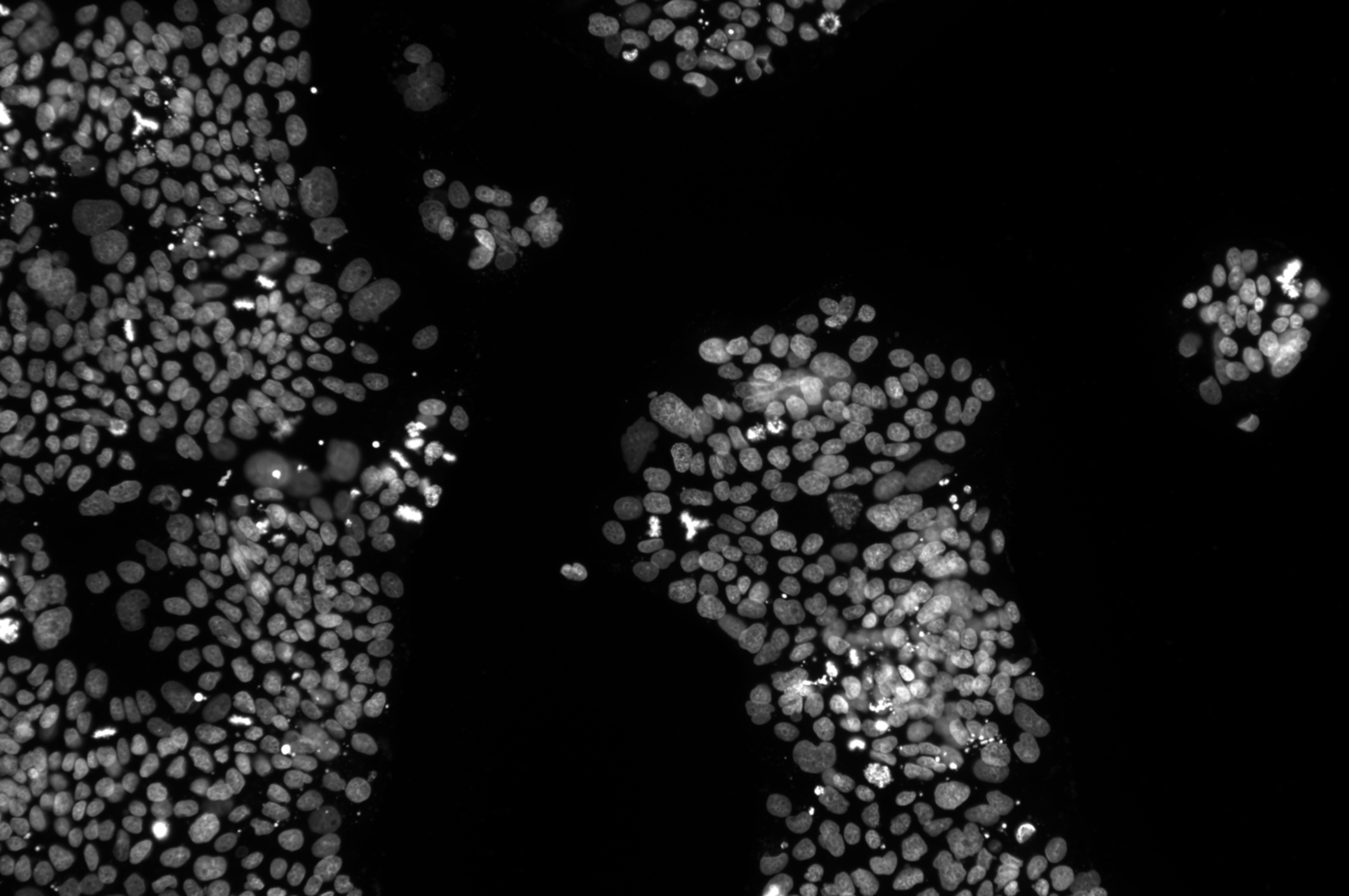

Supplement: Supplementary file 2 — Source Data Fig. 1 [file 44320_2024_11_MOESM2_ESM.zip › Figure 1/1A/IFNb1 2000 IUxml-1_merge DAPI pMx1-GFP.tif]

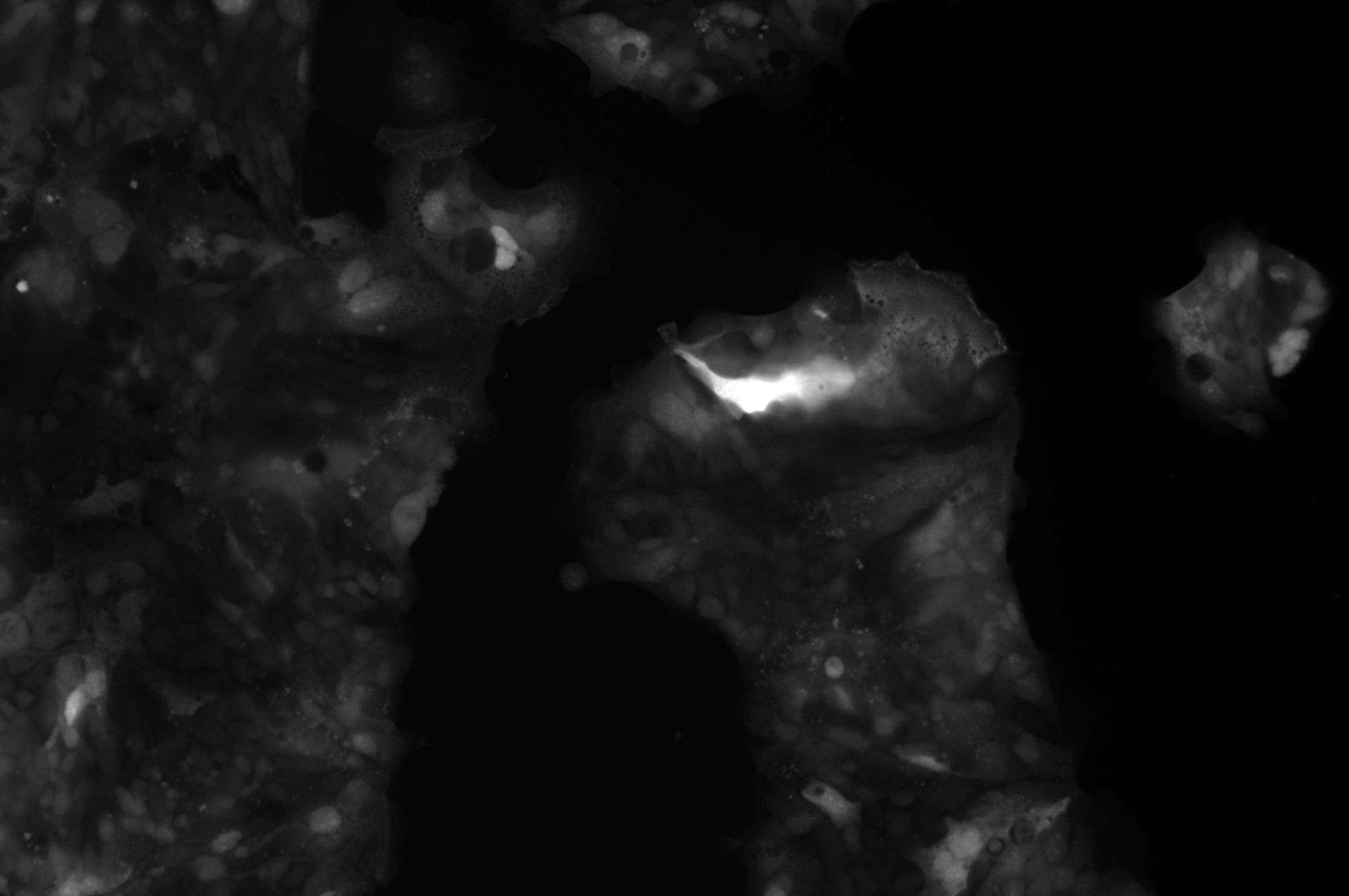

Supplement: Supplementary file 2 — Source Data Fig. 1 [file 44320_2024_11_MOESM2_ESM.zip › Figure 1/1A/IFNb1 2000 IUxml-1_pMx1-GFP.tif]

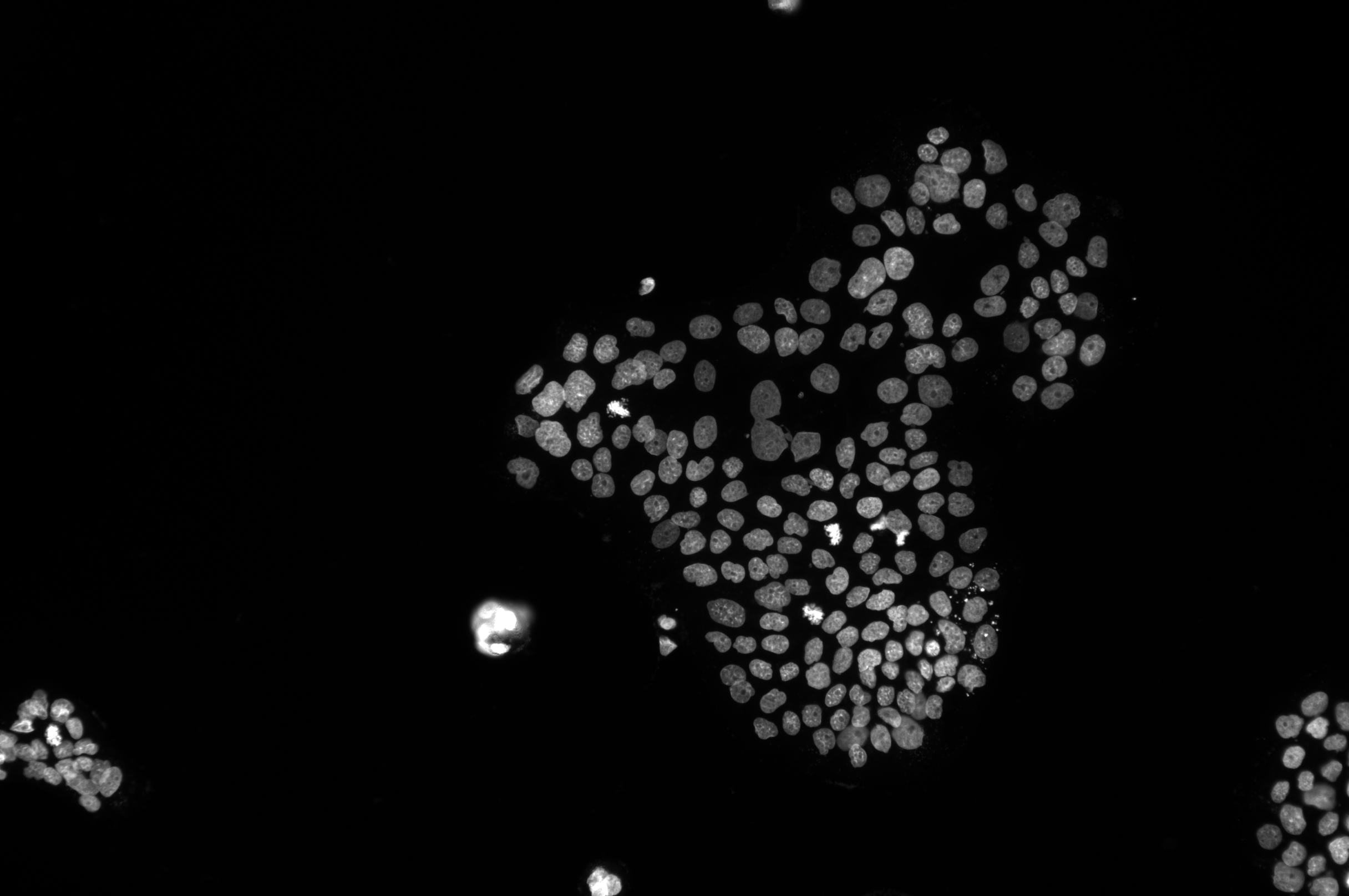

Supplement: Supplementary file 2 — Source Data Fig. 1 [file 44320_2024_11_MOESM2_ESM.zip › Figure 1/1A/IFNL1-3 300 ngxml-1_merge DAPI pMx1-GFP.tif]

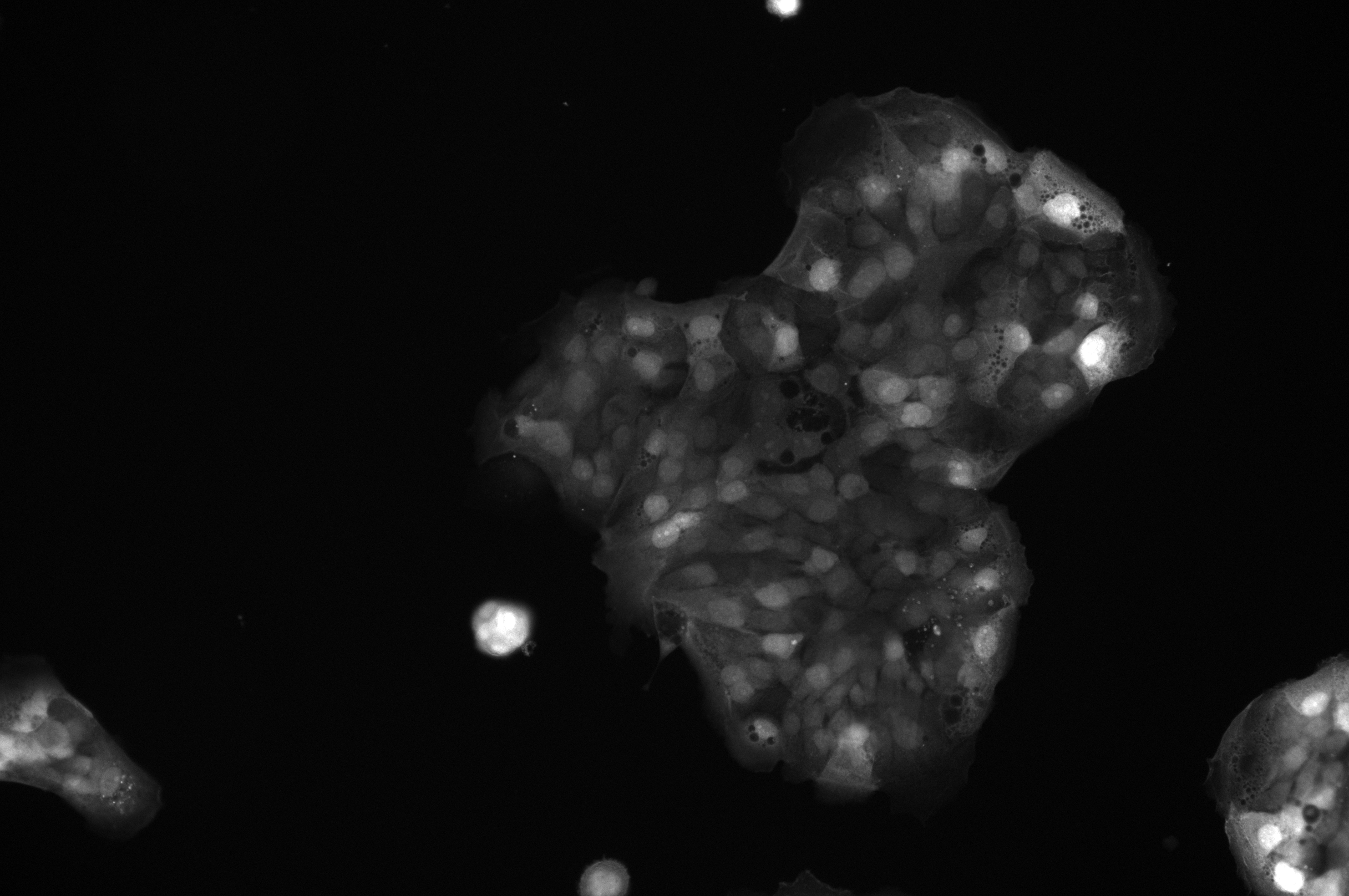

Supplement: Supplementary file 2 — Source Data Fig. 1 [file 44320_2024_11_MOESM2_ESM.zip › Figure 1/1A/IFNL1-3 300 ngxml-1_pMx1-GFP.tif]

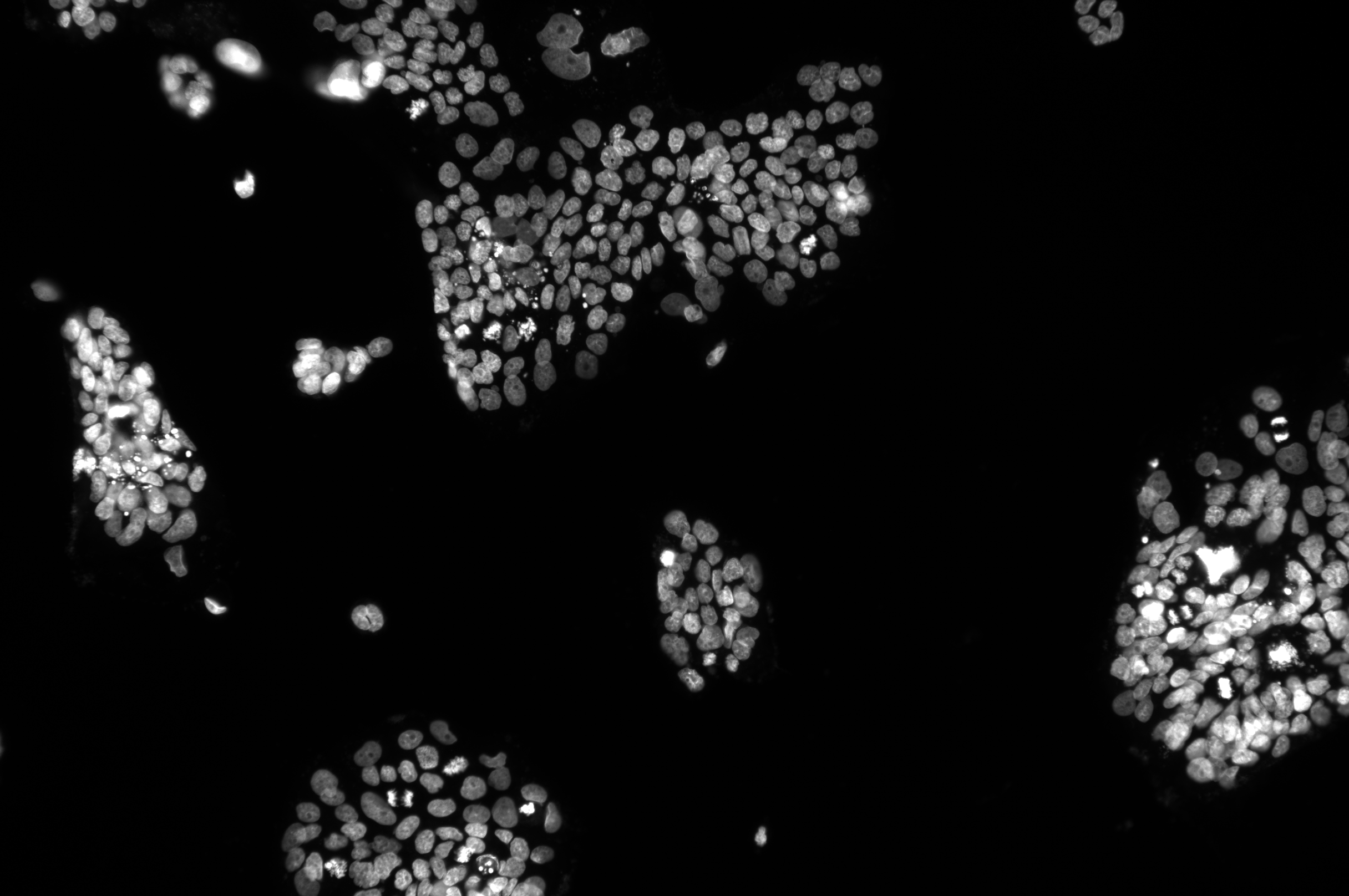

Supplement: Supplementary file 2 — Source Data Fig. 1 [file 44320_2024_11_MOESM2_ESM.zip › Figure 1/1A/mock_merge DAPI pMx1-GFP.tif]

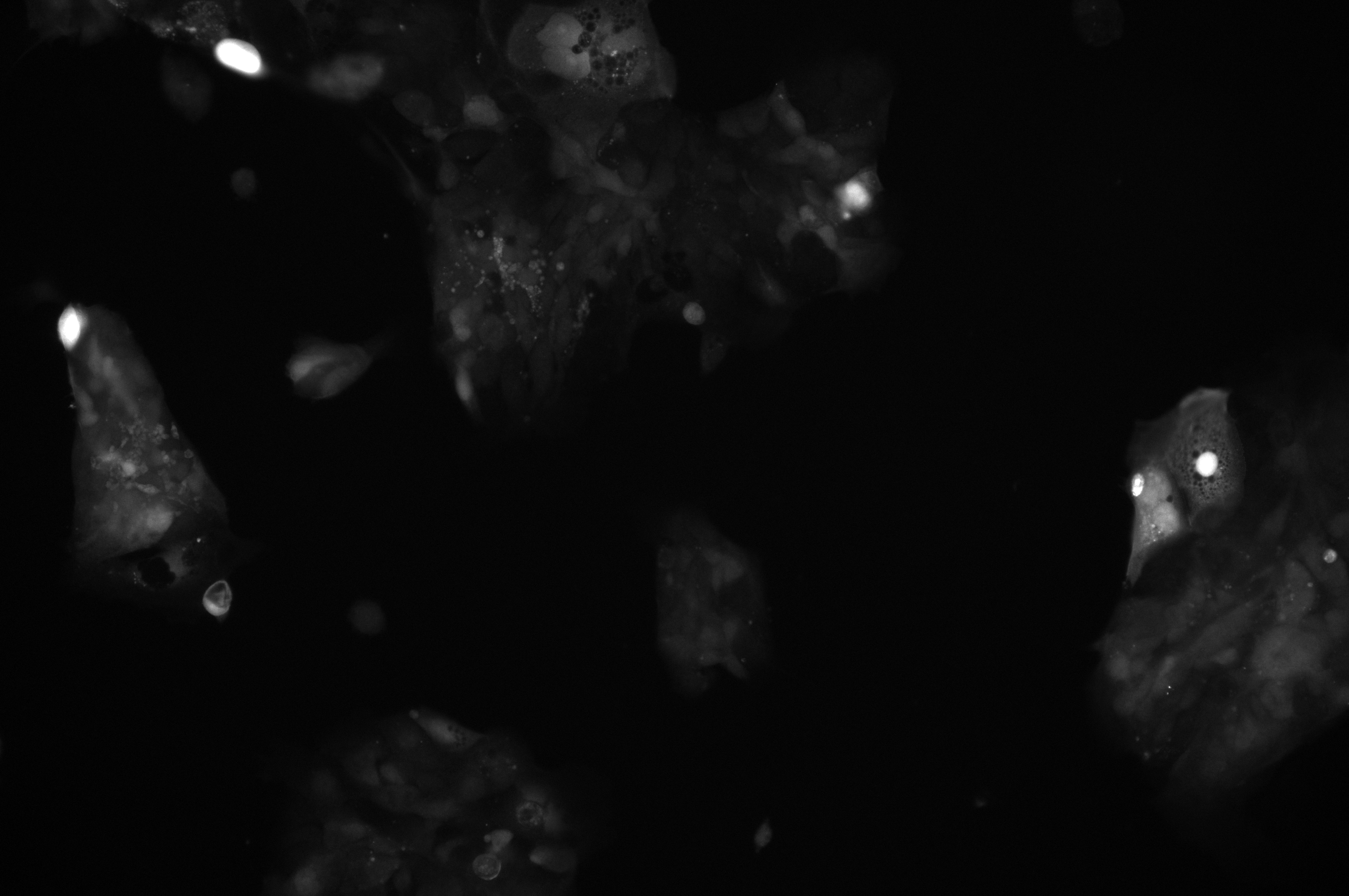

Supplement: Supplementary file 2 — Source Data Fig. 1 [file 44320_2024_11_MOESM2_ESM.zip › Figure 1/1A/mock_pMx1-GFP.tif]

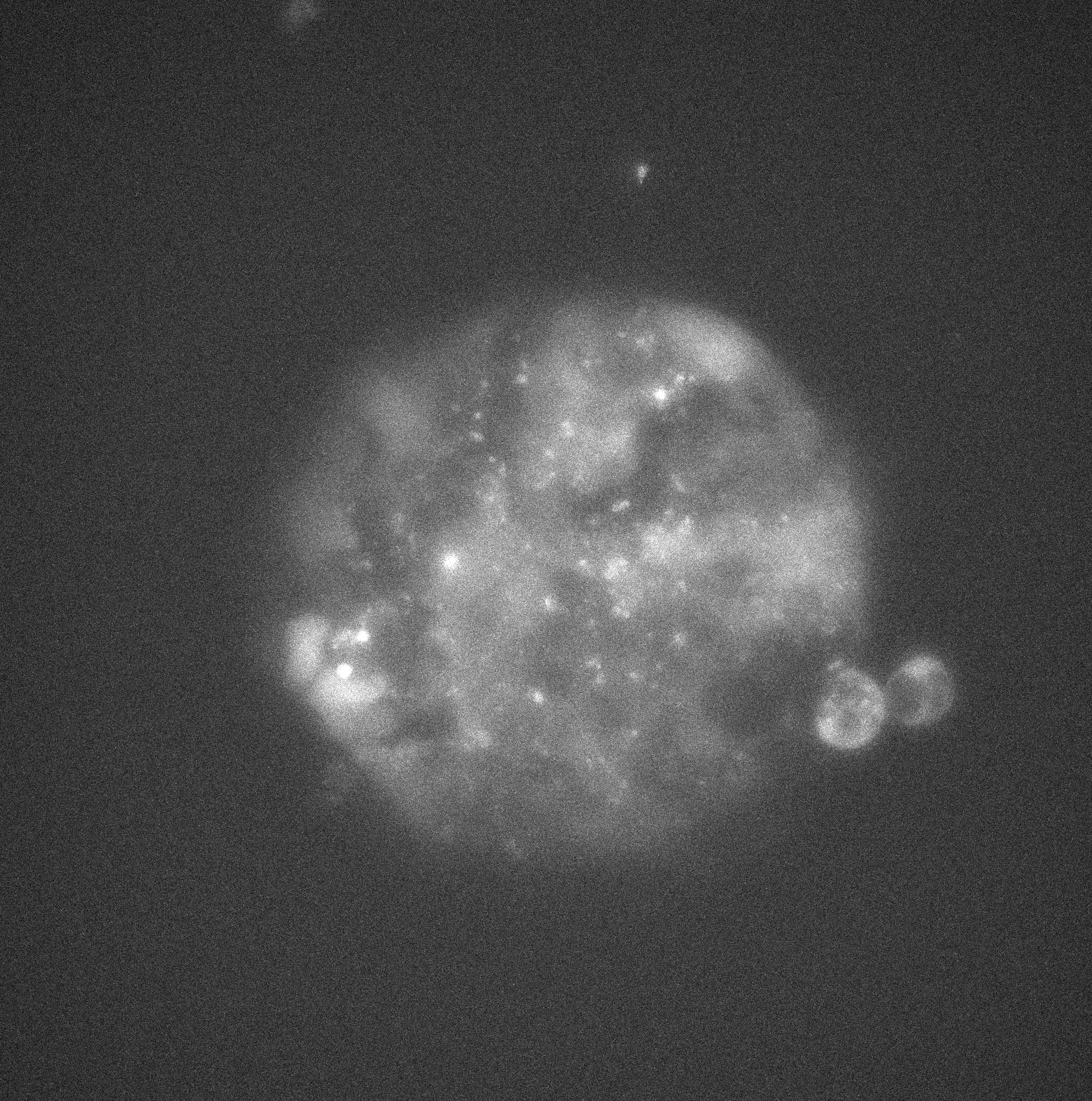

Supplement: Supplementary file 3 — Source Data Fig. 2 [file 44320_2024_11_MOESM3_ESM.zip › Figure 2/2B/IFNb1 treatment_0h_pMx1-GFP channel.tif]

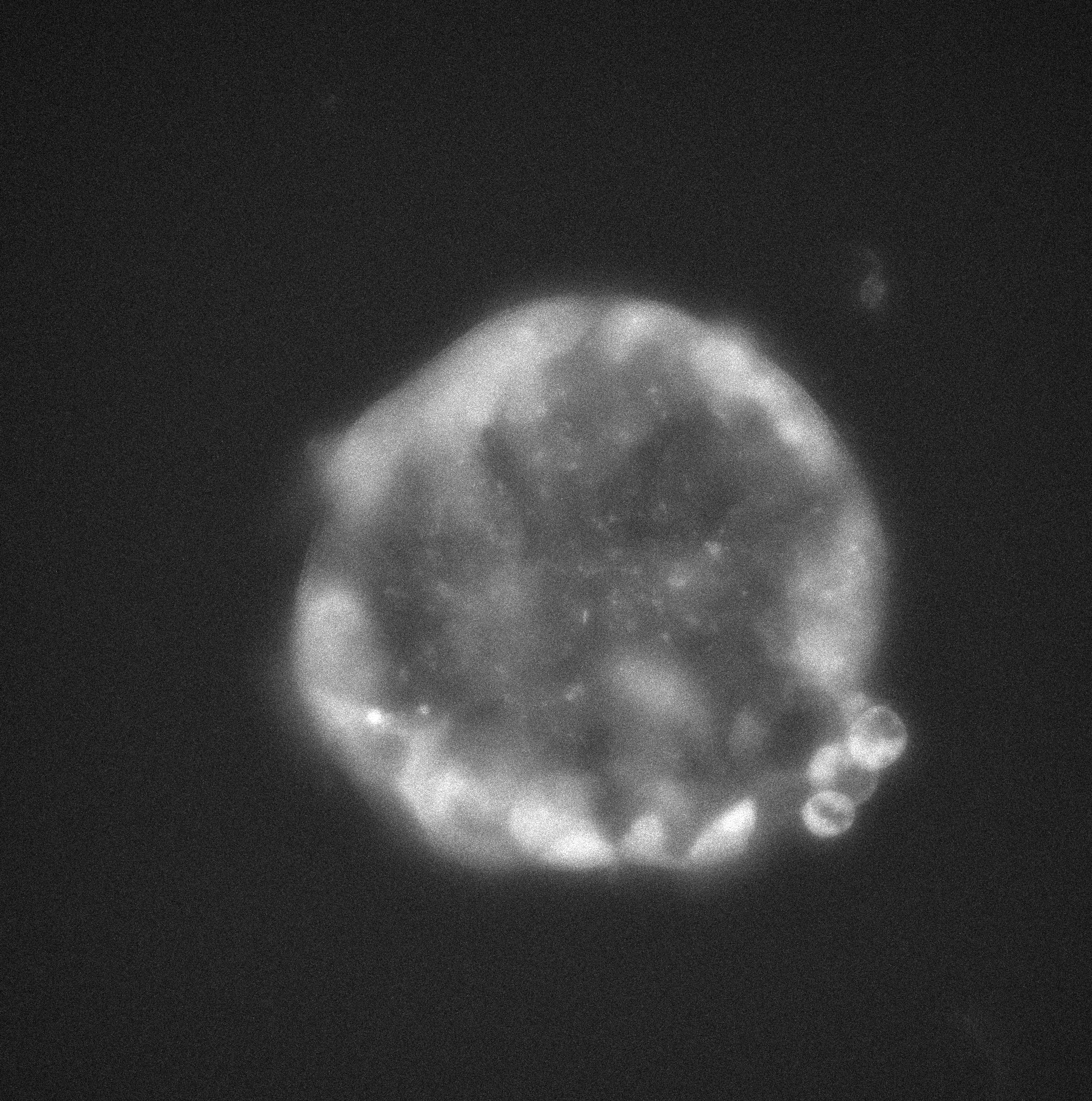

Supplement: Supplementary file 3 — Source Data Fig. 2 [file 44320_2024_11_MOESM3_ESM.zip › Figure 2/2B/IFNb1 treatment_12h_pMx1-GFP channel.tif]

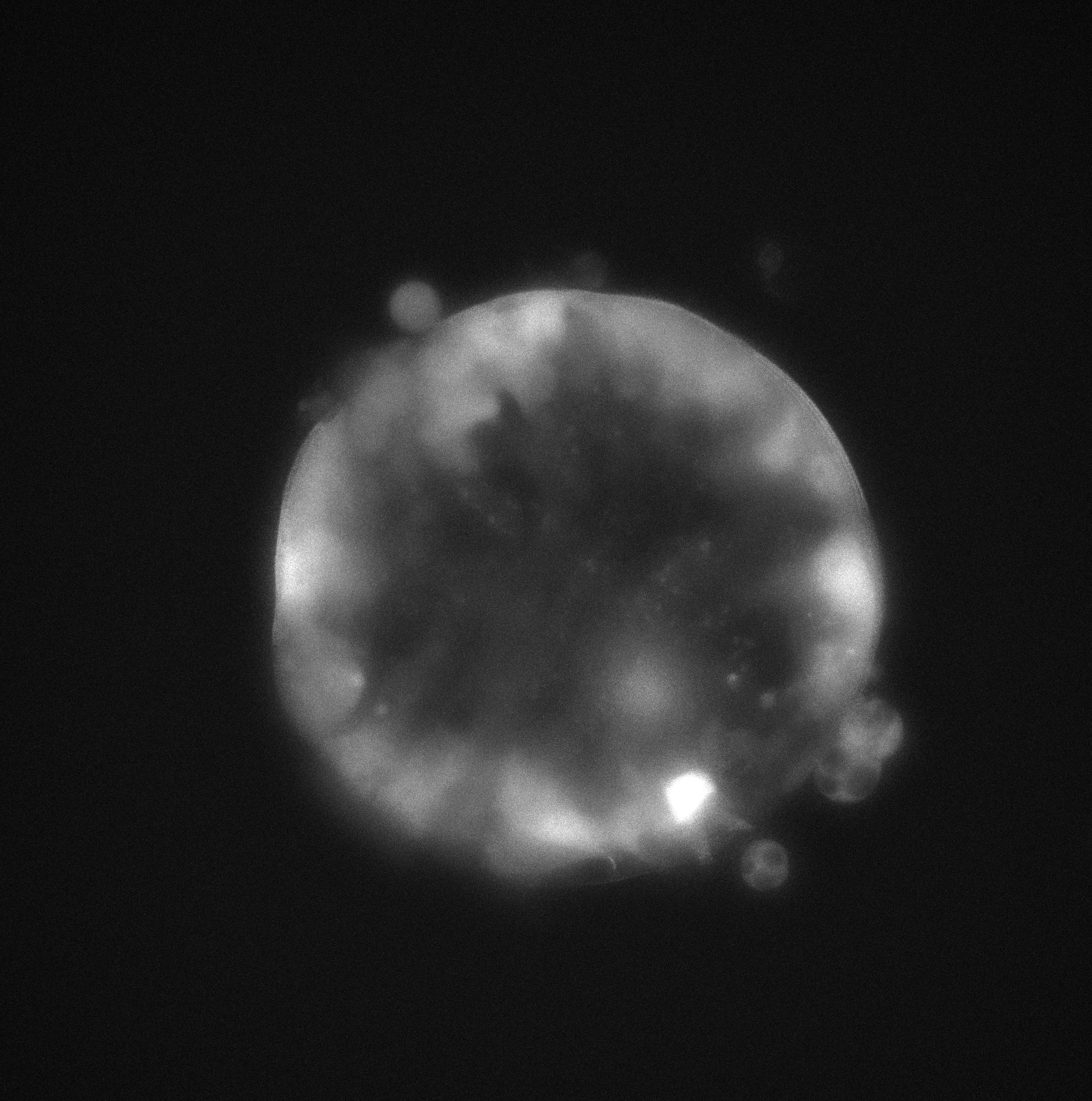

Supplement: Supplementary file 3 — Source Data Fig. 2 [file 44320_2024_11_MOESM3_ESM.zip › Figure 2/2B/IFNb1 treatment_24h_pMx1-GFP channel.tif]

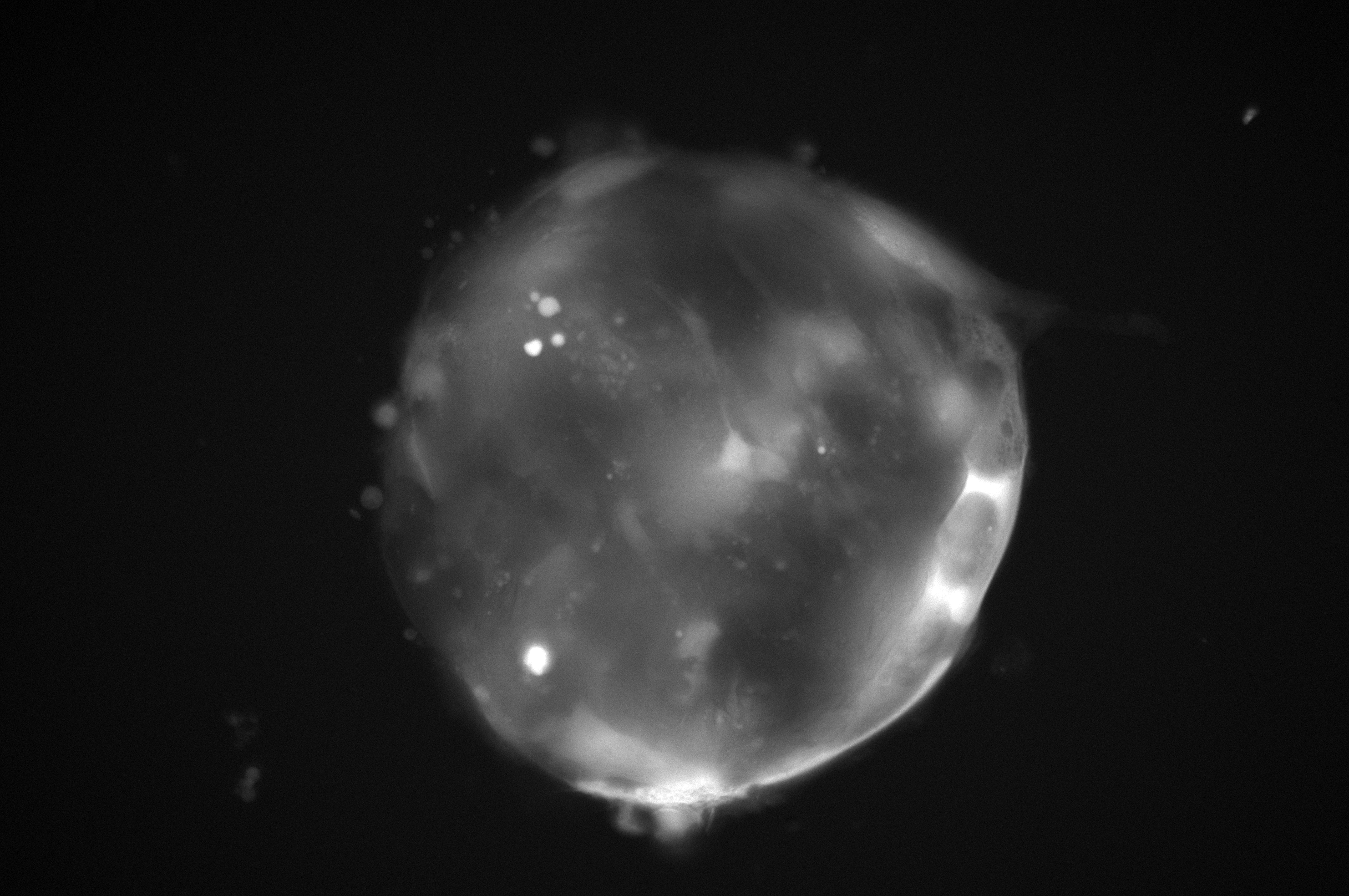

Supplement: Supplementary file 3 — Source Data Fig. 2 [file 44320_2024_11_MOESM3_ESM.zip › Figure 2/2B/IFNL1-3 treatment_0h_pMx1-GFP channel.tif]

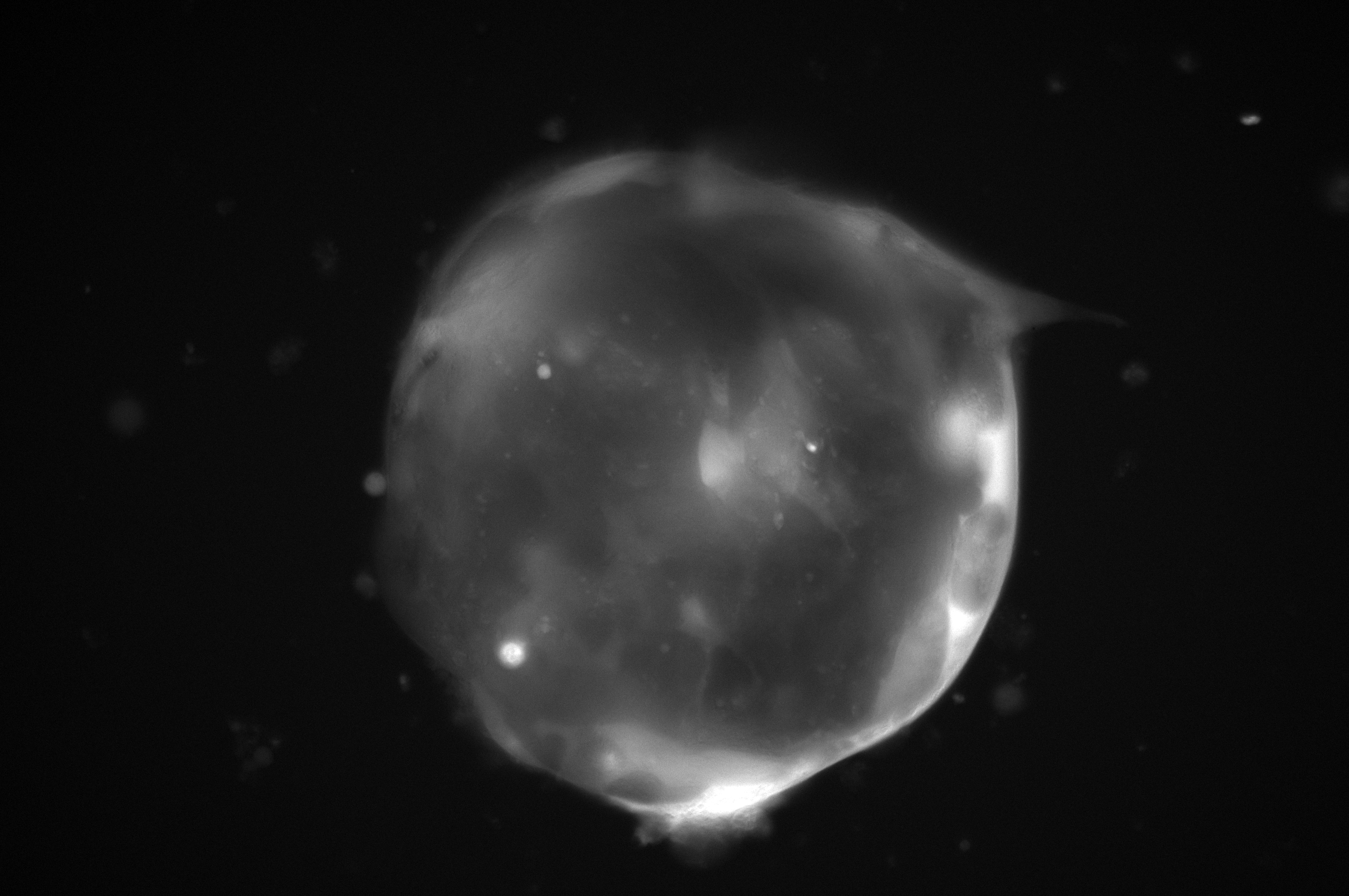

Supplement: Supplementary file 3 — Source Data Fig. 2 [file 44320_2024_11_MOESM3_ESM.zip › Figure 2/2B/IFNL1-3 treatment_12h_pMx1-GFP channel.tif]

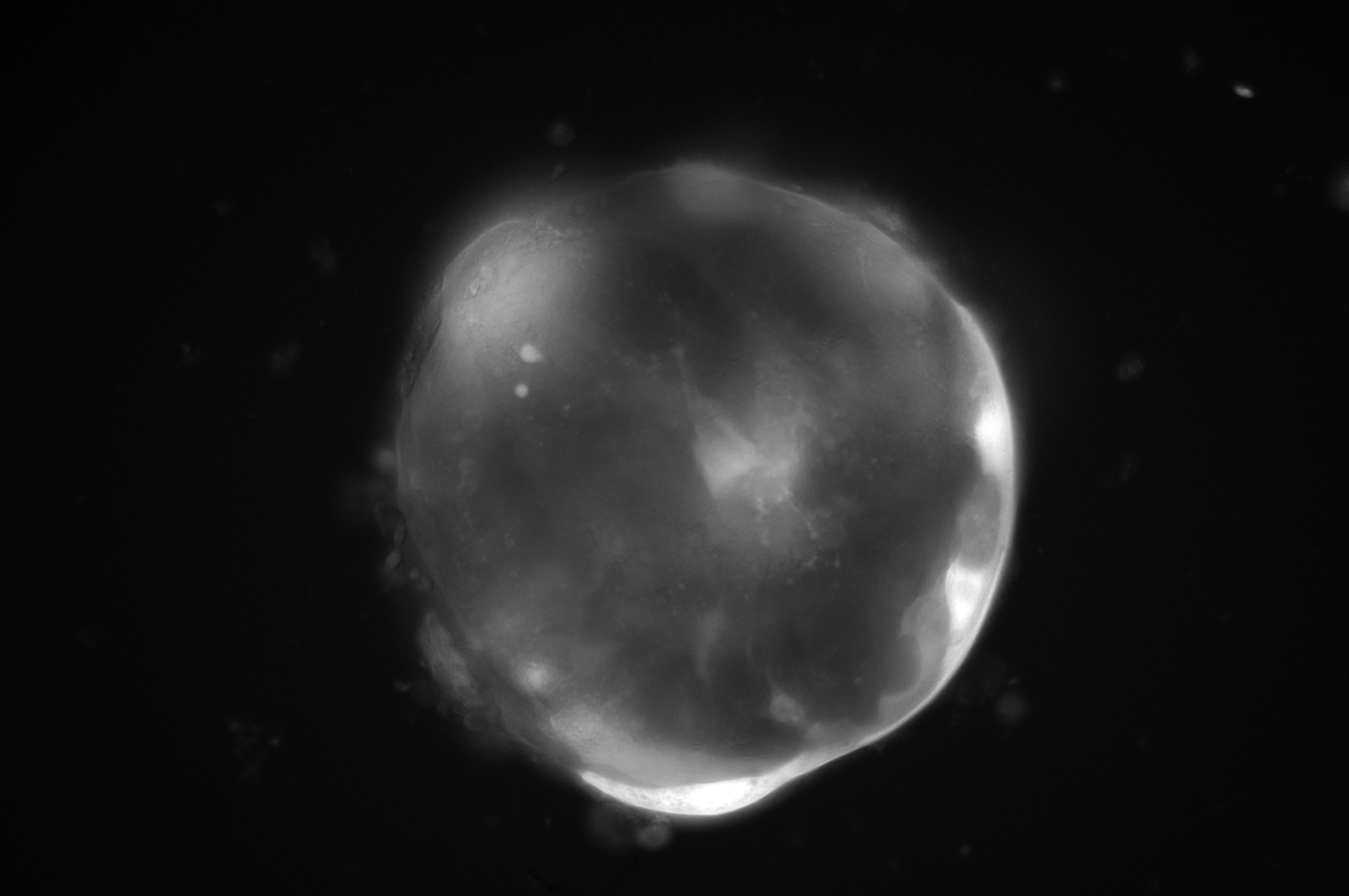

Supplement: Supplementary file 3 — Source Data Fig. 2 [file 44320_2024_11_MOESM3_ESM.zip › Figure 2/2B/IFNL1-3 treatment_24h_pMx1-GFP channel.tif]

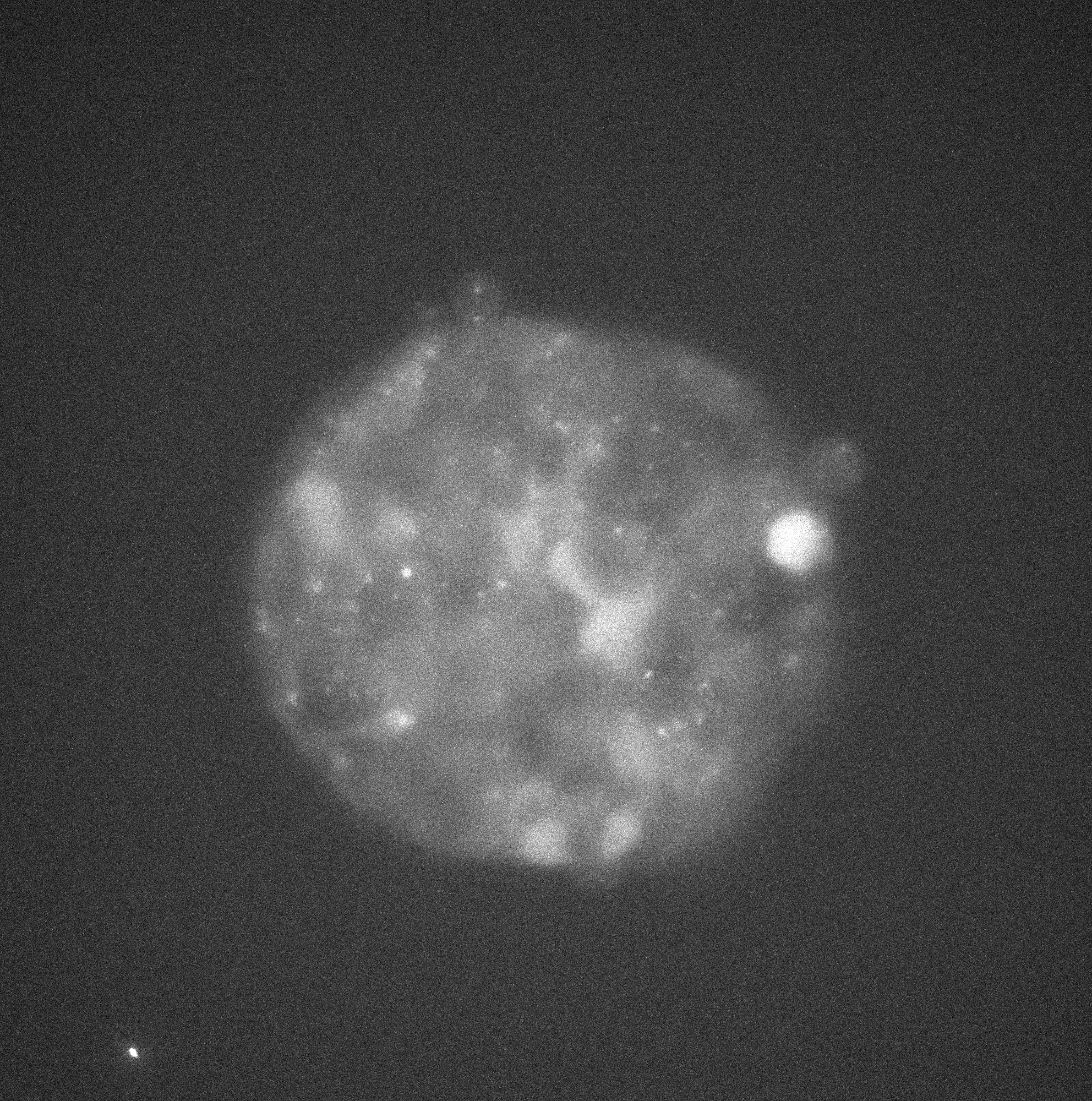

Supplement: Supplementary file 3 — Source Data Fig. 2 [file 44320_2024_11_MOESM3_ESM.zip › Figure 2/2B/mock treatment_0h_pMx1-GFP channel.tif]

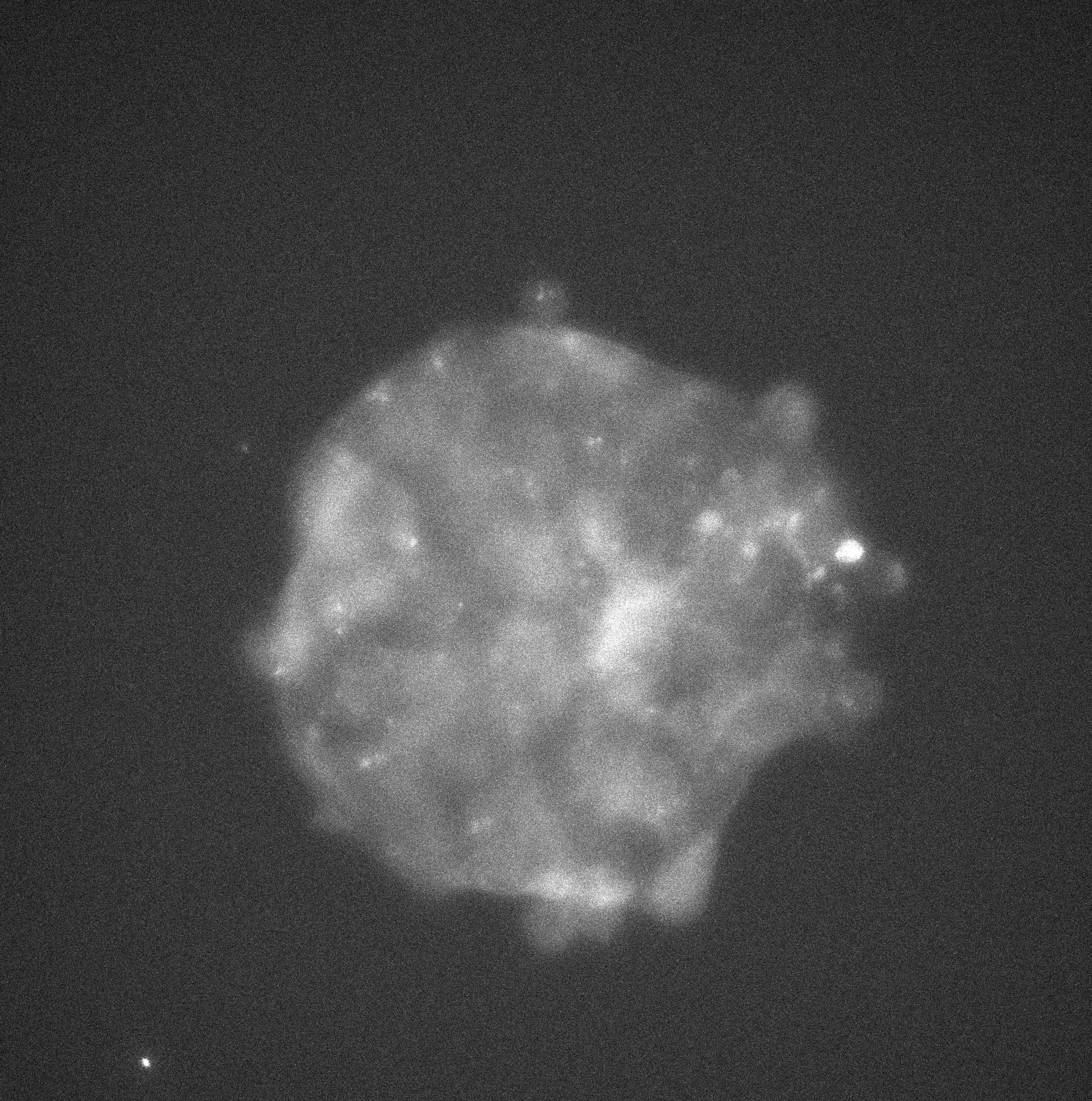

Supplement: Supplementary file 3 — Source Data Fig. 2 [file 44320_2024_11_MOESM3_ESM.zip › Figure 2/2B/mock treatment_12h_pMx1-GFP channel.tif]

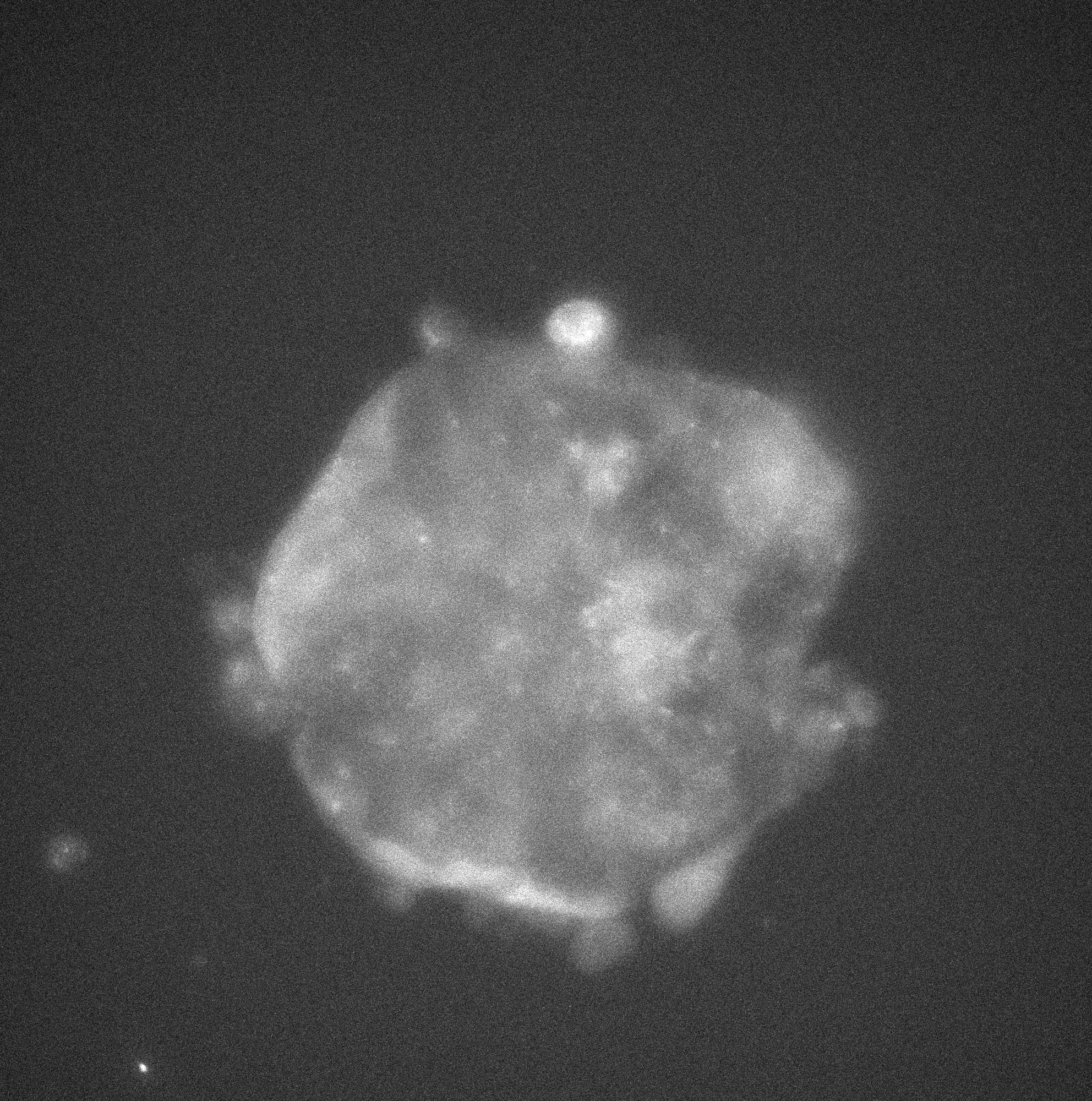

Supplement: Supplementary file 3 — Source Data Fig. 2 [file 44320_2024_11_MOESM3_ESM.zip › Figure 2/2B/mock treatment_24h_pMx1-GFP channel.tif]

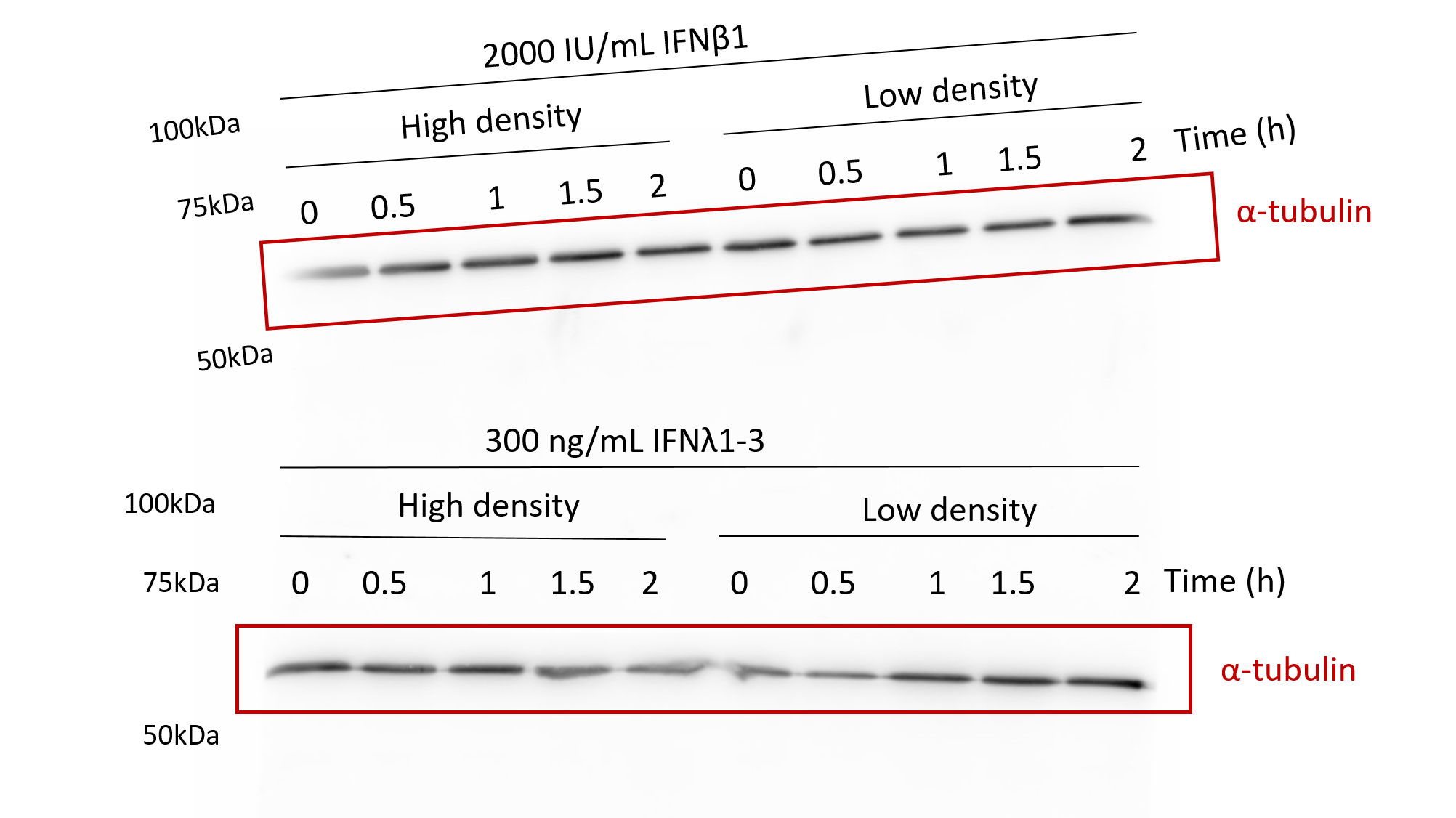

Supplement: Supplementary file 4 — Source Data Fig. 3 [file 44320_2024_11_MOESM4_ESM.zip › Figure 3/3C/a-tubulin.png]

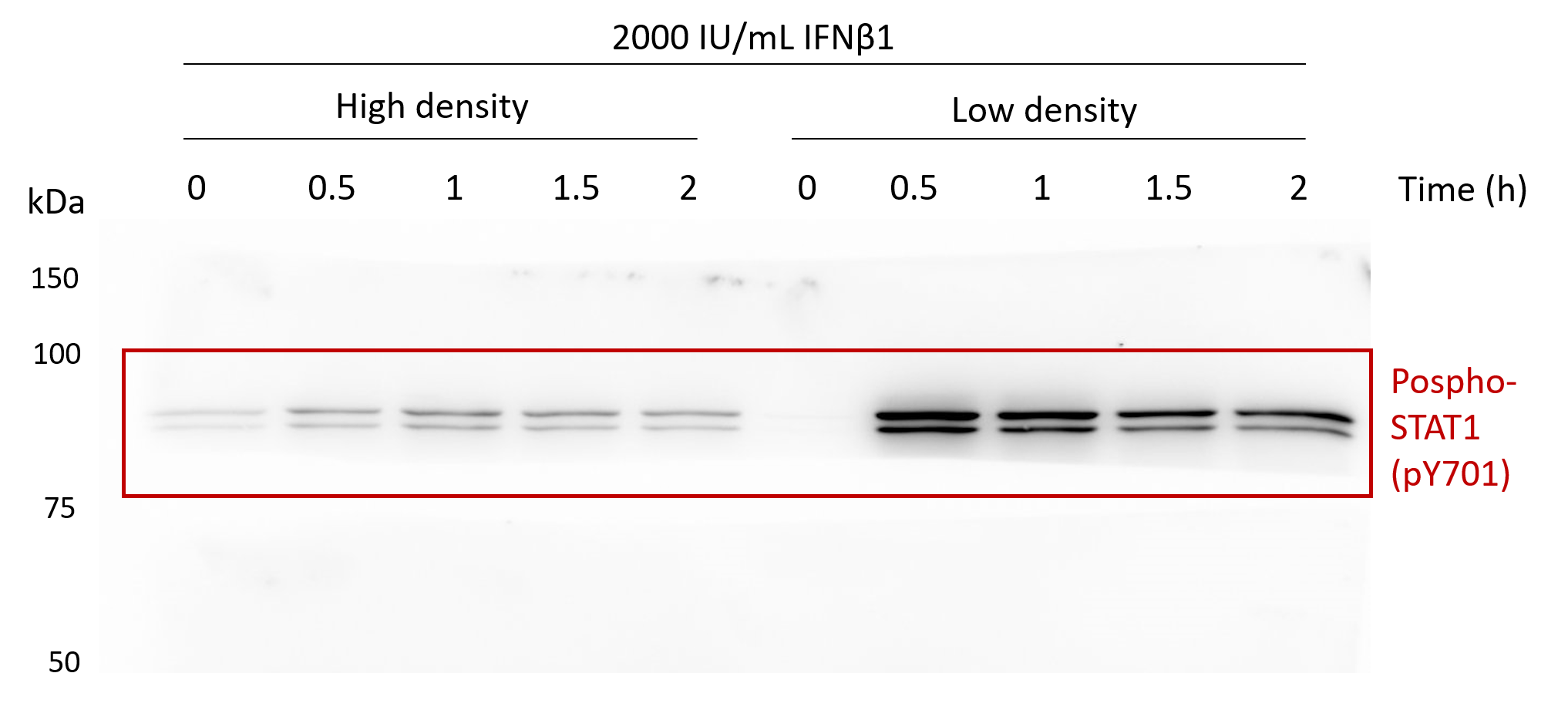

Supplement: Supplementary file 4 — Source Data Fig. 3 [file 44320_2024_11_MOESM4_ESM.zip › Figure 3/3C/IFNb1 treatment_pSTAT1.png]

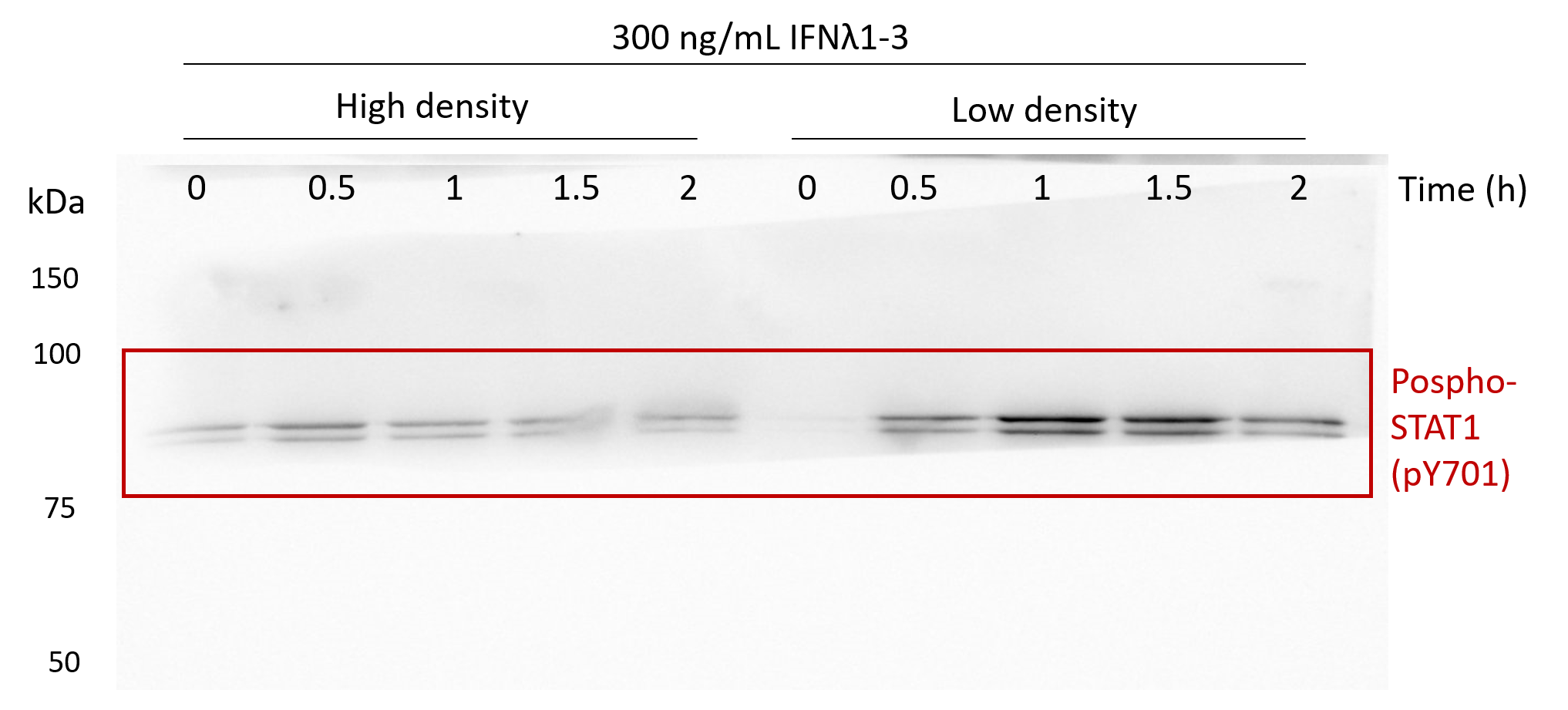

Supplement: Supplementary file 4 — Source Data Fig. 3 [file 44320_2024_11_MOESM4_ESM.zip › Figure 3/3C/IFNL1-3 treatment_pSTAT1.png]

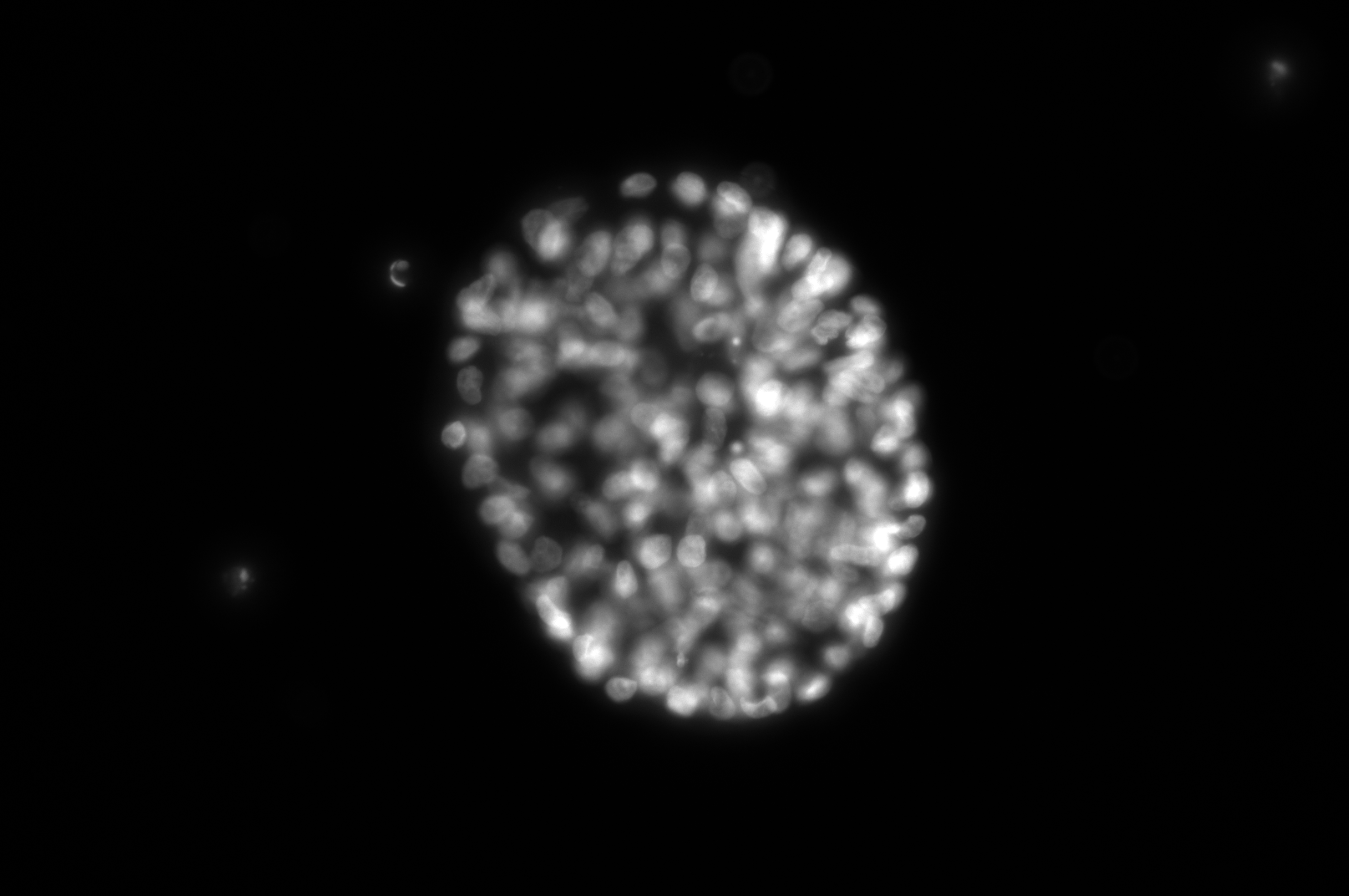

Supplement: Supplementary file 5 — Source Data Fig. 4 [file 44320_2024_11_MOESM5_ESM.zip › Figure 4/4F/Apical out_DAPI Phalloidin_1.tif]

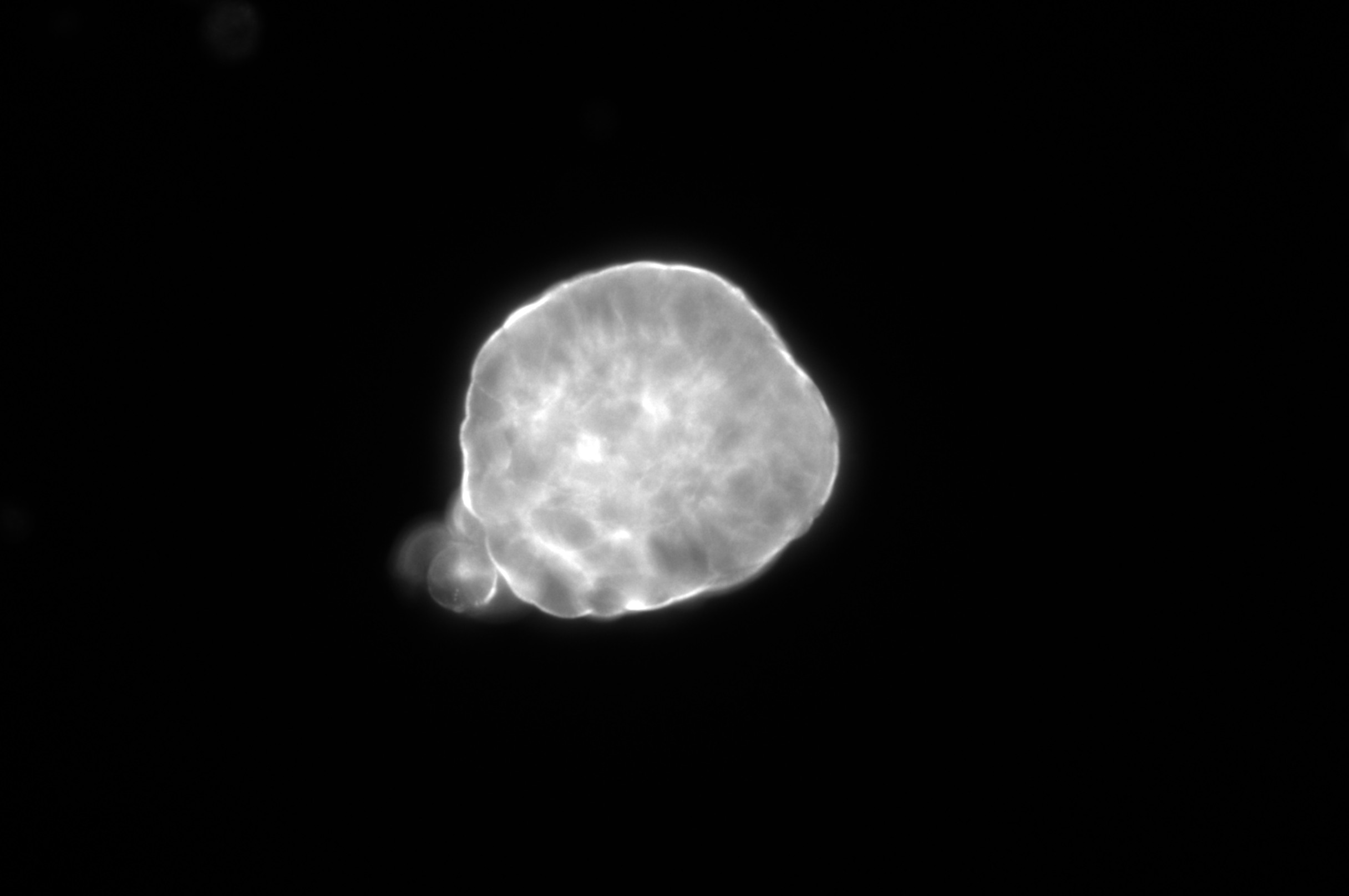

Supplement: Supplementary file 5 — Source Data Fig. 4 [file 44320_2024_11_MOESM5_ESM.zip › Figure 4/4F/Apical out_DAPI Phalloidin_2.tif]

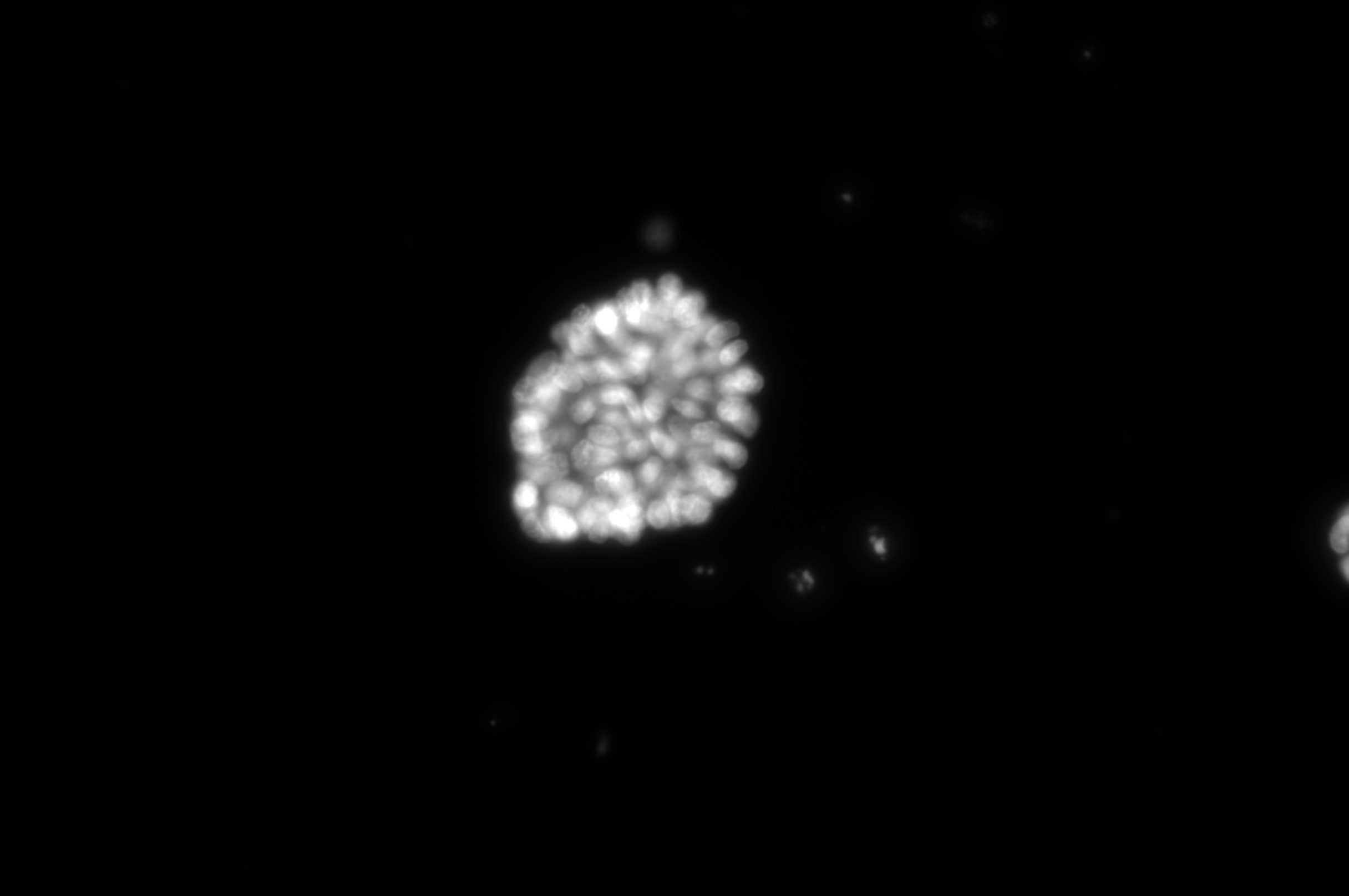

Supplement: Supplementary file 5 — Source Data Fig. 4 [file 44320_2024_11_MOESM5_ESM.zip › Figure 4/4F/Basolateral out_DAPI Phalloidin_1.tif]

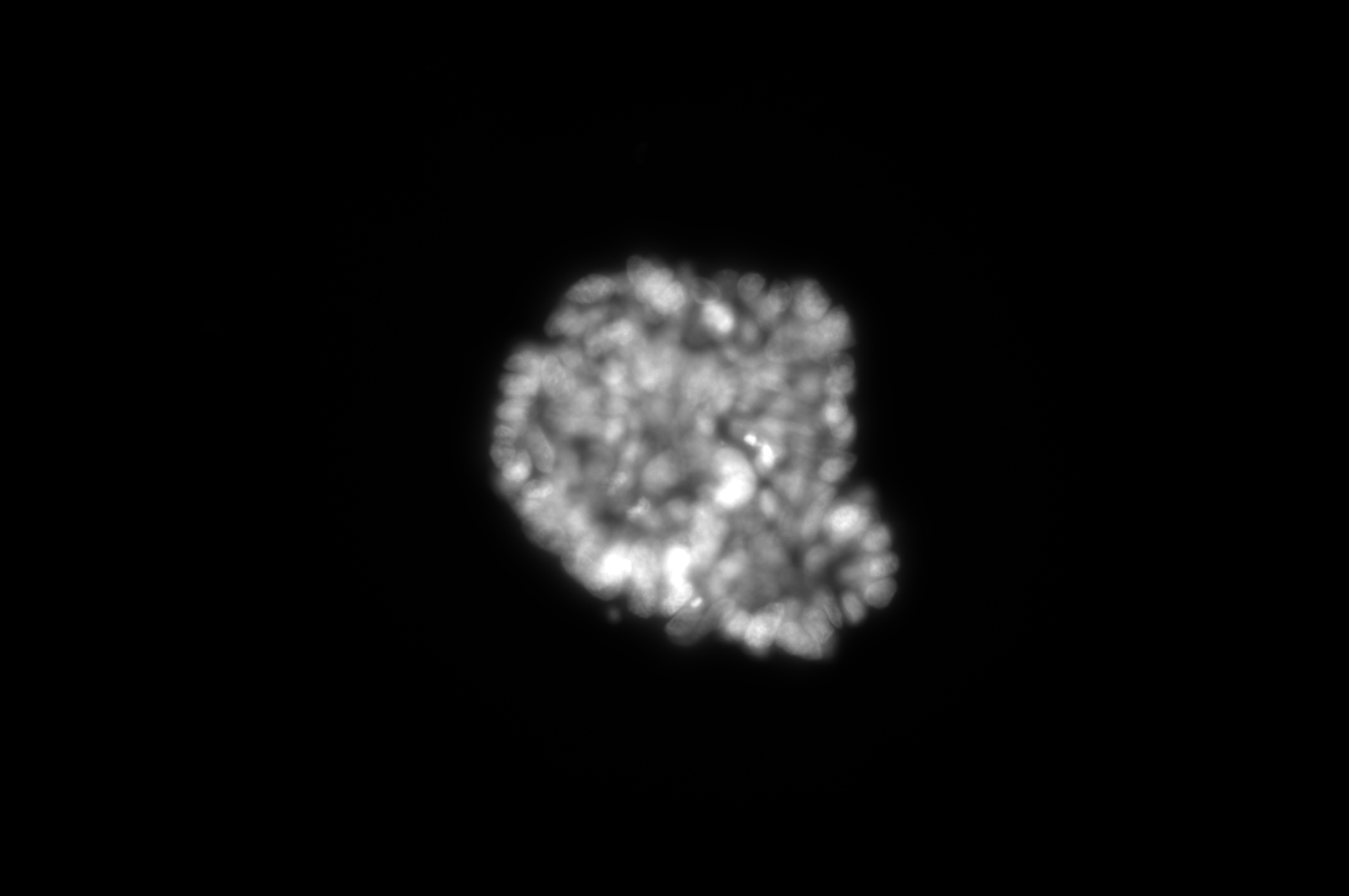

Supplement: Supplementary file 5 — Source Data Fig. 4 [file 44320_2024_11_MOESM5_ESM.zip › Figure 4/4F/Basolateral out_DAPI Phalloidin_2.tif]

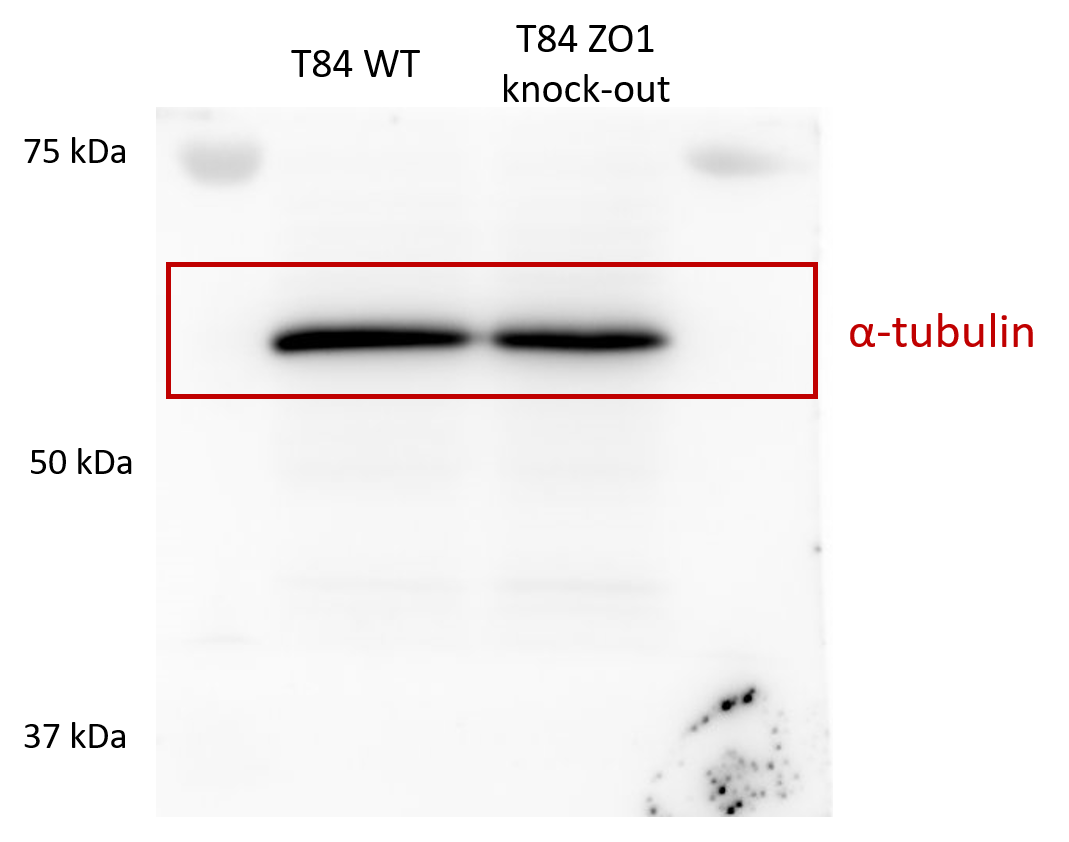

Supplement: Supplementary file 6 — Source Data Fig. 6 [file 44320_2024_11_MOESM6_ESM.zip › Figure 6/6B/a-tubulin WesternBlot.png]

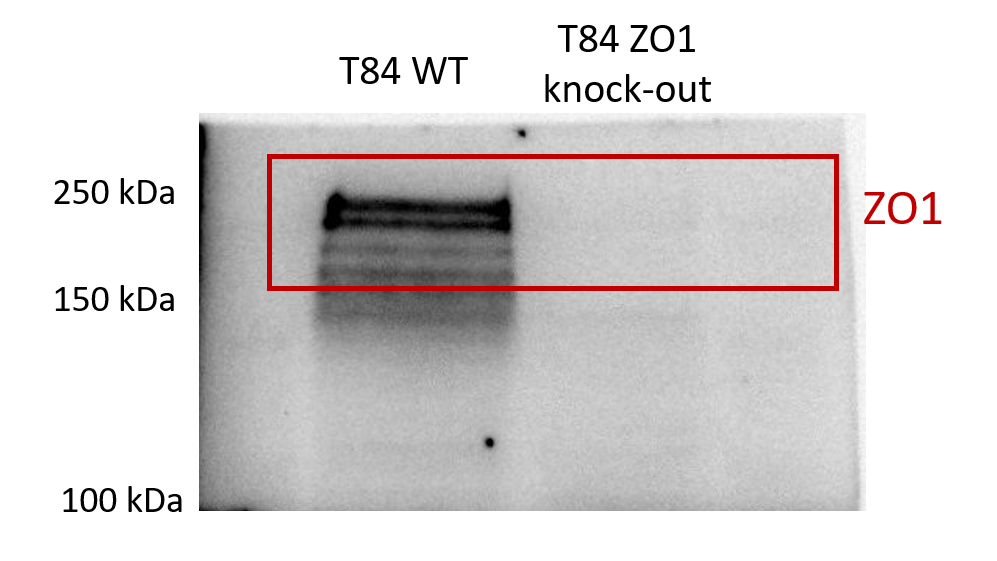

Supplement: Supplementary file 6 — Source Data Fig. 6 [file 44320_2024_11_MOESM6_ESM.zip › Figure 6/6B/ZO1 WesternBlot.png]

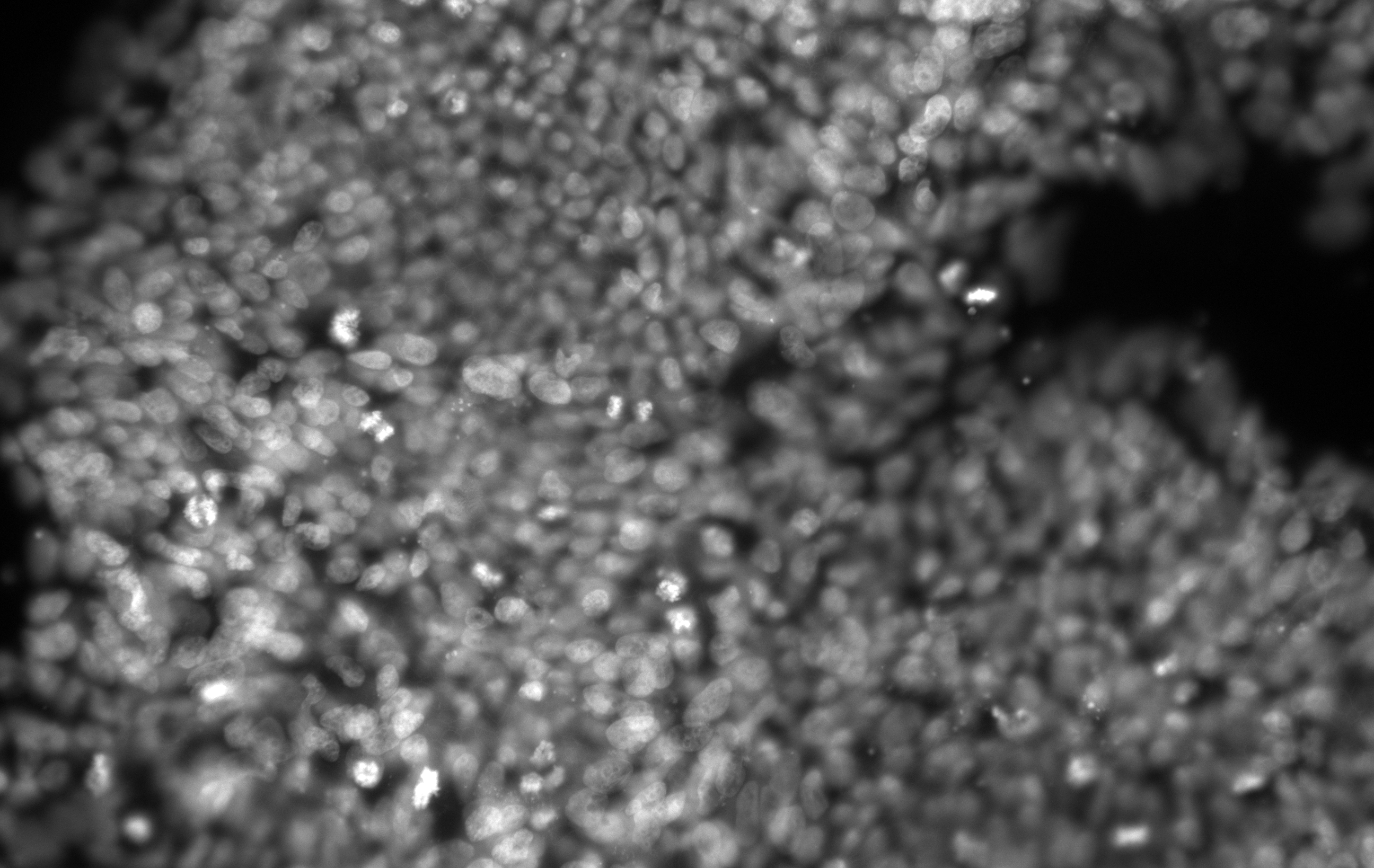

Supplement: Supplementary file 6 — Source Data Fig. 6 [file 44320_2024_11_MOESM6_ESM.zip › Figure 6/6C/T84 WT_DAPI and ZO1 stain.tif]

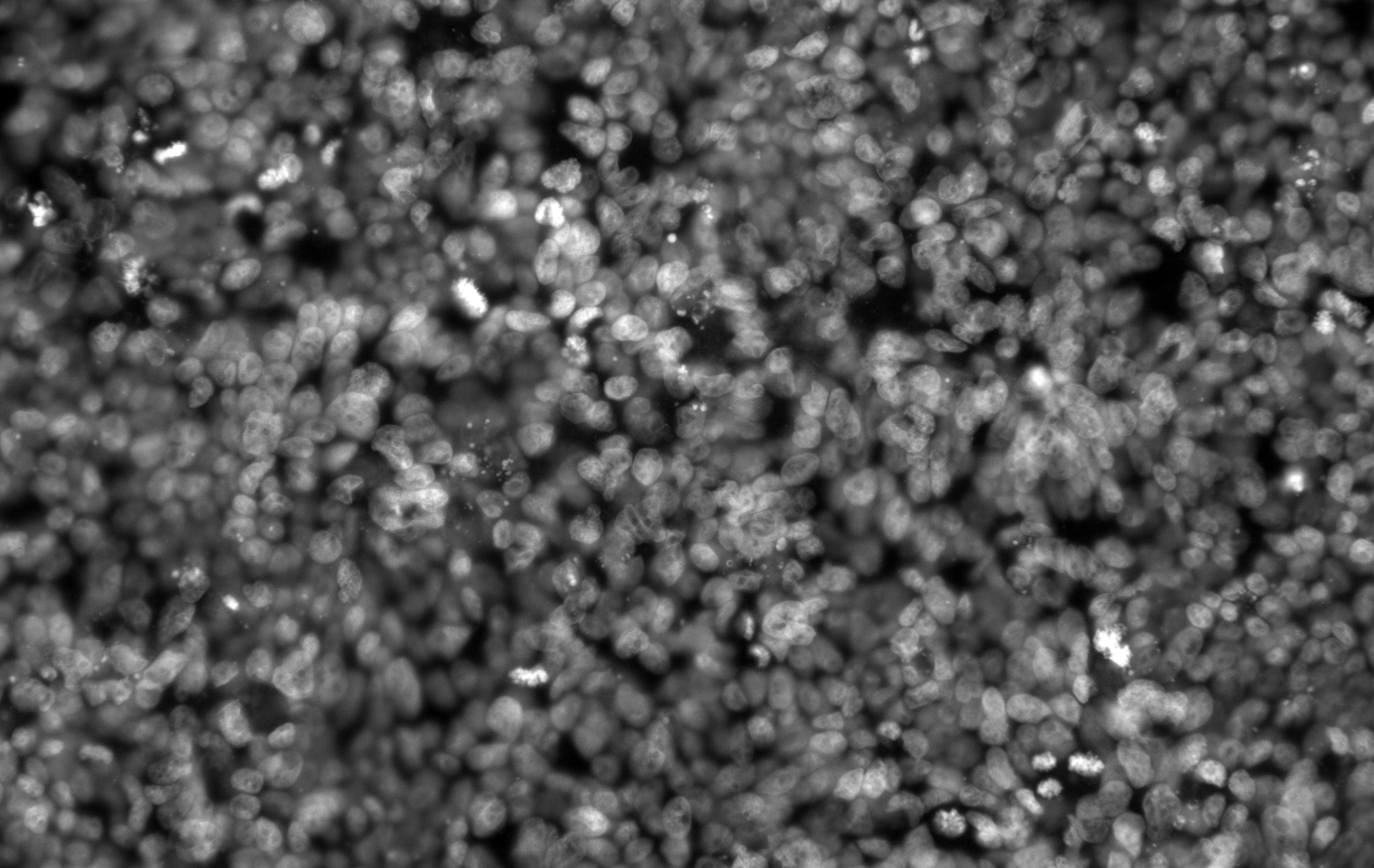

Supplement: Supplementary file 6 — Source Data Fig. 6 [file 44320_2024_11_MOESM6_ESM.zip › Figure 6/6C/T84 ZO1 KO_DAPI and ZO1 stain.tif]

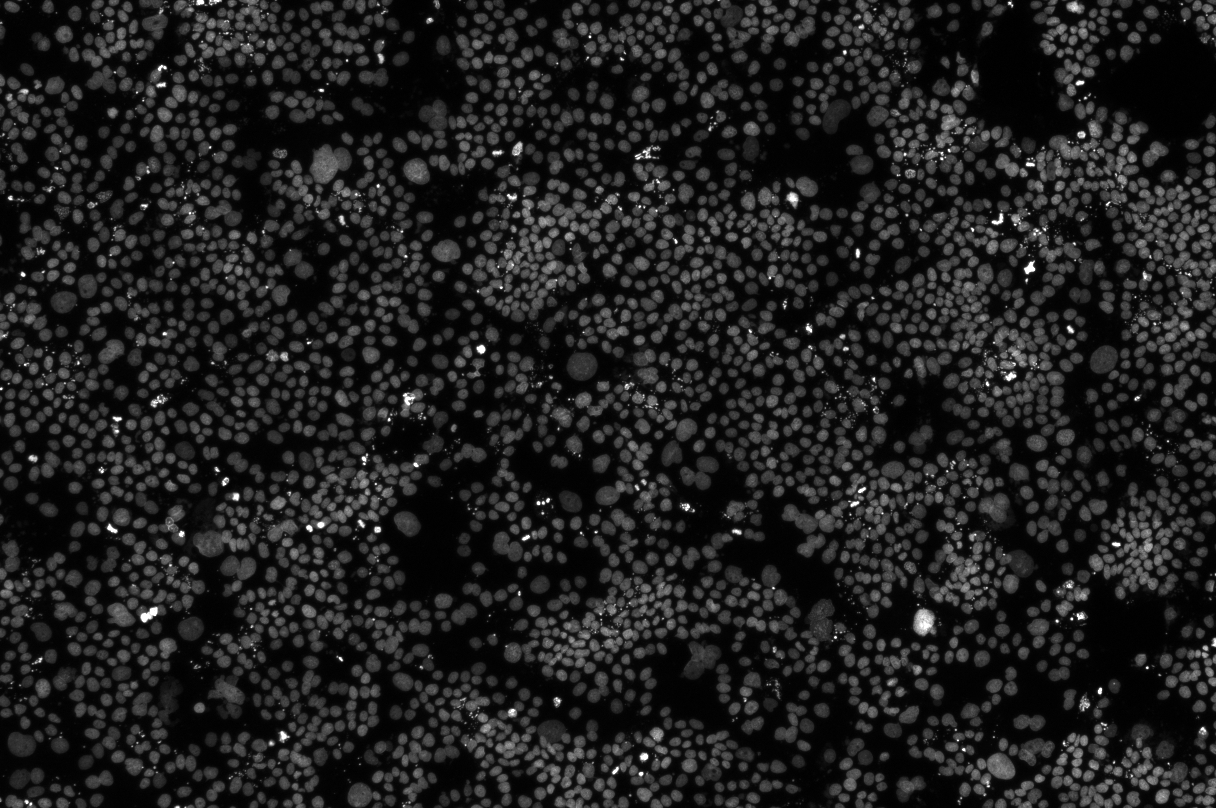

Supplement: Supplementary file 7 — Source Data Fig. 7 [file 44320_2024_11_MOESM7_ESM.zip › Figure 7/7B/high density_IFNb1 and virus.tif]

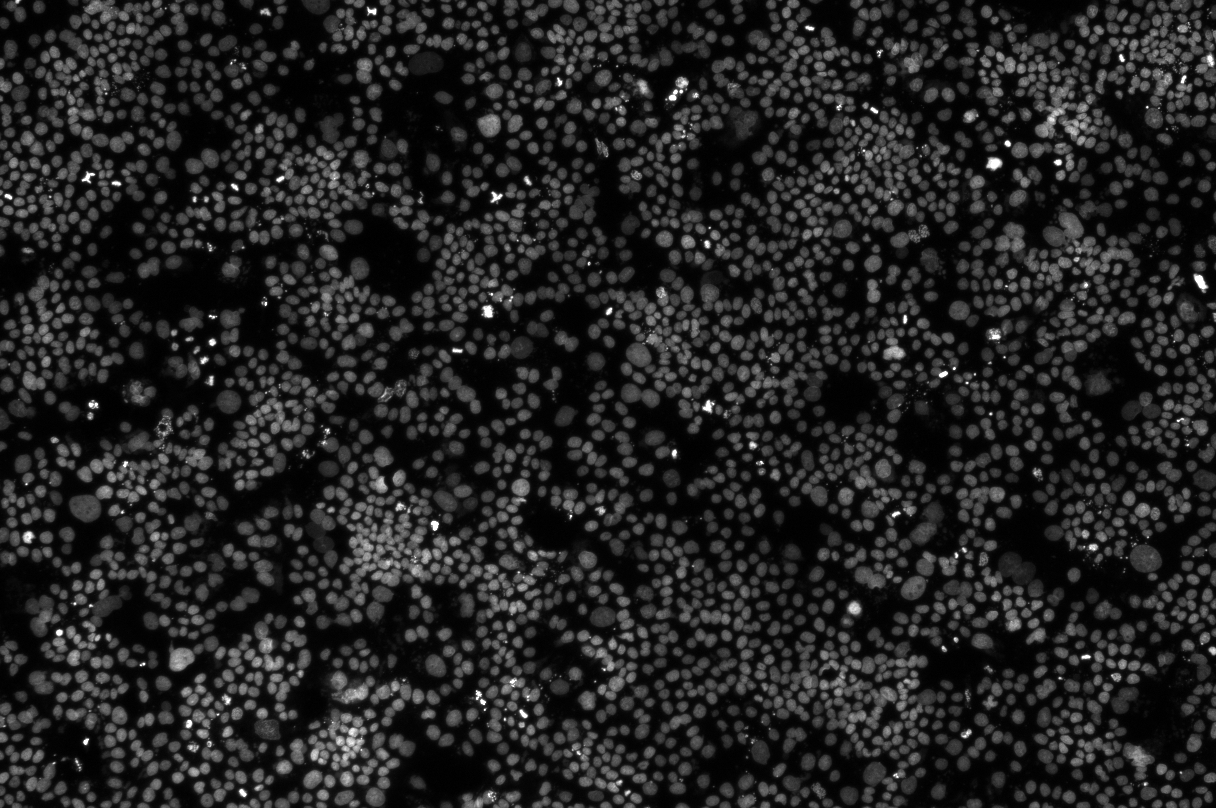

Supplement: Supplementary file 7 — Source Data Fig. 7 [file 44320_2024_11_MOESM7_ESM.zip › Figure 7/7B/high density_IFNL1-3 and virus.tif]

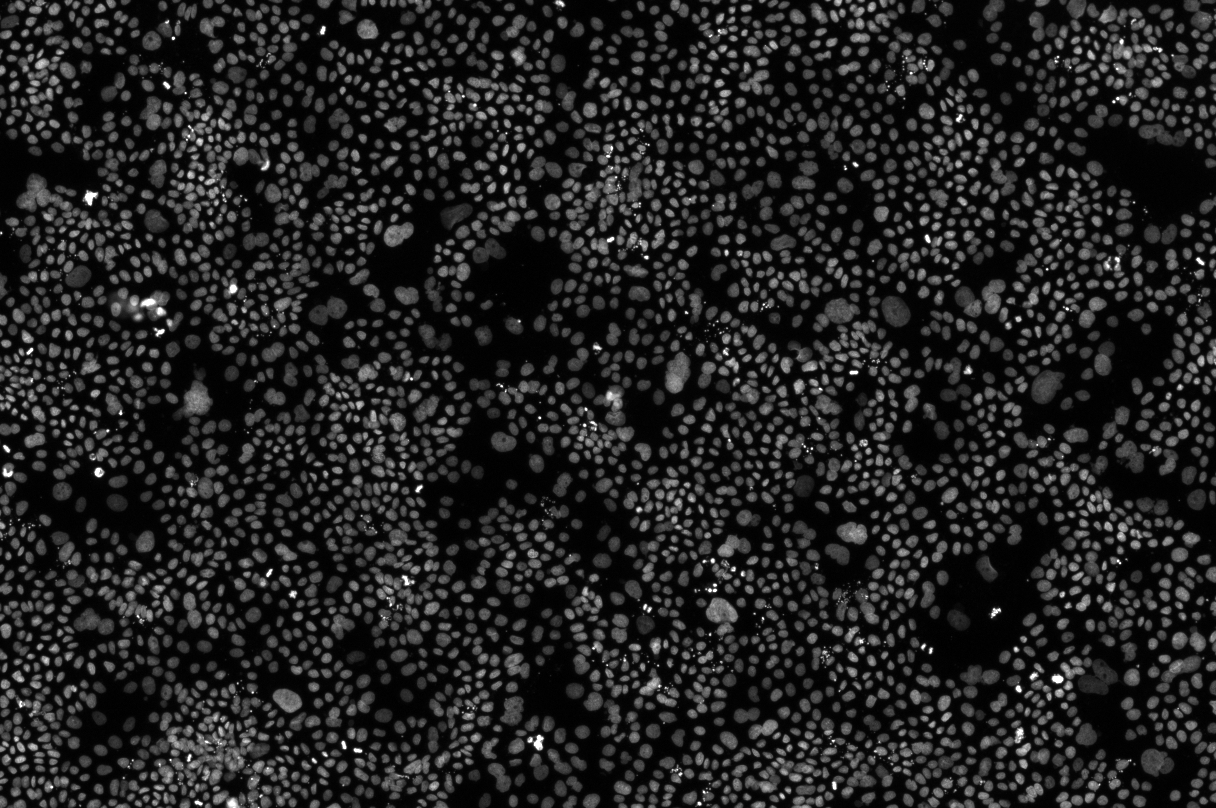

Supplement: Supplementary file 7 — Source Data Fig. 7 [file 44320_2024_11_MOESM7_ESM.zip › Figure 7/7B/high density_mock.tif]

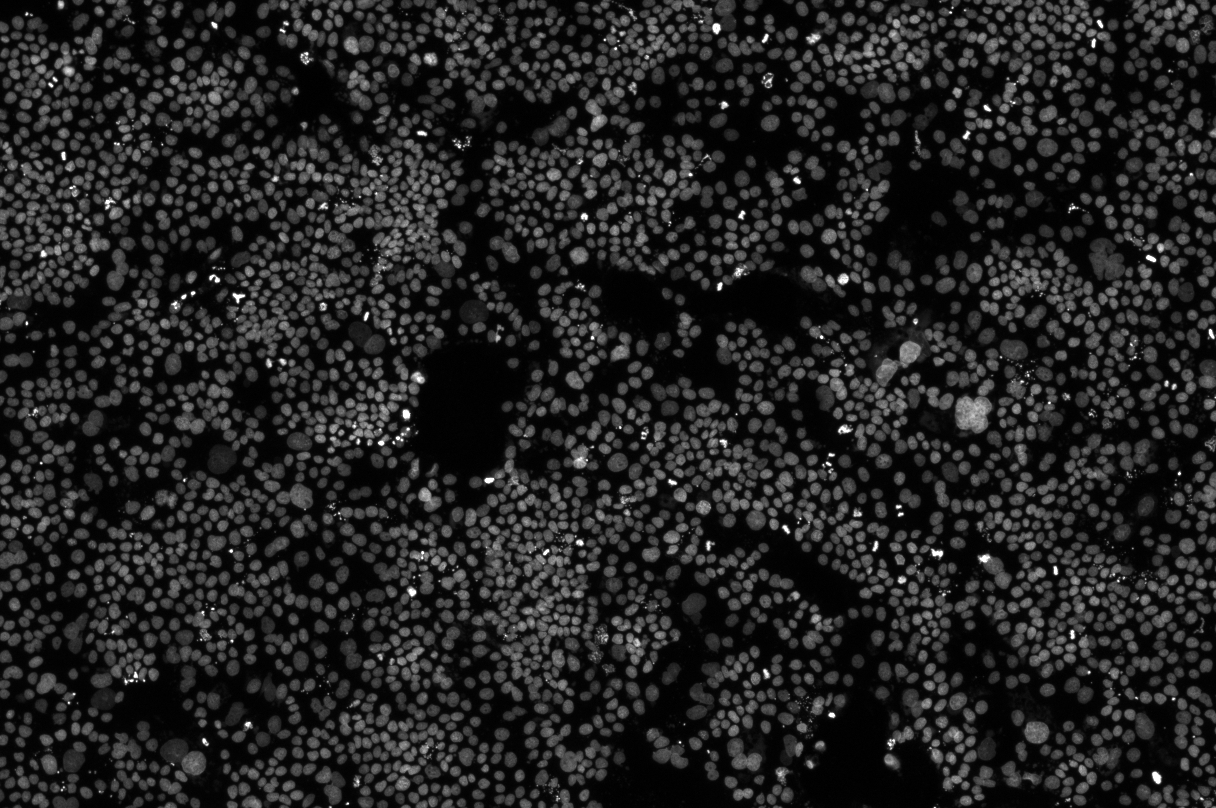

Supplement: Supplementary file 7 — Source Data Fig. 7 [file 44320_2024_11_MOESM7_ESM.zip › Figure 7/7B/high density_virus only.tif]

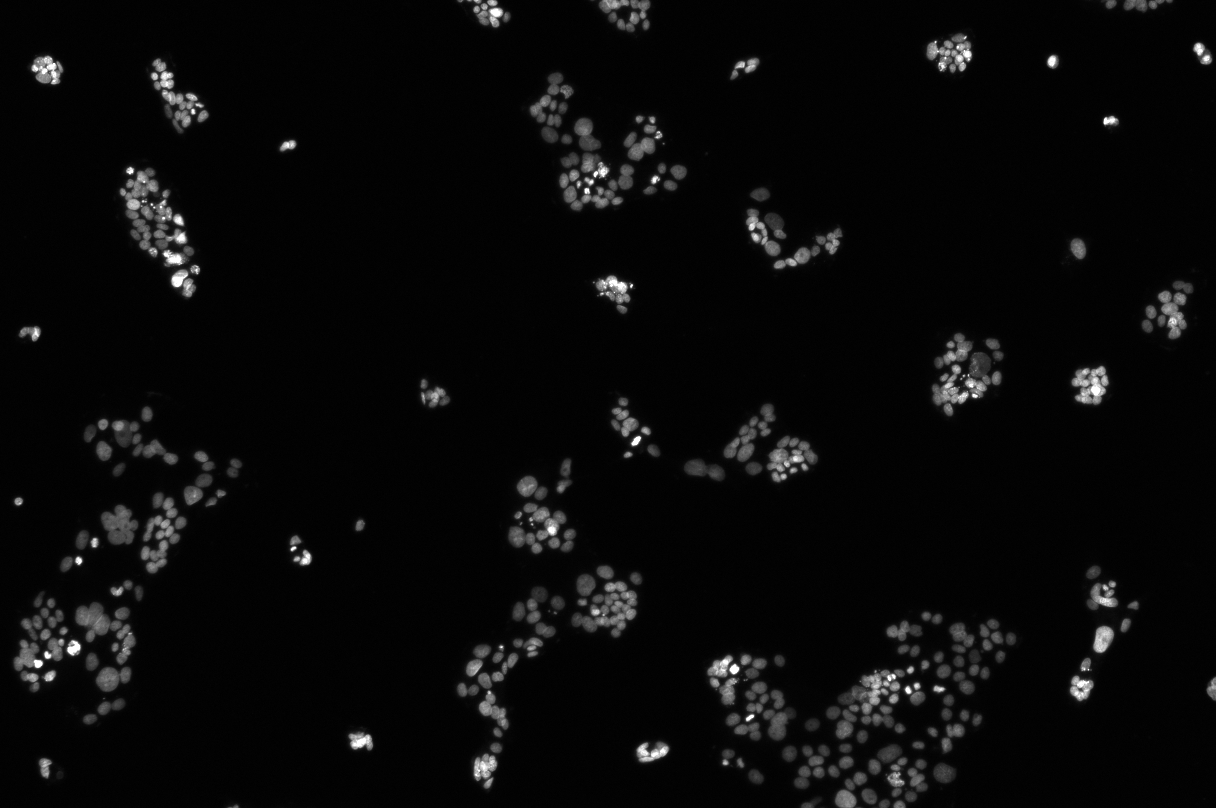

Supplement: Supplementary file 7 — Source Data Fig. 7 [file 44320_2024_11_MOESM7_ESM.zip › Figure 7/7B/low density_IFNb1 and virus.tif]

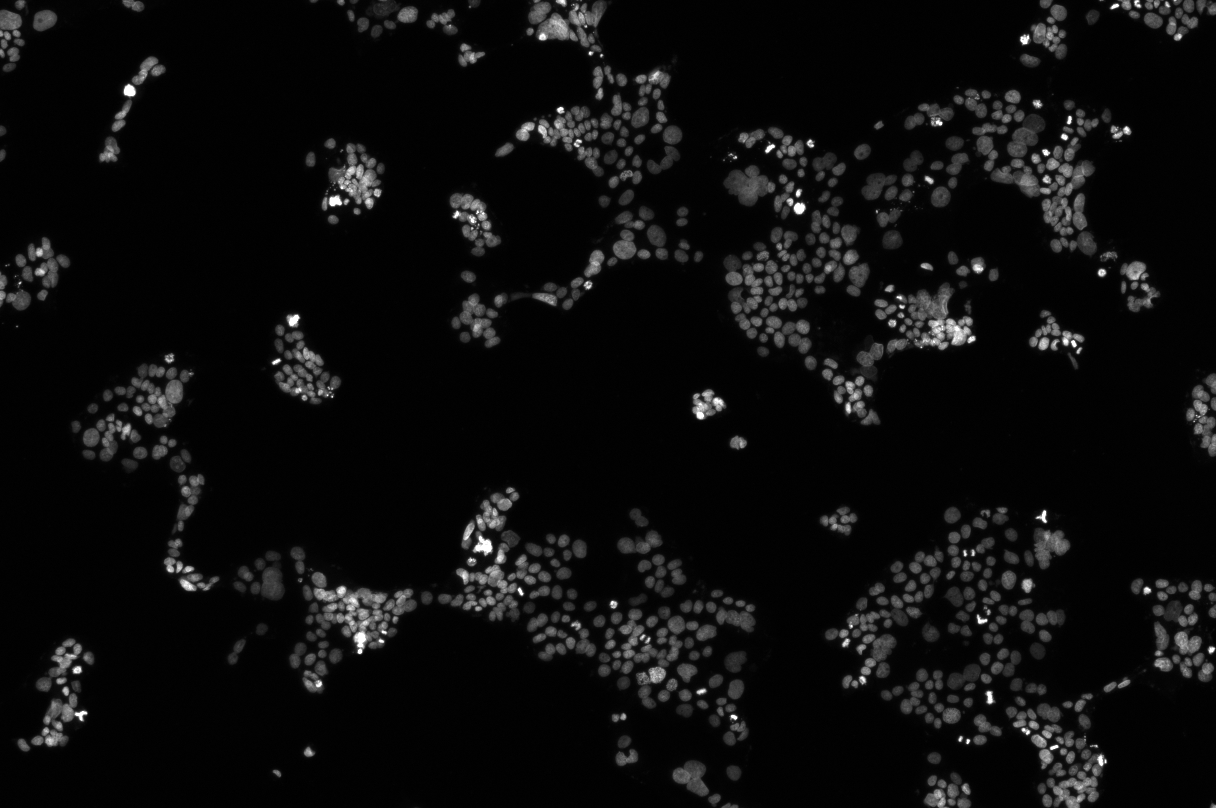

Supplement: Supplementary file 7 — Source Data Fig. 7 [file 44320_2024_11_MOESM7_ESM.zip › Figure 7/7B/low density_IFNL1-3 and virus.tif]

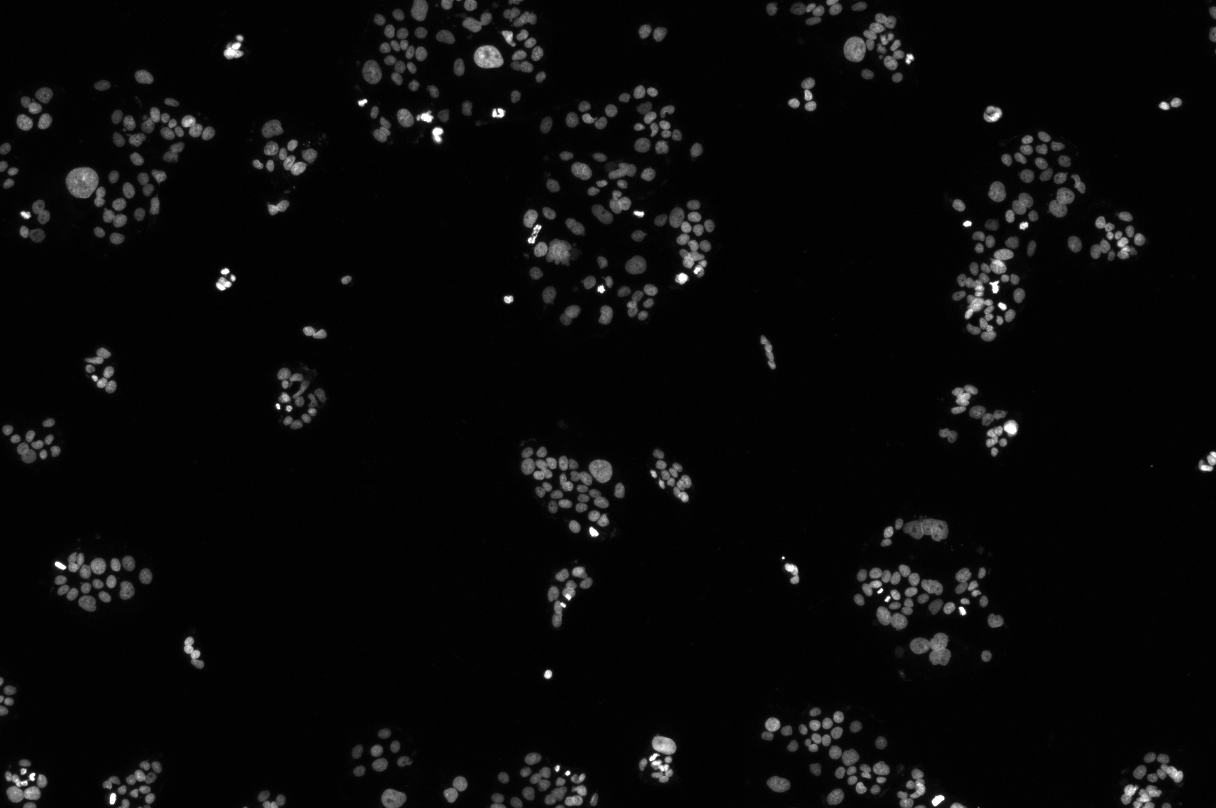

Supplement: Supplementary file 7 — Source Data Fig. 7 [file 44320_2024_11_MOESM7_ESM.zip › Figure 7/7B/low density_mock.tif]

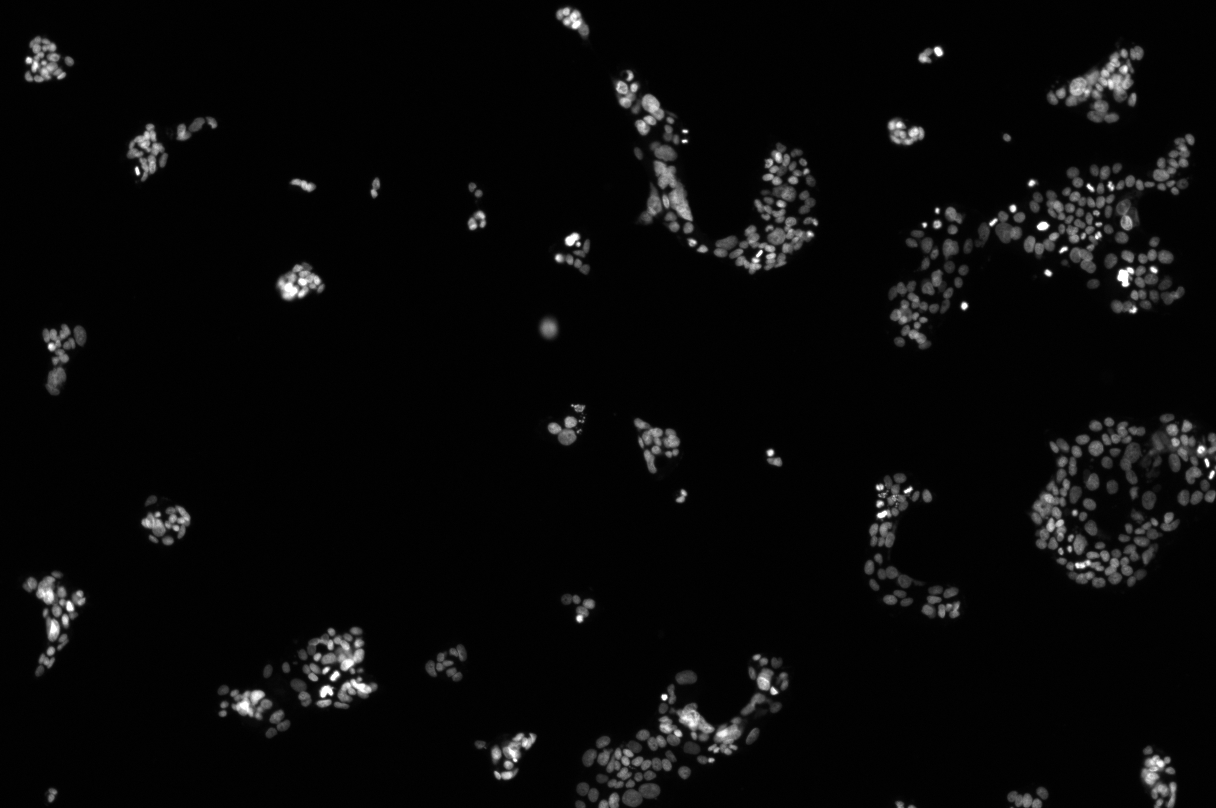

Supplement: Supplementary file 7 — Source Data Fig. 7 [file 44320_2024_11_MOESM7_ESM.zip › Figure 7/7B/low density_virus only.tif]
