# Supplementary material for: The population context is a driver of the heterogeneous response of epithelial cells to interferons
Source: Mol Syst Biol. 2024 Jan 25;20(3):7. doi: 10.1038/s44320-024-00011-2 (PMC10912784; doi:10.1038/s44320-024-00011-2)
Supplement: Supplementary file 9 — Expanded View Figures [file 44320_2024_11_MOESM9_ESM.pdf]

Expanded View Figures

Figure EV1. IFN-sensing reporter cell line and intestinal organoids seeded in 2D show heterogeneity during IFN treatment.

(**A**) Schematic depicting the T84 prom-Mx1-fp reporter cell line. Upon interaction of IFNs with their receptor, downstream signaling induces nuclear translocation of the transcription complex ISGF3. This leads to expression of the fluorescent protein under control of the ISG Mx1 promoter. The fluorescent protein accumulates in the cytosol and can be visualized by fluorescence microscopy. (**B**) Representative images showing expression of the fluorescent reporter (white) after mock, 2000 IU/mL IFNβ1, or 300 ng/mL IFNλ1-3 treatment. Nuclei are stained with DAPI (blue). $n = 3$ biological replicates. Scale bar = 100 µm. (**C**) T84-prom-Mx1-fp seeded at medium density were mock treated or treated with 2000 IU/mL IFNβ1 or 300 ng/mL IFNλ1-3 for 24 h. The positive fluorescent cells were determined for each edge degree and the mean fluorescence intensity (MFI) was measured within each positive cell. The MFI was normalized to the mock-treatment MFI of the corresponding edge degree (normalized fluorescence). (**D**) T84-prom-Mx1-fp seeded at medium density were mock treated or treated with 2000 IU/mL IFNβ1 or 300 ng/mL IFNλ1-3 for 6, 12, 24, 48, 72, and 96 h. Quantification of the percentage of total positive fluorescent cells as compared to mock-treated cells. (**E**, **F**) Ileum-derived organoids were seeded in 2-dimensions (2D) and treated apically with 2000 IU/mL IFNβ1 or 300 ng/mL IFNλ1-3. 24 h post-treatment, samples were fixed and indirect immunofluorescence was performed against ISG15 (green). Nuclei were stained with DAPI (blue). (**E**) Representative images are shown. Yellow arrows point at IFN responder cells located at the colony edges. Red arrows point at non-responder cells in the colony center. Scale bar = 100 µm. (**F**) Quantification of the ISG mean fluorescence intensity (arbitrary units (a.u.)) at the edge or the center of cell clusters. (**D**, **F**) $n \geq 3$ biological replicates, error bars indicate the standard deviation. n.s. = not significant. $P < 0.05$ *, $P < 0.01$ **, $P < 0.001$ ***, $P < 0.0001$ **** as determined by (**D**) ordinary one-way ANOVA with Dunnett's multiple comparison test using edge degree 1 as reference and (**F**) Unpaired *t* test with Welch's correction.

© The Author(s)

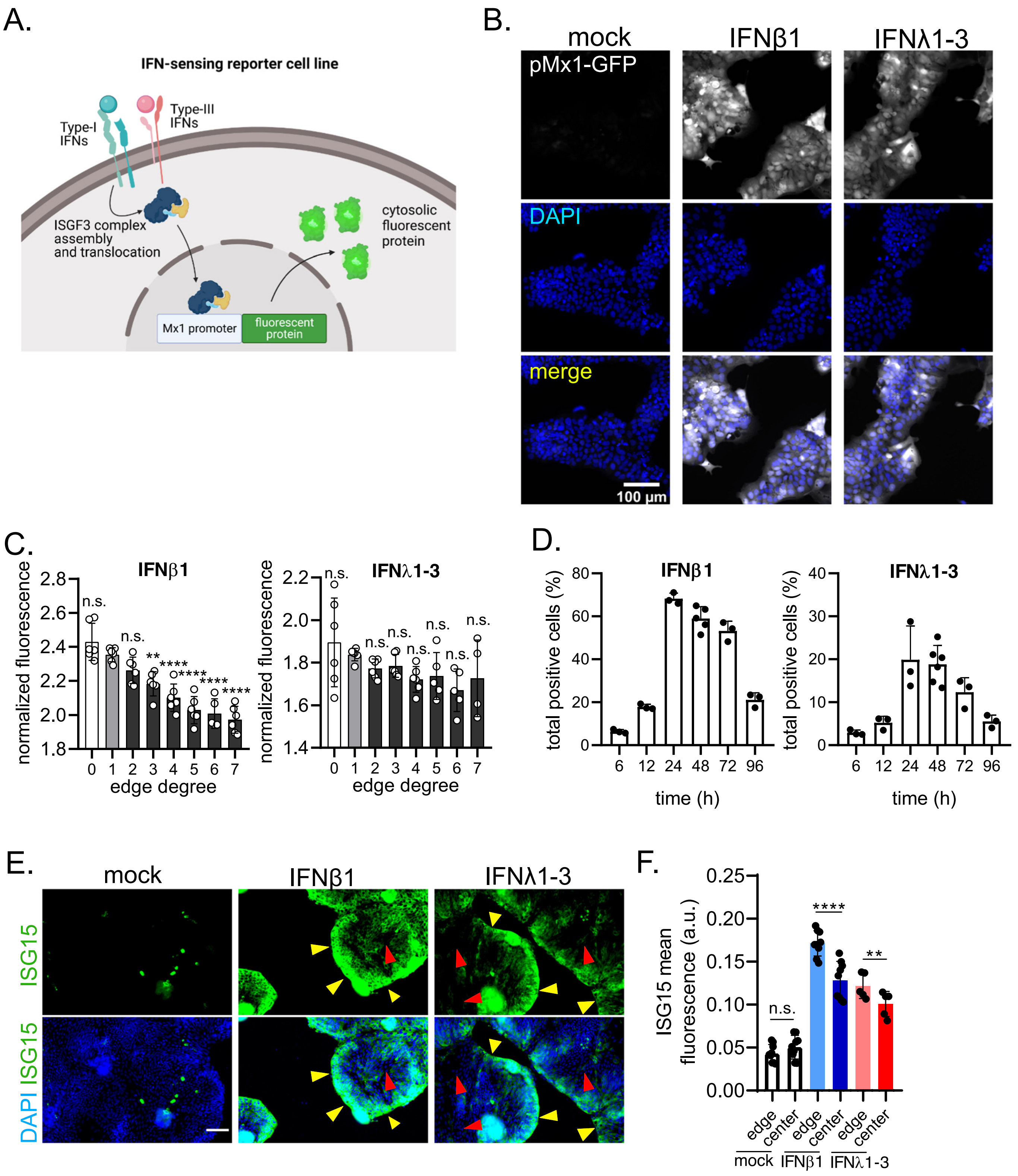
A.
IFN-sensing reporter cell line
Type-I IFNs
Type-III IFNs
ISGF3 complex assembly and translocation
cytosolic fluorescent protein
Mx1 promoter
fluorescent protein
B.
mock
IFNβ1
IFNλ1-3
pMx1-GFP
DAPI
merge
100 µm
C.
IFNβ1
IFNλ1-3
normalized fluorescence
edge degree
n.s.
D.
IFNβ1
IFNλ1-3
total positive cells (%)
time (h)
E.
mock
IFNβ1
IFNλ1-3
ISG15
DAPI ISG15
F.
ISG15 mean fluorescence (a.u.)
edge
center
mock
IFNβ1
IFNλ1-3
n.s.
****
**

 © The Author(s)

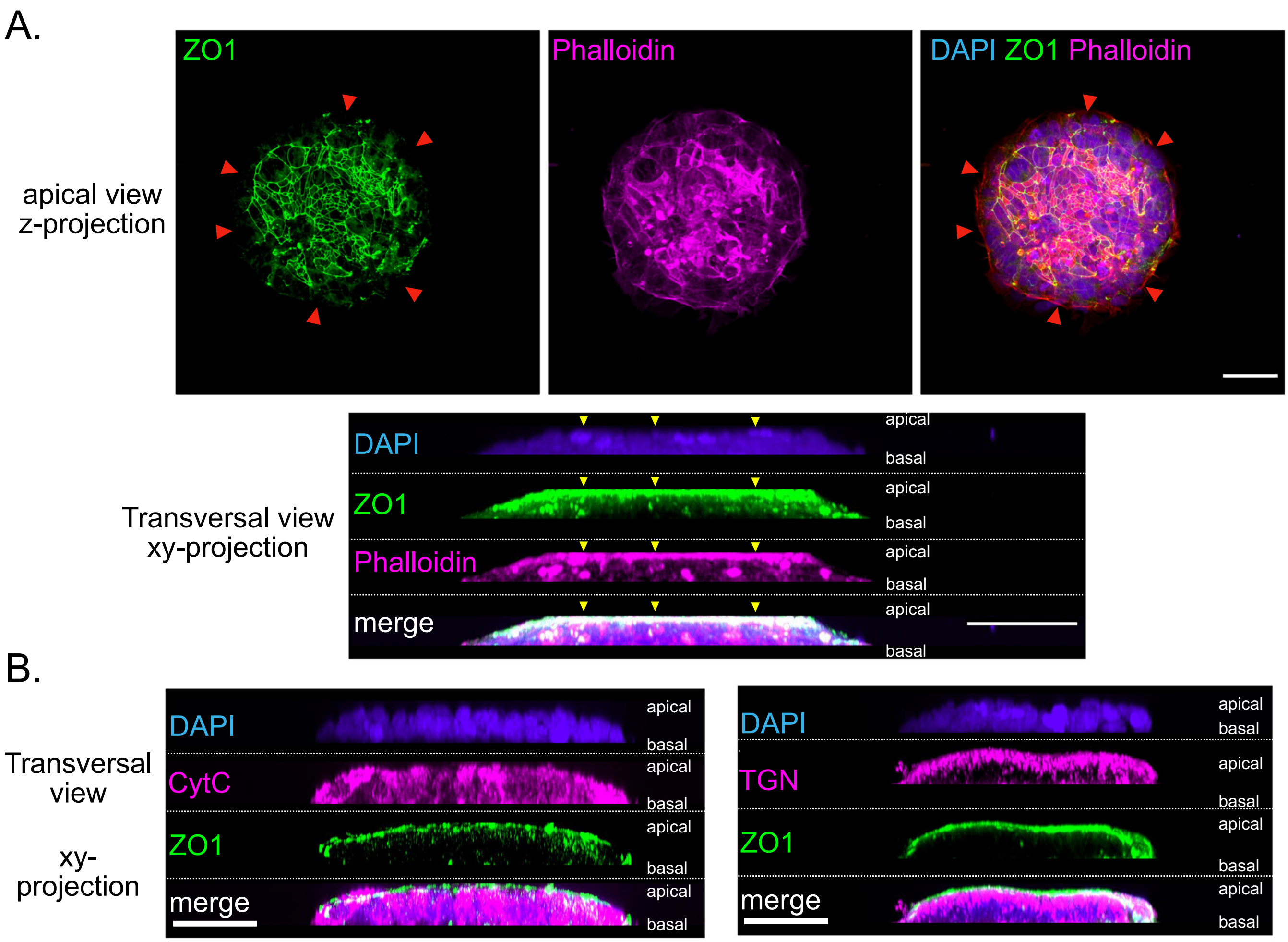

Figure EV2. Protein expression and protein localization in IEC populations seeded on micropatterns.

T84 WT cells were seeded on micropatterns as populations and fixed. Immunostaining was performed against a variety of proteins. Cells were imaged with spinning disc microscopy at different focal-planes (Z-stack), and visualized as apical view (with z-projection) or transversal view (xy-projection). (**A**) Representative images showing ZO1 protein (green) and Phalloidin-647 (magenta) which stains for F-actin. Red arrows point out edge cells lacking ZO1 protein, and yellow arrows show localization of ZO1 and F-actin to the apical side of the populations. (**B**) ˆRepresentative images showing the mitochondria marker Cytochrom C (CytC), Trans-Golgi Network (TGN) and ZO1 in epithelial cell populations along the Z-axis. (**A**, **B**) Cell nuclei were stained with DAPI (blue). Scale bar = 50 μm.

© The Author(s)

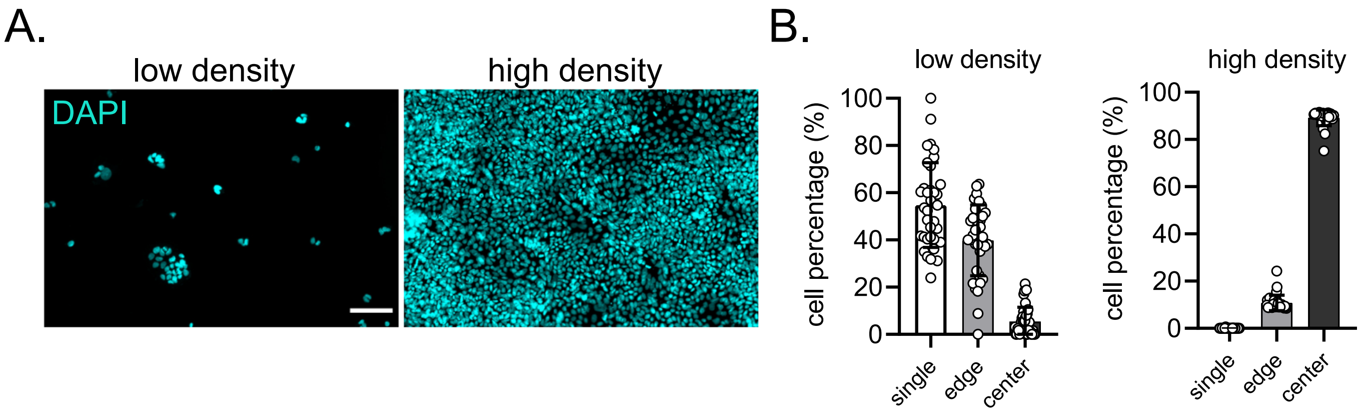

Figure EV3. Cell seeding at high and low density.

T84 WT cells were seeded at high and low density. (**A**) Representative images showing cell nuclei stained with DAPI (blue). Scale bar = 100 µm. (**B**) The DBSCAN-CellX App was used to determine the percentage of cells localized at the edge and the center for low and high density seeding conditions. $n \geq 3$ biological replicates, error bars indicate the standard deviation.

 © The Author(s)

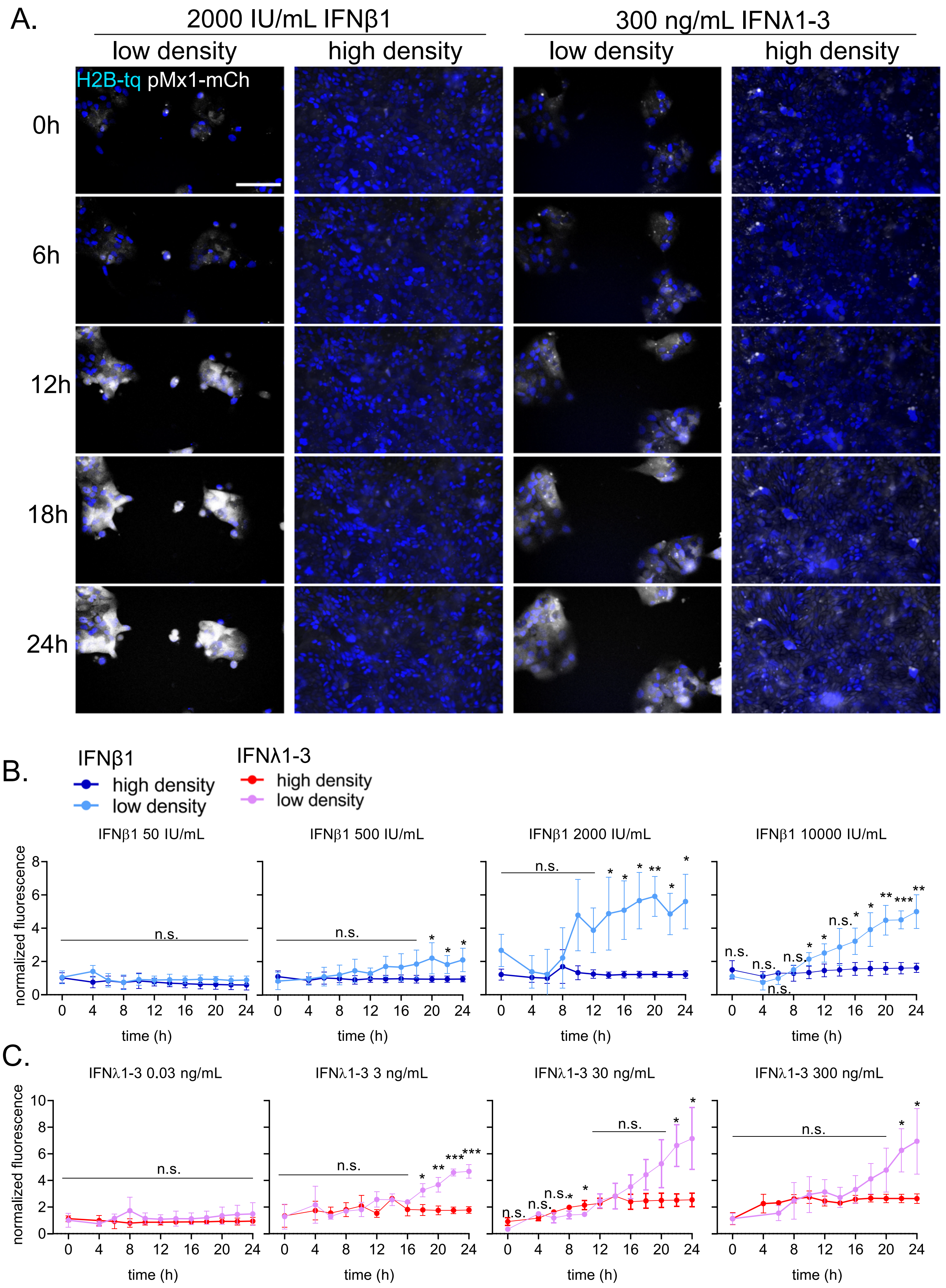
A.
2000 IU/mL IFNβ1
300 ng/mL IFNλ1-3
low density
high density
low density
high density
H2B-tq pMx1-mCh
0h
6h
12h
18h
24h
B.
IFNβ1
high density
low density
IFNλ1-3
high density
low density
IFNβ1 50 IU/mL
IFNβ1 500 IU/mL
IFNβ1 2000 IU/mL
IFNβ1 10000 IU/mL
normalized fluorescence
time (h)
n.s.
C.
IFNλ1-3 0.03 ng/mL
IFNλ1-3 3 ng/mL
IFNλ1-3 30 ng/mL
IFNλ1-3 300 ng/mL
normalized fluorescence
time (h)

Figure EV4. Temporal response of cells at high and low density to IFN treatment.

T84-prom-Mx1-fp cells at high or low density were treated with increasing concentrations of IFNβ1 and IFNλ1-3. Live cell fluorescence imaging was performed at an interval of 2 h for 24 h. (**A**) Representative images for selected time-points showing expression of the reporter prom-Mx1-mCherry (pMx1-mCh) in white. Nuclei are visualized by expression of H2B-turqiouse. Scale bar = 100 μm. (**B**, **C**) The mean fluorescence intensity (MFI) of the reporter expression within each cell was averaged for each density and normalized to the mock MFI of each time-point (fold change) for (**B**) IFNβ1 and (**C**) IFNλ1-3. $n = 3$ biological replicates. n.s = not significant, error bars indicate the standard deviation. $P < 0.05$ *, $P < 0.01$ **, P < 0.001 ***, $P < 0.0001$ **** as determined by Unpaired t test with Welch's correction between high and low density for each time-point.

 © The Author(s)

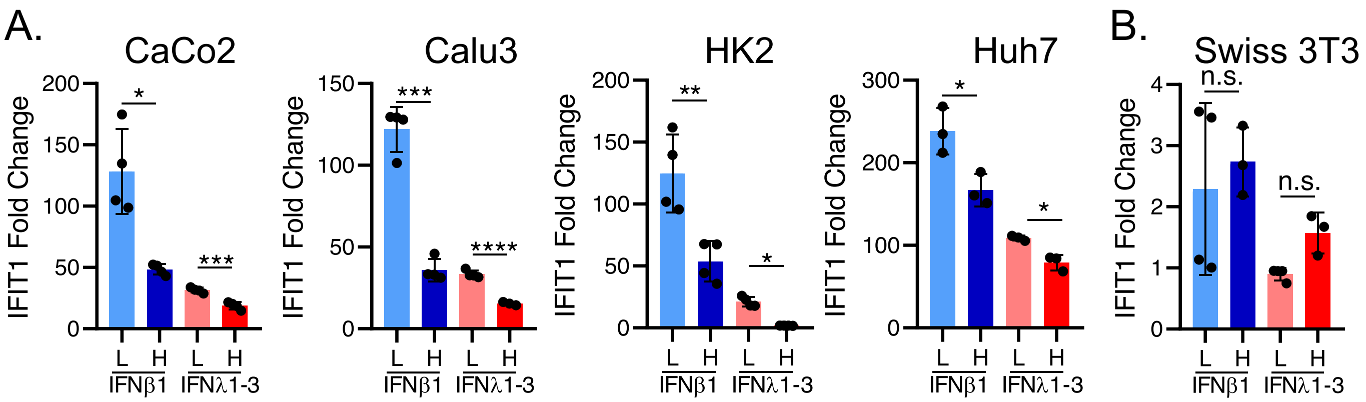

Figure EV5. Effect of density and basolateral IFN receptor localization in epithelial and non-epithelial cells.

(**A**) Epithelial and (**B**) non-epithelial cells were seeded at high (H) and low (L) density. Cells were mock treated, or treated with 2000 IU/mL IFNβ1 or 300 ng/mL IFNλ1-3. 24 h post IFN treatment, RNA was harvested to evaluate the transcription of the representative ISGs IFIT1 using RT-q-PCR. ISG relative expression was normalized to the mock-treated cells (fold change). $n \geq 3$ biological replicates, error bars indicate the standard deviation. n.s. = not significant. $P < 0.05$ *, $P < 0.01$ **, $P < 0.001$ ***, $P < 0.0001$ **** as determined by Unpaired t test with Welch's correction.

© The Author(s)

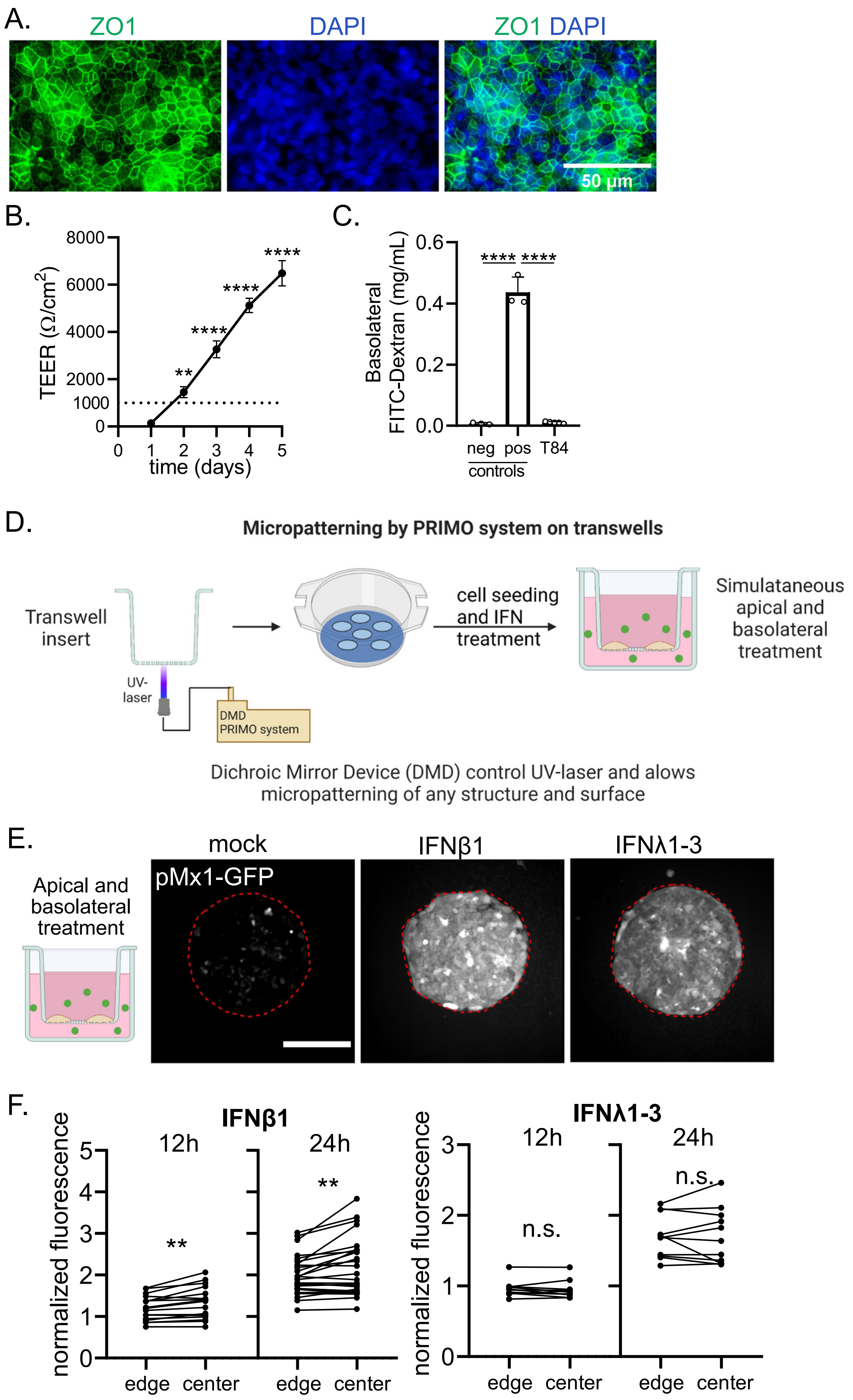
A.
ZO1
DAPI
ZO1 DAPI
50 µm
B.
TEER (Ω/cm²)
8000
6000
4000
2000
1000
0
0 1 2 3 4 5
time (days)
**
****
****
****
****
C.
Basolateral FITC-Dextran (mg/mL)
0.6
0.4
0.2
0.0
**** ****
neg pos T84
controls
D.
Micropatterning by PRIMO system on transwells
Transwell insert
UV-laser
DMD PRIMO system
cell seeding and IFN treatment
Simulataneous apical and basolateral treatment
Dichroic Mirror Device (DMD) control UV-laser and alows micropatterning of any structure and surface
E.
Apical and basolateral treatment
mock
IFNβ1
IFNλ1-3
pMx1-GFP
F.
IFNβ1
12h
24h
normalized fluorescence
**
**
edge center
edge center
IFNλ1-3
12h
24h
n.s.
n.s.
edge center
edge center

 © The Author(s)

Figure EV6. Transwell system to grow a semipermeable monolayer of polarized cells and to seed populations that are accessible from the basolateral side.

(**A–C**) T84 cells were seeded on transwell inserts to allow for a polarized monolayer formation. (**A**) 5 days post seeding, cells were fixed, and indirect immunofluorescence was performed against the junctional complex protein ZO1 (green). Nuclei were stained with DAPI (blue). Representative image is shown. Scale bars = 50 μm. $n = 3$ biological replicates. (**B**) Formation and integrity of the monolayer was followed by measuring the transepithelial electrical resistance (TEER) (Ω/cm^2) over 5 days. Values > 1000 Ω/cm^2 (dotted line) shows that cells established a polarized monolayer formation. $n = 3$ biological replicates. (**C**) 5 days post seeding, after reaching a polarized monolayer, the integrity of the monolayer was confirmed by the FITC-Dextran permeability assay. Diffusion of FITC-Dextran from the apical to the basolateral compartment was measured and expressed as concentration (mg/mL) of FITC-Dextran in the basolateral compartment after 3 h incubation. Positive control (pos) was the maximum diffusion possible and the negative control (neg) was medium only without FITC-Dextran. (**D**) Micropatterning of transwell inserts: Schematic depicting the micropatterning on transwell membranes using the PRIMO system (Alvéole Lab, www.alveolelab.com). (**E**, **F**) T84 prom-Mx1-fp cells were seeded on micropatterned transwell membranes. Cells were mock treated, or treated simultaneously from the apical and basolateral side with 2000 IU/mL IFNβ1 or 300 ng/mL IFNλ1-3. Cells were fixed at 0 h, 12 h, and 24 h post treatment and fluorescent imaging was performed. (**E**) Representatives images showing treated T84 cell populations. The red line represents the edge of the patterns. Expression of the fluorescent reporter is depicted in white. Scale bar = 100 μm. (**F**) The reporter expression was quantified by measuring the mean fluorescence intensity (MFI) at the edge and the center of a population at 12 h or 24 h post treatment, and normalizing it to the corresponding 0 h post treatment at the edge and center, respectively. Each dot is one cell population (seeded on one micropattern), lines connect edge and center of the same cell population. (**B**, **C**, **F**) $n \geq 3$ biological replicates, in (**B**, **C**) error bars indicate the standard deviation. n.s. = not significant, $P < 0.05$ *, $P < 0.01$ **, $P < 0.001$ ***, $P < 0.0001$ **** as determined by (**B**, **C**) ordinary one-way ANOVA with Dunnett's multiple comparison test using (**B**) day 1 or (**C**) the positive control as reference, and (**F**) Paired *t* test.

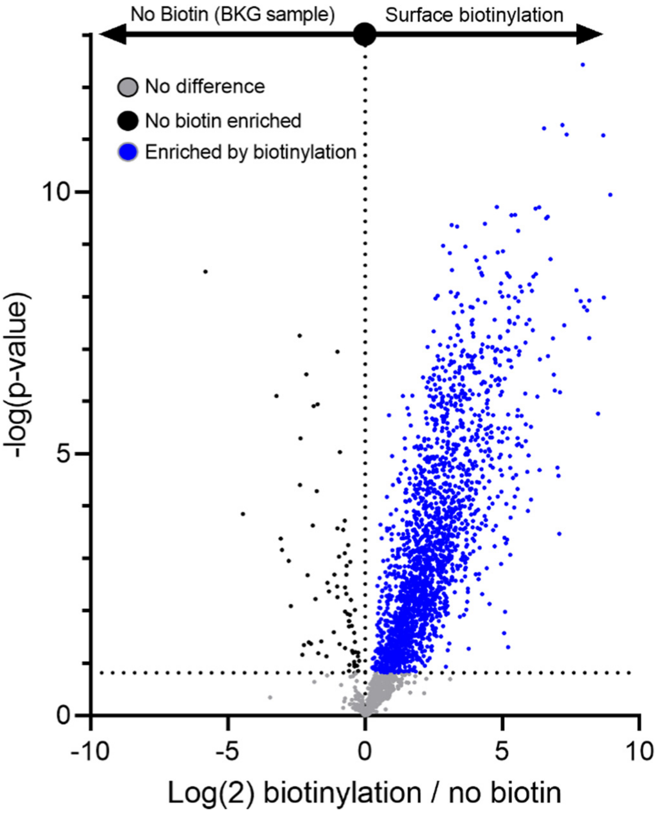

Figure EV7. Enrichment via biotinylation of the surface proteome.

T84 WT cells were grown as a polarized monolayer on transwell inserts. Apical or basolateral surface proteins were biotinylated by addition of cell non-permeable reactive NHS-biotin to the apical or basolateral compartment of the transwell insert, respectively. Biotinylated proteins were pulled down using streptavidin beads and identified by mass spectrometry. For the analysis, biotinylated proteins were filtered by using a non-biotinylated control sample by the volcano plot function of the Perseus software, where Biotinylated apical and Biotinylated basolateral samples are matched against the non-biotinylated control using a *t*-test with FDR of 0.05 and an s0 = 0.1. Volcano plot showing enrichment via biotinylation of the surface proteome. $n = 4$ biological replicates.

 © The Author(s)
