# Supplementary material for: The population context is a driver of the heterogeneous response of epithelial cells to interferons
Source: Mol Syst Biol. 2024 Jan 25;20(3):7. doi: 10.1038/s44320-024-00011-2 (PMC10912784; doi:10.1038/s44320-024-00011-2)
Supplement: Supplementary file 8 — Peer Review File [file 44320_2024_11_MOESM8_ESM.pdf]

molecular systems biology

The population context is a driver of the heterogeneous response of epithelial cells to interferons

Camila Metz-Zumaran, Zina Uckeley, Patricio Doldan, Francesco Muraca, Yagmur Keser, Pascal Lukas, Benno Kuropka, Leonie Küchenhoff, Soheil Rastgou Talemi, Thomas Hoefer, Christian Freund, Elisabetta Ada Cavalcanti-Adam, Frederik Graw, Megan Stanifer, and Steeve Boulant
DOI: 10.15252/msb.202311778

Corresponding author(s): Steeve Boulant (s.boulant@ufl.edu) , Megan Stanifer (m.stanifer@ufl.edu)

Review Timeline:

Submission Date:	19th May 23
Editorial Decision:	26th Jun 23
Appeal Received:	24th Jul 23
Editorial Decision:	26th Jul 23
Revision Received:	27th Oct 23
Editorial Decision:	8th Dec 23
Revision Received:	31st Dec 23
Accepted:	4th Jan 24

Editor: Maria Polychronidou

Transaction Report:

(Note: With the exception of the correction of typographical or spelling errors that could be a source of ambiguity, letters and reports are not edited. Depending on transfer agreements, referee reports obtained elsewhere may or may not be included in this compilation. Referee reports are anonymous unless the Referee chooses to sign their reports.)

26th Jun 2023

RE: Manuscript MSB-2023-11778, Population context drives cell-to-cell variability in interferon response in epithelial cells

Dear Steeve,

Thank you again for submitting your work to Molecular Systems Biology. We have now heard back from the three referees who agreed to evaluate your study. As you will see below, the reviewers raise substantial concerns on your work, which unfortunately preclude its publication in Molecular Systems Biology.

The reviewers appreciate that the addressed topic seems relevant. However, they are not convinced that the main conclusions are well supported and they think that as it stands the broader relevance of the presented observations for understanding IFN responses remains unclear. As such, the reviewers indicated that they do not support publication in Molecular Systems Biology.

Taken together and given the substantial concerns raised by the reviewers, I am afraid that we cannot offer to publish the study. I am sorry that the review of your work did not result in a more favorable outcome on this occasion, but I hope that you will not be discouraged from submitting future work to Molecular Systems Biology. In any case, thank you for the opportunity to examine this work.

Kind regards,

Maria

Maria Polychronidou, PhD
Senior Editor
Molecular Systems Biology

Reviewer #1:

Metz-Zumaran et al presents analysis to address whether a position of a cell with respect to the neighboring cells affects single cell response to interferons. They report that in populations of intestinal epithelial cells, the cells on the population edge are more responsive than cells within the population. They show that interferon responsiveness and antiviral activity is dependent on cell density. The use of the micropatterning is a nice approach as it provides for a more controlled system for culturing cells in uniform population sizes. However, conclusions are drawn about the spatial impact of interferon responsiveness using cell density experiments without quantitative analysis of the number of center and edge cells within the various density conditions. Additionally, conclusions about interferon responsiveness due to the basolateral location of the IFN receptors have been made without sufficient analysis of the receptors or perturbation studies to address the cellular location of the receptors. Since much of the analysis is lacking high temporal resolution (many of the studies only have 1 or 2 timepoints) and responsiveness occurs at low cell density (a condition that is contrary to the barrier role of intestinal epithelial cells which is also stated in the introduction) it is not clear how relevant or transferable such findings will be for in vivo interferon responses. As such, the conclusions are not compelling.

Major points
(i) Type I and III interferons have different temporal dynamics, so a single timepoint is not sufficient for comparing peak expression between the conditions (Fig 1). PMID: 30485383
(ii) The images used for the analysis of edge vs center cells and edge degree should be shown (Fig 1B) and not just a schematic.
(iii) Figure 1: Only 20% of cells on edge and 25% of singles respond indicating that other factors are more important than edge distance.
(iv) Need more detail about how cells are grown in these conditions and the impact on cell growth, proliferation, survival, and basal ISG levels using the micropatterning approach. (Fig 2)
(v) More quantitative analysis with percentages of edge cells vs center cells at high and low cell densities should be provided. (Fig 3)
(vi) Since making claims about cell location (edge vs center), should include images of the cultures seeded at the different cell densities instead of schematics.
(vii) The signaling analysis at 1 hour only assumes that different conditions will peak at the same time. This is not a good assumption and multiple time points should be assessed. (Fig 3C)
(viii) Should have conditions with only basolateral and only apical stimulation to draw conclusions about the contribution of

basolateral stimulation to increase activity of center cells specifically. (Fig 4B,C)
(ix) Provide list of proteins from analysis. (Fig 5C)
(x) Should demonstrate if ZO1 KO affected IFNAR or IFNLR expression levels or trafficking of receptors to apical side? (Fig 6)
(xi) Data presented in Fig 6 does not demonstrate IFN diffusion across cells as apical vs basolateral IFN levels were not measured, but this claim is made: "These results demonstrate that the tight junction protein ZO1 restricts the paracellular diffusion of IFN between polarized T84 cells..."
(xii) Linking receptor accessibility to IFN responsiveness is done with limited data (i.e. mass spec with confusing Apical/Basolateral ratios). Could use confocal or other imaging with z-stack and measure known markers for apical vs basolateral location.
(xiii) The ratio used for the receptor analysis is not clear: It is named "Apical/Basolateral ratios", but it was calculated based on the apical LFQ signal divided by the total LFQ signal. Why was the basolateral LFQ signal used instead of the total LFQ signal?
(xiv) Not sure how relevant some of these questions and experiments are to physiological IFN responses in the intestines since there are no "edge" and "center" cells lining the gut. In the introduction, it is mentioned that the gut is continuous to avoid pathogens invading into other parts of the body.

Minor points
(i) The definition of population context used in this manuscript feels like a narrow view as it doesn't take into account epithelial cell type differences, tissue structure (intestines are uniquely shaped), etc. that are seen in vivo.
(ii) There are a few typos throughout the manuscript. Page 5: "downstream 'of' the receptors". Page 10: "population or of IECs seeded".
(iii) Figure 1: What is the percentage of total responders?
(iv) Figure 3: Were the same densities from the experiment in figure 1 used for this experiment?
(v) Figure 3 legend text should include all times points when RNA was harvested; graphs indicate 0, 6, 12, and 24 hrs.
(vi) How were the concentrations of interferons used determined? This is especially important when comparing dose-independent gene expression.

Reviewer #2:

Metz-Zumaran et al consider the question of sources of heterogeneity in the type I and type III interferon response. Specifically, they are interested in population context in determining heterogeneous responses, as compared to stochastic responses. Using an intestinal epithelial cell line with a live-cell ISG reporter, they observe spatial patterning in the heterogeneity of the IFN response, noting that cells in the center with many neighbors have a lower response than cells on the edge or alone. They show that this pattern is because IFNAR receptors are enriched on the basolateral side of epithelial cells, and high cell density prevents access of IFN to the receptors. They further show this can affect functional responses to infection.

The overall message of this paper is straightforward and the authors convincingly show that receptor expression levels contribute to heterogeneity in the IFN response in this intestinal epithelial cell line. However, the relative impact of the "deterministic" contribution of population context as compared to intrinsic stochasticity in the response is difficult to judge based on the data presented. I agree that culturing conditions can significantly affect results in many experimental systems, and receptor enrichment on one side of epithelial cells has been shown before in closely related contexts (which the authors cite). Overall, the results are interesting, but they are too narrow to claim that population context broadly impacts our understanding of cell-to-cell heterogeneity in the IFN response.

Major comments

1. The review of the literature on cell-to-cell heterogeneity in the introduction should be revised. The authors consider that cell-to-cell heterogeneity in the IFN response could arise from intrinsic stochastic "noise" or from deterministic factors that are determined by population context, and they imply that past studies have concluded it is stochastic. For example, they state ""noise" has been proposed to be critical in cellular decision making and determines, for example, if a cell responds to IFN or not (20, 21, 23, 27)". However, references 20, 21, and 23 are primarily considering heterogeneity in cellular production of type I IFN, not heterogeneity in the response to type 1 IFNs. Rand et al (ref. 20) and Zhao et al (ref 21) address stochasticity of IFNb production following viral infection of fibroblasts and they convincingly show that it is stochastic. Both studies do look briefly at the ISG response and appear to come to different conclusions about its stochasticity (Zhao et al look via imaging and conclude that ISGs are *not* stochastically expressed). However, since both studies use fibroblasts, which should not have a basolateral-apical polarization, these ISG studies cannot be directly compared to the present study without noting that. Schmid et al (ref. 23) looks at a more complex interplay between virus infection, IFN production and ISGs. Maier et al (ref. 27) looks primarily at ISG expression in Huh7 cells and find that it is heterogeneous but not all-or-none. This present study adds to the past literature, but I think it is not justified to imply that past studies "missed" the contribution of population context given that the studies had a different (often broader) focus.

2. The authors conclude at the end of the abstract that "Our data ...[places] population context as a key driver of cell-to-cell

heterogeneity in IFN response." However, this is overstated for multiple reasons. The authors show that population context shifts the mean expression of the Mx1 reporter but they do not plot and compare single-cell distributions. Their images reveal that expression is still very heterogeneous (e.g., Fig. 4B). What if the authors plotted distributions of Mx1 expression for apical and basolateral treatment? Is the variability similar, even if the mean is different, suggesting that intrinsic "stochasticity" is still important for heterogeneous expression?

3. The second weakness is that they have only shown this in one reporter cell line. As mentioned above, studies have made many different conclusions about ISG induction from "not stochastic" to "heterogeneous-but-graded" to "all-or-none". It's likely this is partly to do with the different cell types (fibroblasts vs. epithelial cells), as could also be the case here. To make the claim that population context as a key driver of cell-to-cell heterogeneity, the authors should at least test their findings in 1-2 additional cell lines (perhaps airway epithelial and fibroblasts) using another reporter or a different method of imaging ISG expression. They could also determine if the population-level dependence on culturing density holds in other cell lines/cell types.

Minor comments

1. In the Discussion, the authors state:
"This non-responsiveness of a subpopulation of cells to IFNs is not terminally determined. When the non-responder population is isolated and re-treated with type I IFNs, the same heterogeneous pattern of ISG expression (responder and non-responder cells) was induced, thereby excluding the existence of a stable fraction of unresponsive clones (36, 43). This suggests that within a population, cells actively engage in intercellular communication to reprogram cell subpopulations and generate a precise equilibrium between responsive and non-responsive cells."

The experiment described does not suggest active intercellular communication. There could be many reasons for non-responder populations to respond later, most notably a natural mixing in protein levels as cells divide and changes in relative protein levels due to intracellular circuits, neither of which require active communication.

Reviewer #3:

The authors demonstrate that population-dependent behavior (specifically local cell density, apical-basal polarity, and tight junctions) drive intercellular heterogeneity in IFN responses of human intestinal epithelial cells (IECs). First, when cell density is high enough, they observe that only a fraction of cells at the edge of populations respond to apical IFN stimulation. A micropatterning approach was employed to control population size and density. To investigate spatial distributions of the IFN response, the authors utilize human colon carcinoma T84 cells with a fluorescent protein under transcriptional control of the ISG MX1 promoter, along with high-throughput imaging. Next, distinct aspects of population-dependent behavior are systematically perturbed. Notably, spatial regulation of IFN responses disappears upon basolateral stimulation and when IECs cannot form tight barriers. Finally, cell density is shown to have an important effect on viral responses, as cells seeded at lower densities have lower viral infection rates when they are pre-treated with IFN.

Altogether, the authors present a compelling and interesting argument. Although, extensively studied in bulk, only several recent studies have investigated the cell-to-cell heterogeneity of the IFN response. This study is one of the first to systematically investigate molecular mechanisms underlying the heterogeneity of the IFN response in a high-throughput manner. Additionally, their findings provide an interesting hypothesis for understanding antiviral responses in the gut: intestinal epithelial cells may be designed to selectively sense IFNs provided by immune cells from their basolateral side (although further in vivo validation would be necessary to substantiate this claim).

Before publication, the authors should address several major points regarding extensibility of their findings to non-cancer epithelial cells, quantitative analysis of their spatial heterogeneity results, the duration of the IFN response, and the influence of ZO1-KO on the antiviral immune response.
1. The authors claim in the abstract, introduction, and discussion that their major results are for human epithelial cells. However, they only prove these results using a cancer cell line (T84 human colon carcinoma). To make these claims, the authors must demonstrate their major results are applicable to non-cancer epithelial cells.
2. Figures 2B-C clearly show a spatial heterogeneity in the radial direction. The authors should address this in the text and should also have a plot (either in main text or SI) of the entire distribution of mean fluorescence for edge versus center cells at the single-cell level. If possible, it would be interesting to find a value of fluorescence that separates the edge versus center cells and report what percentage of each population are classified correctly.
3. One question that arises is: do the center cells have a delayed response to stimulation? To address this question in relation to Figures 2B-C, it would be necessary to demonstrate that 24 hours is the maximal antiviral response. The authors should show a later timepoint where the signal is decreased. Also, Figure 3B demonstrates that the maximal response to IFN 1-3 occurs after 24 hours, further motivating the necessity of including a later timepoint.
4. Could the authors comment on why there are significant differences between IFN stimulation of low-density populations upon ZO1-KO (e.g., the difference in ISG fold-change between WT versus ZO1-KO for low-density monolayers)? This potentially indicates that ZO1-KO itself influences the signal transduction of IFNs. The authors should include additional experiments

measuring PSTAT activation for WT versus ZO1-KO cells to determine if ZO1-KO influences IFN activation.

Additionally, several minor points should be addressed:
1. The authors claim that the edge degree is negatively correlated with response to IFN in Figure 1, but they only prove this by performing pairwise p-tests. A better metric to support this claim would be to calculate a correlation coefficient that describes the trend across edge degrees or performing a linear regression across edge degrees and reporting the regression coefficients.
2. Figure 3 has a confusing representation. Figure 3B shows RT-qPCR measurements at 0, 6, 12, 18, and 24 hours but Figure 3A seems to show that only the 24-hour timepoint was collected.
In summary, I believe that this is an important piece of work for the systems biology community and should be published after the modifications outlined above are addressed.

** As a service to authors, EMBO Press offers the possibility to directly transfer declined manuscripts to another EMBO Press title or to the open access journal Life Science Alliance launched in partnership between EMBO Press, Rockefeller University Press and Cold Spring Harbor Laboratory Press. The full manuscript and if applicable, reviewers' reports, are automatically sent to the receiving journal to allow for fast handling and a prompt decision on your manuscript. For more details of this service, and to transfer your manuscript please click on Link Not Available. **

Dear Maria Polychronidou

First, we would like to thank you and the reviewers for this great and constructive review. As mentioned in my Email, we believe that most reviewer comments are easy to address, and we would like to ask if you would consider a revised version addressing the reviewer comments. We have drafted an answer to the reviewers and explain the strategy that we will employ to address their comments below. We apologize as the answer is rather long, but we wanted to make sure that our answers will be easy for you to follow.

As you will see in our response to the individual points, we can easily address all reviewer comments and provide more quantitative, and time resolved data sets that will render the data more compelling. Concerning the lack of models in our experimental set up to transpose our findings *in-vivo* and to challenge their biological significance, we agree with reviewer 1. However, we here insist on the relevance of our findings from a system biology point of view as the outcome of viral infection/host pathogen interactions is extremely dependent on cell density. Importantly, we also would like to highlight that our study provides a novel concept in the field of heterogenous cellular response to interferon where the population context should be considered to explain, at least partially, why not all cells (epithelial cells) respond to IFNs. We will better describe this concept in the revised version.

We are looking forward to hearing from you

All the best

Steeve Boulant

In the name of all co-authors

Reviewer #1:

Metz-Zumaran et al presents analysis to address whether a position of a cell with respect to the neighboring cells affects single cell response to interferons. They report that in populations of intestinal epithelial cells, the cells on the population edge are more responsive than cells within the population. They show that interferon responsiveness and antiviral activity is dependent on cell density. The use of the micropatterning is a nice approach as it provides for a more controlled system for culturing cells in uniform population sizes. However, conclusions are drawn about the spatial impact of interferon responsiveness using cell density experiments without quantitative analysis of the number of center and edge cells within the various density conditions. Additionally, conclusions about interferon responsiveness due to the basolateral location of the IFN receptors have been made without sufficient analysis of the receptors or perturbation studies to address the cellular location of the receptors. Since much of the analysis is lacking high temporal resolution (many of the studies only have 1 or 2 timepoints) and responsiveness occurs at low cell density (a condition that is contrary to the barrier role of intestinal epithelial cells which is also stated in the introduction) it is not clear how relevant or transferable such findings will be for in vivo interferon responses. As such, the conclusions are not compelling.

Major points

(i) Type I and III interferons have different temporal dynamics, so a single timepoint is not sufficient for comparing peak expression between the conditions (Fig 1). PMID: 30485383

We agree that type I and type III IFN response show different dynamics in how they induce expression of ISG. The paper highlighted by the reviewer is actually our very own manuscript. The 24 h treatment in Fig. 1 was chosen as a representative time in which we know for both IFNβ1 and IFNλ1-3 induce high levels of ISG expression. This based on our previous studies (PMID: 30485383) and data in the lab. However, to better address and compare the dynamic response induced by IFNs and the peak expression between both IFNβ1 and IFNλ1-3, we will repeat the experiment with additional timepoints (6, 12, 24, 36, 48 and 72h). From the qPCR data (Fig. 3B) and live cell imaging data (Supp. Fig. 2) showing higher time resolution, we already can strongly speculate that the edge effect is not time sensitive but conserved for all timepoints. This will be fully confirmed with the new time-course experiment in Fig. 1.

(ii) The images used for the analysis of edge vs center cells and edge degree should be shown (Fig 1B) and not just a schematic.

The schematic in Fig. 1B will be changed. Additionally, we will add more representative images (Similar to Fig. 1A) in the supplementary data.

(iii) Figure 1: Only 20% of cells on edge and 25% of singles respond indicating that other factors are more important than edge distance.

We agree that not all edge and single cells respond to IFNλ at 24 h post- treatment (Fig. 1). Previous literature (PMID: 24278020, PMID: 17507495) and data from our lab (PMID: 30485383, PMID: 28484457) supports that type III IFNs induce a lower response as compared to type I IFNs (less magnitude of ISG expression and in less cells). Importantly, we do not claim that cell density and the population context are the sole factor influencing the heterogeneity of IFN response, but rather that it is one parameter among others. This will be clarified in the text to avoid further misunderstandings. Additionally, through the experiment described above as answer to question (i), we speculate that we will induce a higher overall immune response especially in later timepoints, leading to higher percentage of response cells at the edge. In conclusion, we are confident that we will be able to increase the number of edge and single cells responding to IFNλ, although, we foresee that not all cells will be responsive which will be consistent with the literature.

(iv) Need more detail about how cells are grown in these conditions and the impact on cell growth, proliferation, survival, and basal ISG levels using the micropatterning approach. (Fig 2)

When establishing the approach, we characterized the cell populations grown on micropatterns. We will provide this information in the main text.

(v) More quantitative analysis with percentages of edge cells vs center cells at high and low cell densities should be provided. (Fig 3)

Thanks for the suggestions. Indeed, it is important to quantify the percentage of edge and center cells at the different densities to have a better overall understanding of the phenotype. We will use the DBSCAN-CellX App as in Fig. 1 to quantify the number a cells localized at the edge or in the center at both densities.

(vi) Since making claims about cell location (edge vs center), should include images of the cultures seeded at the different cell densities instead of schematics.

Excellent point, representative images of cells at high and low density will be provided.

(vii) The signaling analysis at 1 hour only assumes that different conditions will peak at the same time. This is not a good assumption and multiple time points should be assessed. (Fig 3C)

From previous experiments in our laboratory we observed that pSTAT1 levels are high for both, IFNβ1 and IFNλ1-3, at 1 h post treatment. To rule out possible differences in kinetics between IFNβ1 and IFNλ1-3, we will treat the cells at high and low density and analyze the pSTAT1 levels by WesternBlot in a time dependent manner (time course from 0 to 2 h).

(viii) Should have conditions with only basolateral and only apical stimulation to draw conclusions about the contribution of basolateral stimulation to increase activity of center cells specifically. (Fig 4B,C)

This could be a great proof-of-concept experiment. Unfortunately, it is not possible due to technical limitations. When micropatterning transwells, the cells will grow as populations on restricted (micropatterned) areas of the transwell, while the rest of the transwell membrane will not have any cells on it. At those cell-free areas the reagents can freely diffuse from the apical to the basolateral compartment of the transwell and vice-versa. As a conclusion, it is impossible to treat with IFN from one side only, as it will automatically diffuse to the other side. We will explain this in more detail within the text to avoid further confusion.

(ix) Provide list of proteins from analysis. (Fig 5C)

We will provide a detailed list from the analysis.

(x) Should demonstrate if ZO1 KO affected IFNAR or IFNLR expression levels or trafficking of receptors to apical side? (Fig 6)

This would be indeed a great control, however, the absence of good reagents to immunostaine the IFN receptors makes it difficult to control their location. Although we cannot perform this control, we believe that our data clearly show what is happening in the WT cells with the receptor being polarized on one side of cells, impacting response of epithelial cells to IFN treatment. The use of the ZO-1 KO cells was performed to prove that when IFN is allowed to “diffuse” between epithelial cells and reach the basolateral membrane, cells respond to the cytokines.

(xi) Data presented in Fig 6 does not demonstrate IFN diffusion across cells as apical vs basolateral IFN levels were not measured, but this claim is made: "These results demonstrate that the tight junction protein ZO1 restricts the paracellular diffusion of IFN between polarized T84 cells..."

This is a good point and we indeed made this claim without directly proving it. To address this, we will seed T84 WT and ZO1-KO cells on transwells and allow for a formation of a monolayer. Cells will be treated from the apical side with IFNs and 3 h post-treatment, the medium from the basolateral compartment of the transwell will be harvested. The extend of the paracellular diffusion of the IFNs will be determined by ELISA and/or HEK-blue assay. We anticipate that only in ZO1-KO cells IFNs will be present at the basolateral compartment of the transwell, as the tight junction protein ZO1 will restrict paracellular diffusion in WT cells.

(xii) Linking receptor accessibility to IFN responsiveness is done with limited data (i.e. mass spec with confusing Apical/Basolateral ratios). Could use confocal or other imaging with z-stack and measure known markers for apical vs basolateral location.

This is a good point and ideally we would like to directly visualize the IFN receptor and its polarized localization together with markers for apical and basolateral location. However, after trying many

commercially and non-commercially available antibodies, we failed in specifically staining the IFN receptors. Moreover, we tried to create cell lines expressing the receptor tagged to a fluorescent reporter, however the system was not adequate due to the expression levels. Unfortunately, at this moment it is not possible to visualize the IFN receptors, unless the receptors are strongly overexpressed which will impact their intracellular distribution. However, we will validate our data with another intestinal epithelial cell line CaCo-2 by seeding the cells on transwells in a polarized monolayer as in Fig. 4A, and quantifying the IFN response after apical or basolateral IFN treatment. As a control, we will use non-polarized cells which we know equally respond to "apical and basolateral" IFN treatment.

(xiii) The ratio used for the receptor analysis is not clear: It is named "Apical/Basolateral ratios", but it was calculated based on the apical LFQ signal divided by the total LFQ signal. Why was the basolateral LFQ signal used instead of the total LFQ signal?

This is a good point and it is a labelling error from our side. Indeed, our calculation is based on the apical LFQ signal divided by the total (apical + basolateral) LFQ signal. We labeled the graph wrongly with "Log(2) AP/BL ratio" to make it easier for the reader. However, we agree that it is wrong and will correct the labeling in the Figure.

(xiv) Not sure how relevant some of these questions and experiments are to physiological IFN responses in the intestines since there are no "edge" and "center" cells lining the gut. In the introduction, it is mentioned that the gut is continuous to avoid pathogens invading into other parts of the body.

While under homeostatic conditions the gut is a continuous monolayer of polarized cells, normal peristalsis, pathogen infection and inflammatory flares are known to lead to microlesions and wounds in this epithelial monolayer. Having a differential immune response at these wounds or 'edges' as compared to the intact epithelial might be especially relevant when looking at IBD, which is characterized for dysregulation of immune response. However, we do not want to speculate on this physiological effect within our paper, as we do not have direct evidence for the role of the population context in the intestinal mucosa. The focus of this work lies on cellular and population factors influencing single cell response to IFNs in epithelial cells. Moreover, we want to emphasize the effect that this has on *in vitro* experiments, pointing out that it needs to be considered for drawing conclusions or performing screening assays which we have strongly documented in Figure 7. To address the reviewer's concern, we will be clearer in the text on our system, the aims we want to address, and the relevance for the field.

Minor points

(i) The definition of population context used in this manuscript feels like a narrow view as it doesn't take into account epithelial cell type differences, tissue structure (intestines are uniquely shaped), etc. that are seen in vivo.

This is a good point to address, and we believe that there is a misunderstanding on the concept of population context. We use this term to determine the relation of single cells with respect to other cells. We specifically focus in isogenic cells, meaning that all have the same genomic material and expression profile. Altogether, we want to investigate how the position of a cell in relation to other cells can influence the response to a stimulus and how this could be different/heterogeneous within a population of isogenic cells. We will further emphasize this notion to avoid confusions. Importantly, considering the fact that multiple cell types are present in the gut epithelium is indeed very important, but will complicate the interpretation of the data as we have shown that all intestinal cell

types respond differently to viral challenges and IFN treatment (PMID: 33904651, PMID: 34309190, data not shown).

(ii) There are a few typos throughout the manuscript. Page 5: "downstream 'of' the receptors". Page 10: "population or of IECs seeded".

Thank you, we will correct them.

(iii) Figure 1: What is the percentage of total responders?

We will calculate the percentage of total responders in Fig. 1.

(iv) Figure 3: Were the same densities from the experiment in figure 1 used for this experiment?

No, in Fig. 1 we used medium density to have heterogeneity in the population context, allowing us to determine all factors that could be involved in IFN signaling. For Fig. 3 we use high and low density, which are the 2 extreme conditions in which we see a differential regulation in Fig. 1. We will describe this better in the main text.

(v) Figure 3 legend text should include all times points when RNA was harvested; graphs indicate 0, 6, 12, and 24 hrs.

We will change the schematic from Fig. 3A and the Figure Legend accordingly

(vi) How were the concentrations of interferons used determined? This is especially important when comparing dose-independent gene expression.

The concentrations were chosen from previous publications in our laboratory and previous experiments to reach the maximum responsiveness (saturating concentrations). This will be explained better in the main text.

Reviewer #2:

Metz-Zumaran et al consider the question of sources of heterogeneity in the type I and type III interferon response. Specifically, they are interested in population context in determining heterogeneous responses, as compared to stochastic responses. Using an intestinal epithelial cell line with a live-cell ISG reporter, they observe spatial patterning in the heterogeneity of the IFN response, noting that cells in the center with many neighbors have a lower response than cells on the edge or alone. They show that this pattern is because IFNAR receptors are enriched on the basolateral side of epithelial cells, and high cell density prevents access of IFN to the receptors. They further show this can affect functional responses to infection.

The overall message of this paper is straightforward and the authors convincingly show that receptor expression levels contribute to heterogeneity in the IFN response in this intestinal epithelial cell line. However, the relative impact of the "deterministic" contribution of population context as compared to intrinsic stochasticity in the response is difficult to judge based on the data presented. I agree that culturing conditions can significantly affect results in many experimental systems, and receptor enrichment on one side of epithelial cells has been shown before in closely related contexts (which the authors cite). Overall, the results are interesting, but they are too narrow to claim that population context broadly impacts our understanding of cell-to-cell heterogeneity in the IFN response.

Major comments

1. The review of the literature on cell-to-cell heterogeneity in the introduction should be revised. The authors consider that cell-to-cell heterogeneity in the IFN response could arise from intrinsic stochastic "noise" or from deterministic factors that are determined by population context, and they imply that past studies have concluded it is stochastic. For example, they state ""noise" has been proposed to be critical in cellular decision making and determines, for example, if a cell responds to IFN or not (20, 21, 23, 27)". However, references 20, 21, and 23 are primarily considering heterogeneity in cellular production of type I IFN, not heterogeneity in the response to type 1 IFNs. Rand et al (ref. 20) and Zhao et al (ref 21) address stochasticity of IFNb production following viral infection of fibroblasts and they convincingly show that it is stochastic. Both studies do look briefly at the ISG response and appear to come to different conclusions about its stochasticity (Zhao et al look via imaging and conclude that ISGs are *not* stochastically expressed). However, since both studies use fibroblasts, which should not have a basolateral-apical polarization, these ISG studies cannot be directly compared to the present study without noting that. Schmid et al (ref. 23) looks at a more complex interplay between virus infection, IFN production and ISGs. Maier et al (ref. 27) looks primarily at ISG expression in Huh7 cells and find that it is heterogeneous but not all-or-none. This present study adds to the past literature, but I think it is not justified to imply that past studies "missed" the contribution of population context given that the studies had a different (often broader) focus.

We appreciate a lot this comment and believe that the raised points are valid. We will rephrase the literature review in the introduction accordingly. We will differentiate better between heterogeneity in cellular production and sensing of IFNs. Moreover, we will emphasize on the cellular system used in previous publications, for example whether they use cells that are polarized or not, and whether it is comparable to our study on cell-to-cell heterogeneity in epithelial cells that have the ability to polarize. Finally, we want to point out the importance of the stochastic events for cell-to-cell

variability, and not imply that the population context has been understudied but rather that our study is a contribution to better understand the origin of heterogeneity in epithelial cells.

2. The authors conclude at the end of the abstract that "Our data ...[places] population context as a key driver of cell-to-cell heterogeneity in IFN response." However, this is overstated for multiple reasons. The authors show that population context shifts the mean expression of the Mx1 reporter but they do not plot and compare single-cell distributions. Their images reveal that expression is still very heterogeneous (e.g., Fig. 4B). What if the authors plotted distributions of Mx1 expression for apical and basolateral treatment? Is the variability similar, even if the mean is different, suggesting that intrinsic "stochasticity" is still important for heterogeneous expression?

We agree that we may have made an over-statement. We do not attribute the population context as the most important origin for cell-to-cell heterogeneity during IFN response. We rather state that the population context is a deterministic factor that should be considered when analyzing cell-to-cell variability. However, we understand that we failed in making that point completely clear throughout the text and will rephrase it accordingly. We will emphasize the role of stochastic events for single cell heterogeneity during immune response. Especially when considering the reviewer's observation, that the prom-Mx1-mCherry expression is still very heterogeneous and that we did not compare single-cell distributions.

3. The second weakness is that they have only shown this in one reporter cell line. As mentioned above, studies have made many different conclusions about ISG induction from "not stochastic" to "heterogeneous-but-graded" to "all-or-none". It's likely this is partly to do with the different cell types (fibroblasts vs. epithelial cells), as could also be the case here. To make the claim that population context as a key driver of cell-to-cell heterogeneity, the authors should at least test their findings in 1-2 additional cell lines (perhaps airway epithelial and fibroblasts) using another reporter or a different method of imaging ISG expression. They could also determine if the population-level dependence on culturing density holds in other cell lines/cell types.

We agree that drawing our conclusions from only on cell type (intestinal epithelial cell line T84) can be not sufficient, and that using other cell lines will make conclusions stronger and improve this study. We will address this point as follows:

- We will validate our findings in other epithelial cell lines:
    - CaCo2: intestinal epithelial cell lines
    - Calu3: airway epithelial cell line
- We will test whether the population context affects cell types which do not polarize. To this end we will perform the experiments in at least one fibroblast cell line. As we already tested in out laboratory that HK2 cells are fully responsive to type I and type III IFNs, we will start using that cell line. In parallel we will test responsiveness to both IFNs in other fibroblastic cell lines in our lab, and eventually perform the experiments in those as well.
- As we do not have the fluorescent reporter in those cell lines, we will perform other experiments to validate our results:
    - To address the spatial heterogeneity during IFN response in a visual manner, cells will be seeded at medium confluence, treated with IFN and then fixed. Cells will then be immunostained again ISGs to assess which cells are responsive to IFNs.
    - Cells will be seeded at high and low density as established before, and treated from the apical side with IFNs. Expression of ISG will be assessed by qRT-PCR.
    - For the cell lines that polarize (CaCo2 and Calu3), we will seed them on transwells and allow for formation of a polarized monolayer as determined by TEER

measurement. Then cells will be treated from the apical or basolateral side with IFNs, and the ISG expression will be measured by qRT-PCR as in Fig. 4A.

Minor comments

1. In the Discussion, the authors state:

"This non-responsiveness of a subpopulation of cells to IFNs is not terminally determined. When the non-responder population is isolated and re-treated with type I IFNs, the same heterogeneous pattern of ISG expression (responder and non-responder cells) was induced, thereby excluding the existence of a stable fraction of unresponsive clones (36, 43). This suggests that within a population, cells actively engage in intercellular communication to reprogram cell subpopulations and generate a precise equilibrium between responsive and non-responsive cells."

The experiment described does not suggest active intercellular communication. There could be many reasons for non-responder populations to respond later, most notably a natural mixing in protein levels as cells divide and changes in relative protein levels due to intracellular circuits, neither of which require active communication.

We agree with the comment and wrongly interpreted the studies. We will change the text accordingly.

Reviewer #3:

The authors demonstrate that population-dependent behavior (specifically local cell density, apical-basal polarity, and tight junctions) drive intercellular heterogeneity in IFN responses of human intestinal epithelial cells (IECs). First, when cell density is high enough, they observe that only a fraction of cells at the edge of populations respond to apical IFN stimulation. A micropatterning approach was employed to control population size and density. To investigate spatial distributions of the IFN response, the authors utilize human colon carcinoma T84 cells with a fluorescent protein under transcriptional control of the ISG MX1 promoter, along with high-throughput imaging. Next, distinct aspects of population-dependent behavior are systematically perturbed. Notably, spatial regulation of IFN responses disappears upon basolateral stimulation and when IECs cannot form tight barriers. Finally, cell density is shown to have an important effect on viral responses, as cells seeded at lower densities have lower viral infection rates when they are pre-treated with IFN.

Altogether, the authors present a compelling and interesting argument. Although, extensively studied in bulk, only several recent studies have investigated the cell-to-cell heterogeneity of the IFN response. This study is one of the first to systematically investigate molecular mechanisms underlying the heterogeneity of the IFN response in a high-throughput manner. Additionally, their findings provide an interesting hypothesis for understanding antiviral responses in the gut: intestinal epithelial cells may be designed to selectively sense IFNs provided by immune cells from their basolateral side (although further in vivo validation would be necessary to substantiate this claim).

Before publication, the authors should address several major points regarding extensibility of their findings to non-cancer epithelial cells, quantitative analysis of their spatial heterogeneity results, the duration of the IFN response, and the influence of ZO1-KO on the antiviral immune response.

1. The authors claim in the abstract, introduction, and discussion that their major results are for human epithelial cells. However, they only prove these results using a cancer cell line (T84 human colon carcinoma). To make these claims, the authors must demonstrate their major results are applicable to non-cancer epithelial cells.

This is a very good point and we will perform key experiments in human ileum and/or colon-derived organoids. We will perform all (or at least one) of the following experiments:

- Population context and cell-to-cell heterogeneity: Ileum and/or colon-derived organoids will be seeded at medium density on 8-well ibidi chambers (glass bottom). After differentiation, cells will be treated from the apical side (basolateral side is attached to glass) with type I and type III IFNs. 24 h post-treatment, cells will be fixed and immunostained for ISGs. We will determine which cells are responsive to IFNs with respect to their position within the population context by quantifying the spatial distribution ISG-expressing cells, allowing to confirm the impact of the population context in cell-to-cell heterogeneity in a physiological primary epithelial cell model system.
- IFN treatment of organoids from the apical or basolateral side: Ileum and/or colon-derived organoids will be seeded on transwells (as in Fig. 4A) allowing for formation of a monolayer of polarized (as determined by TEER measurement) and differentiated (as determined by RT-q-PCR) cells through air-liquid interphase. The polarized monolayer of cells will then be treated from the apical or basolateral side with type I and type III IFNs, and the ISG induction will be determined by q-RT-PCR. This will show whether in a more physiological model intestinal epithelial cells are more responsive from the basolateral side, thereby confirming an asymmetric polarized response to IFNs.

- IFN treatment of classical and inside-out 3D organoids: Organoids will be grown as 3D structures in matrigel. Classically, the organoids grow with the apical side facing inside the structure and the basolateral side facing the matrigel (IFN treatment side). Additionally, we will generate inside-out organoids, in which the basolateral side will face inwards while the apical side will face the matrigel and be accessible for IFN treatment. Treatment of classical or inside-out 3D organoids will allow for IFN interaction with solely the basolateral or apical side, respectively. Measurement of ISG expression by q-RT-PCR will support whether response to IFN is polarized in 3D organoids, which resemble a physiological crypt-villi axis.

2. Figures 2B-C clearly show a spatial heterogeneity in the radial direction. The authors should address this in the text and should also have a plot (either in main text or SI) of the entire distribution of mean fluorescence for edge versus center cells at the single-cell level. If possible, it would be interesting to find a value of fluorescence that separates the edge versus center cells and report what percentage of each population are classified correctly.

This is a good observation. We will address the radial direction of spatial heterogeneity in the text and plot the distribution of mean fluorescence for edge vs. center at the single-cell level.

3. One question that arises is: do the center cells have a delayed response to stimulation? To address this question in relation to Figures 2B-C, it would be necessary to demonstrate that 24 hours is the maximal antiviral response. The authors should show a later timepoint where the signal is decreased. Also, Figure 3B demonstrates that the maximal response to IFN1-3 occurs after 24 hours, further motivating the necessity of including a later timepoint.

This is good point and we already have performed experiments showing that it is not the case (the data were not shown). In the revised version, we will address it by measuring IFN responsiveness at later timepoints for several experiments:

- We will quantify the spatial heterogeneity in prom-Mx1-mCherry expression as in Fig. 1 (see answer to Reviewer 1, comment (i)).
- We will follow the expression of prom-Mx1-mCherry in cell population seeded on patterns for 36, 48 and 72 h post IFN treatment (experimental setup as in Fig. 2A).
- We will measure at the endogenous ISG expression with q-RT-PCR at high vs. low density at 36, 48 and 72 h post IFN treatment (experimental setup as Figure 3A-B).

4. Could the authors comment on why there are significant differences between IFN stimulation of low-density populations upon ZO1-KO (e.g., the difference in ISG fold-change between WT versus ZO1-KO for low-density monolayers)? This potentially indicates that ZO1-KO itself influences the signal transduction of IFNs. The authors should include additional experiments measuring PSTAT activation for WT versus ZO1-KO cells to determine if ZO1-KO influences IFN activation.

Preliminary data in our laboratory revealed that cell density (as sensed by junctional complexes in which ZO1 is present) influences IFN signaling. We are currently investigating the molecular mechanism linking potentially ZO-1 and tight junction to IFN signaling. Like the reviewer, we believe that it is very interesting but we also think that this does not belong to the scope of this manuscript. However, we will comment on the difference in IFN signaling between ZO1-KO and WT cells in the main text of this manuscript and provide pSTAT activation kinetics as asked by the reviewer.

Additionally, several minor points should be addressed:

1. The authors claim that the edge degree is negatively correlated with response to IFN in Figure 1, but they only prove this by performing pairwise p-tests. A better metric to support this claim would

be to calculate a correlation coefficient that describes the trend across edge degrees or performing a linear regression across edge degrees and reporting the regression coefficients.

This is a very good suggestion, and we will plot a linear regression and calculate the correlation coefficient.

2. Figure 3 has a confusing representation. Figure 3B shows RT-qPCR measurements at 0, 6, 12, 18, and 24 hours but Figure 3A seems to show that only the 24-hour timepoint was collected.

Thanks for pointing it out, we will update the schematic.

In summary, I believe that this is an important piece of work for the systems biology community and should be published after the modifications outlined above are addressed.

26th Jul 2023

Manuscript Number: MSB-2023-11778R-Q
Title: Population context drives cell-to-cell variability in interferon response in epithelial cells

Dear Steeve,

Thank you for your message regarding our decision on your manuscript MSB-2023-11778. I have now had the chance to read the manuscript and your point-by-point response to the reviewers' comments and I have discussed them with the team. As I will explain below, we would not be opposed to considering a revised and extended manuscript addressing the issues raised by the reviewers.

During the review of your study, the reviewers appreciated that the presented findings seem potentially interesting. However, they indicated that the main conclusions were not well supported and the broader relevance was somewhat unclear. We think that the proposed additional experimental analyses (including performing measurements at additional time points and cell lines and performing further quantifications) as well as the clarifications regarding the broader relevance of the main findings seem promising for addressing the reviewers' concerns and for increasing the conclusiveness of the study. Regarding the limitations related to performing certain analyses suggested by the reviewers (e.g. visualizing the IFN receptors) we think that it is very important to describe in detail in your point by point response why these analyses cannot be performed.

We would therefore invite you to submit a revised and extended manuscript, which will be sent back to the reviewers so that they can assess if their concerns have been satisfactorily addressed. Given that the revised manuscript will be subjected to peer review and that addressing the issues raised involves several analyses with unclear outcome, as you can probably understand we can give no guarantee about the eventual acceptability of the study. If you do decide to resubmit the extended/revised manuscript, we would ask you to enclose with your resubmission a point-by-point response to the points raised.

Let me know in case you have any questions or if there is anything you would like to discuss with me later on.

Kind regards,

Maria

Maria Polychronidou, PhD
Senior Editor
Molecular Systems Biology

Use the link below to submit your revision:

https://msb.msubmit.net/cgi-bin/main.plex

*** PLEASE NOTE *** As part of the EMBO Press transparent editorial process initiative (see our Editorial at https://dx.doi.org/10.1038/msb.2010.72), Molecular Systems Biology publishes online a Review Process File with each accepted manuscripts. This file will be published in conjunction with your paper and will include the anonymous referee reports, your point-by-point response and all pertinent correspondence relating to the manuscript. If you do NOT want this File to be published, please inform the editorial office at msb@embo.org within 14 days upon receipt of the present letter.

Dear Maria Polychronidou and reviewers

We thank you for the opportunity to resubmit our manuscript entitled "Population context drives cell-to-cell variability in interferon response in epithelial cells".

Please find below our answers to the reviewer comments. (Reviewer comments are in black and our answers are in blue).

Best

Steeve Boulant, on behalf of all co-authors

Reviewer #1:

Metz-Zumaran et al presents analysis to address whether a position of a cell with respect to the neighboring cells affects single cell response to interferons. They report that in populations of intestinal epithelial cells, the cells on the population edge are more responsive than cells within the population. They show that interferon responsiveness and antiviral activity is dependent on cell density. The use of the micropatterning is a nice approach as it provides for a more controlled system for culturing cells in uniform population sizes. However, conclusions are drawn about the spatial impact of interferon responsiveness using cell density experiments without quantitative analysis of the number of center and edge cells within the various density conditions. Additionally, conclusions about interferon responsiveness due to the basolateral location of the IFN receptors have been made without sufficient analysis of the receptors or perturbation studies to address the cellular location of the receptors. Since much of the analysis is lacking high temporal resolution (many of the studies only have 1 or 2 timepoints) and responsiveness occurs at low cell density (a condition that is contrary to the barrier role of intestinal epithelial cells which is also stated in the introduction) it is not clear how relevant or transferable such findings will be for in vivo interferon responses. As such, the conclusions are not compelling.

Major points

(i) Type I and III interferons have different temporal dynamics, so a single timepoint is not sufficient for comparing peak expression between the conditions (Fig 1). PMID: 30485383

We agree that type I and type III IFN response show different dynamics in how they induce expression of ISG. The paper highlighted by the reviewer is our own manuscript. The 24 h treatment in Fig. 1 was chosen as a representative time in which we know for both IFNβ1 and IFNλ1-3 induce high levels of ISG expression. This was based on our previous studies (PMID: 30485383) and data in the lab. However, to better address and compare the dynamic response induced by IFNs and the peak expression of ISGs between both IFNβ1 and IFNλ1-3, we repeated the experiment with additional timepoints (6, 12, 24, 36, 48, 72 and 96 h). Results can be found in Fig. 1F, and we demonstrate that the edge effect is not time dependent but is conserved for all timepoints. We also chose similar timepoints when analyzing the response of cells seeded as populations on micropatterns (Fig. 2D). Similar to Fig. 1F, we found that the edge cells are more responsive than the center cell, regardless of the time post IFN treatment (Fig. 2D).

(ii) The images used for the analysis of edge vs center cells and edge degree should be shown (Fig 1B) and not just a schematic.

Representative images used for the analysis of edge vs. center cells and the edge degree are depicted in Fig. 1A. We changed the schematic in Fig. 1B and hope it is clearer.

(iii) Figure 1: Only 20% of cells on edge and 25% of singles respond indicating that other factors are more important than edge distance.

We agree that not all edge and single cells respond to IFNλ at 24 h post- treatment (Fig. 1). Previous literature (PMID: 24278020, PMID: 17507495) and data from our lab (PMID: 30485383, PMID: 28484457) supports that type III IFNs induce a lower response as compared to type I IFNs (less magnitude of ISG expression and in less cells). Importantly, we have modified the manuscript to make sure that we did not claim that cell density and the population context are the sole factor influencing the heterogeneity of IFN response, but rather that it is one parameter among others. This is now clarified in the text to avoid misunderstandings.

(iv) Need more detail about how cells are grown in these conditions and the impact on cell growth, proliferation, survival, and basal ISG levels using the micropatterning approach. (Fig 2)

We performed a characterization of key molecular features of cell populations grown on micropatterns. This can be found in Supp. Fig. 2 and in the results section.

(v) More quantitative analysis with percentages of edge cells vs center cells at high and low cell densities should be provided. (Fig 3)

Thanks for the suggestions. Indeed, it is important to quantify the percentage of edge and center cells at the different densities to have a better overall understanding of the phenotype. We used the DBSCAN-CellX App as in Fig. 1 to quantify the number a cells localized at the edge or in the center at both densities. These results can be found in Supp. Fig. 3B and are described in the results section.

(vi) Since making claims about cell location (edge vs center), should include images of the cultures seeded at the different cell densities instead of schematics.

Representative images of cells at high and low density are provided in Supp. Fig. 3A.

(vii) The signaling analysis at 1 hour only assumes that different conditions will peak at the same time. This is not a good assumption and multiple time points should be assessed. (Fig 3C)

To rule out possible differences in kinetics between IFNβ1 and IFNλ1-3, we treated the cells at high and low density and analyzed the pSTAT1 levels by WesternBlot in a time dependent manner (time course from 0 to 2 h). Results are shown in Fig. 3C and are described in the results section. Our conclusions remained unchanged with these kinetics data.

(viii) Should have conditions with only basolateral and only apical stimulation to draw conclusions about the contribution of basolateral stimulation to increase activity of center cells specifically. (Fig 4B,C)

This could be a great proof-of-concept experiment. Unfortunately, it is not possible due to technical limitations. When micropatterning transwells, the cells will grow as populations on restricted (micropatterned) areas of the transwell, while the rest of the transwell will not have any cells on it. At those cell-free areas, the reagents can freely diffuse from the apical to the basolateral compartment of the transwell and vice-versa. As a conclusion, it is impossible to treat with IFN from one side only, as it will automatically diffuse to the other side. We explained this in more detail within the text to avoid further confusion.

(ix) Provide list of proteins from analysis. (Fig 5C)

We are providing the list of proteins from the analysis as an Excel sheet.

(x) Should demonstrate if ZO1 KO affected IFNAR or IFNLR expression levels or trafficking of receptors to apical side? (Fig 6)

This would be indeed a great control, however, the absence of good reagents to immunostain the IFN receptors makes it difficult to control their location. Similarly, our mass spectrometry approach cannot be employed with ZO1 KO cells as the side specific biotinylation approach relies on formations of tight barrier blocking paracellular diffusion. In ZO-1 KO cells, this paracellular diffusion is impaired (Fig. 6D-E) and as such we cannot specifically biotinylate the apical or basolateral side of our cells. Although we cannot perform this control, we believe that our data clearly show that in polarized T84 WT cells the IFN receptors are enriched at the basolateral membrane, thereby impacting response of epithelial cells to IFN treatment. The use of the ZO-1 KO cells was performed to prove that, when IFN is allowed to "diffuse" between epithelial cells and reach the basolateral membrane, cells respond to the cytokines.

(xi) Data presented in Fig 6 does not demonstrate IFN diffusion across cells as apical vs basolateral IFN levels were not measured, but this claim is made: "These results demonstrate that the tight junction protein ZO1 restricts the paracellular diffusion of IFN between polarized T84 cells..."

To address this, we seeded T84 WT and ZO1-KO cells on transwells and allowed for the formation of a monolayer. Cells were then treated from the apical side with IFNs and 3 h post-treatment, the medium from the basolateral compartment of the transwell was harvested. The extend of the paracellular diffusion of the IFNs was determined by HEK-blue assay. The results demonstrated that only in ZO1-KO cells, IFNs are present at the basolateral compartment of the transwell. This demonstrates that the tight junction protein ZO1 restricts paracellular IFN diffusion in WT cells. The results are shown in Fig. 6E and are described in the results section.

(xii) Linking receptor accessibility to IFN responsiveness is done with limited data (i.e. mass spec with confusing Apical/Basolateral ratios). Could use confocal or other imaging with z-stack and measure known markers for apical vs basolateral location.

This is a good point and ideally we would like to directly visualize the IFN receptor and its polarized localization together with markers for apical and basolateral location. However, after trying many commercially and non-commercially available antibodies, we failed in specifically staining the IFN receptors. Moreover, we tried to create cell lines expressing the receptor tagged to a fluorescent reporter, however this system was not adequate due to the high overexpression levels. Unfortunately, at this moment it is not possible to visualize the IFN receptors, unless the receptors are strongly overexpressed which will impact their intracellular distribution.

However, we validated our data with the airway epithelial cell line Calu3 (see Fig. 4C) and primary human ileum-derived organoids (see Fig. 4D) by seeding the cells on transwells as a polarized monolayer as in Fig. 4A. We quantified the IFN response after apical or basolateral IFN treatment (Fig. 4C-D). Moreover, we confirmed that primary human-derived organoids are more responsive from basolateral IFN treatment as compared to apical treatment by using a novel apical-out and basolateral-out 3D culturing system (Fig. 4E-G). Finally, to control that this phenotype is truly due to a basolateral localization of the receptor, we used cells that do not polarize along the apical-basolateral axis and therefore should not have a polarized IFN receptor polarization. Indeed, out results show that the non-epithelial Swiss 3T3 did not show any differential immune response as high vs. low density (Supp. Fig. 5B).

(xiii) The ratio used for the receptor analysis is not clear: It is named "Apical/Basolateral ratios", but it was calculated based on the apical LFQ signal divided by the total LFQ signal. Why was the basolateral LFQ signal used instead of the total LFQ signal?

We have clarified this issue in the text. Our calculation is based on the comparison of the LFQ signal between apical and basolateral biotinylation samples, and their LFQ log(2) ratio was used together with a pairwise t-test and sample shuffling-FDR to assign the proteins to either side of the generated volcano plot and evaluate the significance of the enrichment. We corrected the mistake in the material and methods section.

(xiv) Not sure how relevant some of these questions and experiments are to physiological IFN responses in the intestines since there are no "edge" and "center" cells lining the gut. In the introduction, it is mentioned that the gut is continuous to avoid pathogens invading into other parts of the body.

While under homeostatic conditions the gut is a continuous monolayer of polarized cells, normal peristalsis, pathogen infection and inflammatory flares are known to lead to microlesions and wounds in this epithelial monolayer (PMID: 32572429, PMID: 31191468, PMID: 35695206). Having a differential immune response at these wounds or ‘edges’ as compared to the intact epithelial might be especially relevant when looking at IBD, which is characterized for dysregulation of immune response. However, we do not want to speculate on this physiological effect within our paper, as we do not have direct evidence for the role of the population context in the intestinal mucosa. The focus of this work lies on cellular and population factors influencing single cell response to IFNs in epithelial cells. Moreover, we want to emphasize the effect that this has on *in vitro* experiments, pointing out that it needs to be considered for drawing conclusions or performing screening assays which we have strongly documented in Fig. 7. To address the reviewer’s concern, we better described in the text the purpose of the study and its relevance for the field.

Finally, we demonstrated in Fig 4D-G and Supp. Fig. 1D that human ileum-derived organoids show the same phenotype as epithelial cell lines (edge cells being more responsive), and are more responsive from the basolateral membrane to IFN treatment. Organoid systems simulate physiological intestinal epithelium conditions. Therefore, we would argue that in the intestinal epithelium the IFN receptors are most likely also localized to the basolateral side of cells. In the discussion we shortly describe the possible physiological impact that this could have: “*A polarized receptor localization might have a physiological relevance, since in-vivo IECs are in contact with the lamina propria from the basolateral side where immune cells are also situated. On the contrary, the apical membrane faces the gut lumen containing the commensal microbiota system. Sensing IFNs from the sterile basolateral side could be a mechanism to selectively sense IFNs provided by immune cells. IECs also express and secrete IFNs to act in an autocrine and paracrine manner, and to propagate an antiviral immune response. Interestingly and in line with our results, it was demonstrated that after virus infection of polarized hIECs in-vitro, IFNλ was secreted predominantly to the basolateral side (PMID: 27279006). Further studies must address whether IFN secretion in-vivo by IECs occurs on the apical or basolateral side, and how this is relevant in the context of a basolateral IFN receptor localization.*”

Minor points

(i) The definition of population context used in this manuscript feels like a narrow view as it doesn't take into account epithelial cell type differences, tissue structure (intestines are uniquely shaped), etc. that are seen in vivo.

This is a good point to address, and we believe that there is a misunderstanding on the concept of population context. We apologize for this. We use this term to determine the relation of single cells with respect to other cells. We specifically focus on isogenic cell groups, meaning that all have the same genomic material and expression profile. Altogether, we want to

investigate how the position of a cell in relation to other cells can influence the response to a stimulus and how this could be different/heterogeneous within a population of isogenic cells.

Importantly, considering the fact that multiple cell types are present in the gut epithelium is indeed very important, but will complicate the interpretation of the data as we have shown that all intestinal cell types respond differently to viral challenges and IFN treatment (PMID: 33904651, PMID: 34309190).

(ii) There are a few typos throughout the manuscript. Page 5: "downstream 'of' the receptors". Page 10: "population or of IECs seeded".

Thank you, we have corrected them.

(iii) Figure 1: What is the percentage of total responders?

We calculated the percentage of total responders for a time period of 96 hours (experimental setup as in Fig. 1). The results are shown in Supp. Fig. 1C and are explained in the text.

(iv) Figure 3: Were the same densities from the experiment in figure 1 used for this experiment?

No, in Fig. 1 we used medium density to have heterogeneity in the population context, allowing us to determine all factors that could be involved in IFN signaling. For Fig. 3 we use high and low density, which are the 2 extreme conditions in which we see a differential regulation in Fig. 1. These points are now better described in the text.

(v) Figure 3 legend text should include all times points when RNA was harvested; graphs indicate 0, 6, 12, and 24 hrs.

Thank you for pointing that out. We changed the schematic from Fig. 3A and the Figure Legend accordingly.

(vi) How were the concentrations of interferons used determined? This is especially important when comparing dose-independent gene expression.

The concentrations were chosen from previous publications in our laboratory and previous experiments to reach the maximum responsiveness (saturating concentrations). This is now explained in the main text.

Reviewer #2:

Metz-Zumaran et al consider the question of sources of heterogeneity in the type I and type III interferon response. Specifically, they are interested in population context in determining heterogeneous responses, as compared to stochastic responses. Using an intestinal epithelial cell line with a live-cell ISG reporter, they observe spatial patterning in the heterogeneity of the IFN response, noting that cells in the center with many neighbors have a lower response than cells on the edge or alone. They show that this pattern is because IFNAR receptors are enriched on the basolateral side of epithelial cells, and high cell density prevents access of IFN to the receptors. They further show this can affect functional responses to infection.

The overall message of this paper is straightforward and the authors convincingly show that receptor expression levels contribute to heterogeneity in the IFN response in this intestinal epithelial cell line. However, the relative impact of the "deterministic" contribution of population context as compared to intrinsic stochasticity in the response is difficult to judge based on the data presented. I agree that culturing conditions can significantly affect results in many experimental systems, and receptor enrichment on one side of epithelial cells has been shown before in closely related contexts (which the authors cite). Overall, the results are interesting, but they are too narrow to claim that population context broadly impacts our understanding of cell-to-cell heterogeneity in the IFN response.

Major comments

1. The review of the literature on cell-to-cell heterogeneity in the introduction should be revised. The authors consider that cell-to-cell heterogeneity in the IFN response could arise from intrinsic stochastic "noise" or from deterministic factors that are determined by population context, and they imply that past studies have concluded it is stochastic. For example, they state ""noise" has been proposed to be critical in cellular decision making and determines, for example, if a cell responds to IFN or not (20, 21, 23, 27)". However, references 20, 21, and 23 are primarily considering heterogeneity in cellular production of type I IFN, not heterogeneity in the response to type 1 IFNs. Rand et al (ref. 20) and Zhao et al (ref 21) address stochasticity of IFNb production following viral infection of fibroblasts and they convincingly show that it is stochastic. Both studies do look briefly at the ISG response and appear to come to different conclusions about its stochasticity (Zhao et al look via imaging and conclude that ISGs are *not* stochastically expressed). However, since both studies use fibroblasts, which should not have a basolateral-apical polarization, these ISG studies cannot be directly compared to the present study without noting that. Schmid et al (ref. 23) looks at a more complex interplay between virus infection, IFN production and ISGs. Maier et al (ref. 27) looks primarily at ISG expression in Huh7 cells and find that it is heterogeneous but not all-or-none. This present study adds to the past literature, but I think it is not justified to imply that past studies "missed" the contribution of population context given that the studies had a different (often broader) focus.

We acknowledge the comments made by the reviewer. We have now rephrased the literature review in the introduction. The following major changes were made:

(a) The literature reviewed in the introduction is only about heterogeneity during IFN sensing, since this is also the focus of the study. The heterogeneity in cellular IFN production is not addressed anymore, as it is not relevant for this study.
(b) We specified the cellular system used in previous publications, and outlined their origin (epithelial tissues or not). This allows us to address in the discussion whether previous results are comparable to our study on cell-to-cell heterogeneity, especially focusing on the fact that these cells polarize or not along the apical-basolateral axis.

(c) Finally, we pointed out the importance of the stochastic events for cell-to-cell variability during IFN signaling. Moreover, in the last paragraph of the Introduction we say that the population context is one factor influencing cell-to-cell variability, but only in epithelial cells characterized by polarization along the apical-basolateral axis.

Additionally, we have adjusted our discussion, pointing out that in previous studies different cell models were used, and that stochastic events are pivotal factors contributing to cell-to-cell variability during IFN signaling. Moreover, we have moderated our conclusions, and not imply that the population context has been understudied, but rather that our study is a contribution to better understand the origin of heterogeneity in epithelial cells.

2. The authors conclude at the end of the abstract that "Our data ...[places] population context as a key driver of cell-to-cell heterogeneity in IFN response." However, this is overstated for multiple reasons. The authors show that population context shifts the mean expression of the Mx1 reporter but they do not plot and compare single-cell distributions. Their images reveal that expression is still very heterogeneous (e.g., Fig. 4B). What if the authors plotted distributions of Mx1 expression for apical and basolateral treatment? Is the variability similar, even if the mean is different, suggesting that intrinsic "stochasticity" is still important for heterogeneous expression?

We agree that we have made an over-statement. We do not attribute the population context as the most important origin for cell-to-cell heterogeneity during IFN response in adherent epithelial cells. We rather state that the population context is a deterministic factor that should be considered when analyzing cell-to-cell variability. However, we understand that we failed in making that point completely clear throughout the text. We have now rephrased it accordingly in the abstract, the introduction and the discussion. We furthermore emphasize on the role of stochastic events for single cell heterogeneity during IFN-signaling. Especially when considering the reviewer's observation, that the prom-Mx1-mCherry expression is still very heterogeneous and that we did not compare single-cell distributions.

3. The second weakness is that they have only shown this in one reporter cell line. As mentioned above, studies have made many different conclusions about ISG induction from "not stochastic" to "heterogeneous-but-graded" to "all-or-none". It's likely this is partly to do with the different cell types (fibroblasts vs. epithelial cells), as could also be the case here. To make the claim that population context as a key driver of cell-to-cell heterogeneity, the authors should at least test their findings in 1-2 additional cell lines (perhaps airway epithelial and fibroblasts) using another reporter or a different method of imaging ISG expression. They could also determine if the population-level dependence on culturing density holds in other cell lines/cell types.

We agree that drawing our conclusions from only on cell type (intestinal epithelial cell line T84) can be not sufficient, and that using other cell lines will make conclusions stronger and improve this study. We addressed this point as follows:

- We confirmed our findings in other epithelial cell lines (Fig. 4C, Supp. Fig. 5A):
  - CaCo2: intestinal epithelial cell lines
  - Calu3: airway epithelial cell line
  - HK2: human kidney tubule epithelial cell
  - Huh7: human hepatocyte derived cellular carcinoma, epithelial-like → this cell line has been used by another study cited in the introduction (*Maier et al., PLOS, 2022*)
- We confirmed our findings in primary human ileum-derived organoids (epithelial cell) (Fig. 4D-G).

- We tested how the population context, specifically cell density, affects cell types which cannot not polarize along the apical-basolateral axis (Supp. Fig. 5B):
    - 3T3-Swiss albino: mouse fibroblast cell line → has been used by another study cited in the introduction (*Rand et al., MSB, 2012*)
- We used different methods to challenge our hypothesis. Note: not all methods were used for all cell types. Following were the methods:
    - Measurement of the ISG expression by RT-q-PCR after apical IFN treatment of cells seeded at high and low density.
    - Seeding cells on transwells until formation of a polarized monolayer as determined by TEER measurement. Apical or basolateral treatment with IFNs, and quantification of the induction of ISG expression.
    - Seeding of cell on a glass surface in 2D and apical treatment with IFNs. Assessing the spatial localization of responder cells by immunostaining of ISG15.
    - Growing 3D ileum-derived organoids in an "apical-out" and "basolateral-out" conformation, to test under which conditions they are more responsive to IFN treated (quantify ISG expression by RT-q-PCR).

Together our results demonstrate that the population context and the basolateral receptor localization affects cell-to-cell responsiveness to IFN treatment in epithelial cells.

Minor comments

1. In the Discussion, the authors state:

"This non-responsiveness of a subpopulation of cells to IFNs is not terminally determined. When the non-responder population is isolated and re-treated with type I IFNs, the same heterogeneous pattern of ISG expression (responder and non-responder cells) was induced, thereby excluding the existence of a stable fraction of unresponsive clones (36, 43). This suggests that within a population, cells actively engage in intercellular communication to reprogram cell subpopulations and generate a precise equilibrium between responsive and non-responsive cells."

The experiment described does not suggest active intercellular communication. There could be many reasons for non-responder populations to respond later, most notably a natural mixing in protein levels as cells divide and changes in relative protein levels due to intracellular circuits, neither of which require active communication.

We agree with the comment and wrongly interpreted the studies. We changed the text accordingly.

Reviewer #3:

The authors demonstrate that population-dependent behavior (specifically local cell density, apical-basal polarity, and tight junctions) drive intercellular heterogeneity in IFN responses of human intestinal epithelial cells (IECs). First, when cell density is high enough, they observe that only a fraction of cells at the edge of populations respond to apical IFN stimulation. A micropatterning approach was employed to control population size and density. To investigate spatial distributions of the IFN response, the authors utilize human colon carcinoma T84 cells with a fluorescent protein under transcriptional control of the ISG MX1 promoter, along with high-throughput imaging. Next, distinct aspects of population-dependent behavior are systematically perturbed. Notably, spatial regulation of IFN responses disappears upon basolateral stimulation and when IECs cannot form tight barriers. Finally, cell density is shown to have an important effect on viral responses, as cells seeded at lower densities have lower viral infection rates when they are pre-treated with IFN.

Altogether, the authors present a compelling and interesting argument. Although, extensively studied in bulk, only several recent studies have investigated the cell-to-cell heterogeneity of the IFN response. This study is one of the first to systematically investigate molecular mechanisms underlying the heterogeneity of the IFN response in a high-throughput manner. Additionally, their findings provide an interesting hypothesis for understanding antiviral responses in the gut: intestinal epithelial cells may be designed to selectively sense IFNs provided by immune cells from their basolateral side (although further in vivo validation would be necessary to substantiate this claim).

Before publication, the authors should address several major points regarding extensibility of their findings to non-cancer epithelial cells, quantitative analysis of their spatial heterogeneity results, the duration of the IFN response, and the influence of ZO1-KO on the antiviral immune response.

1. The authors claim in the abstract, introduction, and discussion that their major results are for human epithelial cells. However, they only prove these results using a cancer cell line (T84 human colon carcinoma). To make these claims, the authors must demonstrate their major results are applicable to non-cancer epithelial cells.

This is a very good point and we have performed key experiments in human ileum-derived organoids showing that the population context influence response to IFNs in primary IECs:

- Population context and cell-to-cell heterogeneity: Ileum-derived organoids were seeded at medium density on 8-well ibidi chambers (glass bottom). After differentiation, cells were treated from the apical side (basolateral side is attached to glass) with type I and type III IFNs. 24 h post-treatment, cells were fixed and immunostained for ISG15. We then quantifying the ISG expression at the edge or center of cell clusters, allowing to confirm the impact of the population context on cell-to-cell heterogeneity in a physiological primary epithelial cell model system. (Supp. Fig. 1D-E)
- IFN treatment of organoids from the apical or basolateral side: Ileum-derived organoids were seeded on transwells (as in Fig. 4A) allowing for formation of a monolayer of polarized cells through air-liquid interphase (as described in Wang et al., Cell 2019. PMID: 31708126). The polarized monolayer of cells was then treated from the apical or basolateral side with type I and type III IFNs, and the ISG induction was determined by q-RT-PCR. This showed in a more physiological model that intestinal epithelial cells are more responsive from the basolateral side, thereby confirming an asymmetric polarized response to IFNs. (Fig. 4D)
- IFN treatment of classical (basolateral-out) and inside-out (apical-out) 3D organoids: Organoids were grown as 3D structures. Classically, the organoids grow with the apical

side facing inside the structure and the basolateral side facing the matrigel (IFN treatment side), namely basolateral-out (BL-out) organoids. Additionally, we generate inside-out organoids, in which the basolateral side faces inwards while the apical side will face the matrigel and be accessible for IFN treatment, namely apical-out (A-out) organoids. Treatment of BL-out or A-out 3D organoids allowed for IFN interaction with solely the basolateral or apical side, respectively. Measurement of ISG expression by q-RT-PCR supported that the response to IFNs is polarized towards the basolateral side of IECs in 3D organoids (Fig. 4E-G)

2. Figures 2B-C clearly show a spatial heterogeneity in the radial direction. The authors should address this in the text and should also have a plot (either in main text or SI) of the entire distribution of mean fluorescence for edge versus center cells at the single-cell level. If possible, it would be interesting to find a value of fluorescence that separates the edge versus center cells and report what percentage of each population are classified correctly.

This is a good observation. We have addressed the radial direction of spatial heterogeneity in the text and plotted the distribution of mean fluorescence along the radial axis (Fig. 2C).

3. One question that arises is: do the center cells have a delayed response to stimulation? To address this question in relation to Figures 2B-C, it would be necessary to demonstrate that 24 hours is the maximal antiviral response. The authors should show a later timepoint where the signal is decreased. Also, Figure 3B demonstrates that the maximal response to IFN1-3 occurs after 24 hours, further motivating the necessity of including a later timepoint.

This is a critical point. In the revised version, we addressed it by measuring IFN responsiveness at later timepoints for several experiments:

- We quantified the spatial heterogeneity in prom-Mx1-mCherry expression as in Fig. 1A-C for the timepoints 6, 12, 24, 36, 48, 72 and 96 h post-IFN treatment. Results can be found in Fig. 1F, and we demonstrate that the edge effect is not time dependent but is conserved for all timepoints.
- We followed the expression of prom-Mx1-mCherry in cell population seeded on patterns for 48, 72 and 96 h post IFN treatment. Results are depicted in Fig. 2D.
- We measured the endogenous ISG expression with q-RT-PCR at high vs. low density at 36, 48, 72 and 96 h post IFN treatment. Results are depicted in Fig. 3B.

4. Could the authors comment on why there are significant differences between IFN stimulation of low-density populations upon ZO1-KO (e.g., the difference in ISG fold-change between WT versus ZO1-KO for low-density monolayers)? This potentially indicates that ZO1-KO itself influences the signal transduction of IFNs. The authors should include additional experiments measuring PSTAT activation for WT versus ZO1-KO cells to determine if ZO1-KO influences IFN activation.

Preliminary data in our laboratory revealed that cell density (as sensed by junctional complexes in which ZO1 is present) influences IFN signaling. Precisely, the basal immune response is affected by presence or absence of junctional complexes/ZO1. Since in our RT-q-PCR analysis we plot the fold change relative to mock, differences in basal IFN signaling (basal ISG expression) will have an effect on the fold change quantification as seen in Fig. 6F. We are currently investigating the molecular mechanism linking potentially ZO-1 and tight junction to IFN signaling. Like the reviewer, we believe that it is very interesting but we also think that this does not belong to the scope of this manuscript.

Nonetheless, to address the reviewer's concern, we treated confluent and sparse T84 WT and ZO1-KO cells with 2000 IU/mL IFNβ1 or 300ng/mL IFNλ1-3. After 1h, protein lysates were harvested and PSTAT1 levels were analyzed by WesternBlot (α-Tubulin served as a loading

control). Results can be seen below. Interestingly, while T84 WT cells show basal pSTAT1 levels at high density (mock treatment), these are not present in T84 ZO1-KO cells seeded at high density. Importantly, ZO1-KO cells at high density respond the same or even more to IFNs than ZO1-KO cells at low density, which is in agreement with the ISG expression data from Fig. 6F.,

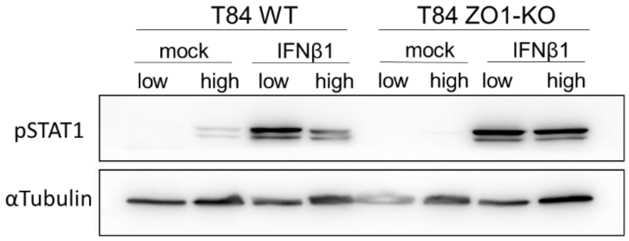

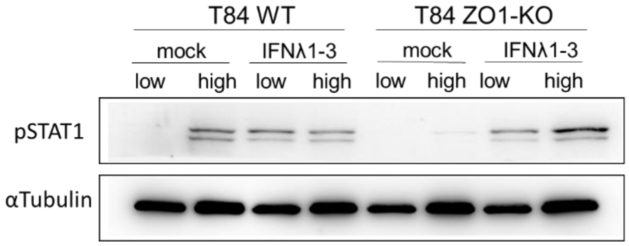

Additionally, several minor points should be addressed:

1. The authors claim that the edge degree is negatively correlated with response to IFN in Figure 1, but they only prove this by performing pairwise p-tests. A better metric to support this claim would be to calculate a correlation coefficient that describes the trend across edge degrees or performing a linear regression across edge degrees and reporting the regression coefficients.

This is a very good suggestion, and we now plotted a linear regression between positive cells and edge degree, and calculated the correlation coefficient. This is depicted in Fig. 1E and explained in the main text.

2. Figure 3 has a confusing representation. Figure 3B shows RT-qPCR measurements at 0, 6, 12, 18, and 24 hours but Figure 3A seems to show that only the 24-hour timepoint was collected.

Thanks for pointing it out, we updated the schematic.

In summary, I believe that this is an important piece of work for the systems biology community and should be published after the modifications outlined above are addressed.

8th Dec 2023

Manuscript Number: MSB-2023-11778RR
Title: Population context drives cell-to-cell variability in interferon response in epithelial cells

Dear Steeve,

Thank you for sending us your revised manuscript. We have now heard back from the three reviewers who were asked to evaluate your revised study. As you will see below, the reviewers think that the study has improved after the performed revisions. They raise however a series of remaining concerns, which we would ask you to address by text changes in a final round of revision. The remaining somewhat more major comments of reviewer #1 can also be addressed by text changes and without performing additional analyses. We would also ask you to address some remaining editorial issues listed below.

- Our data editors have noticed some unclear or missing information in the figure legends.
-- Please indicate the statistical test used for data analysis in the legends of figures 5b-c.
-- Information related to n is missing in the legend of figures 5b-c; figure EV3b
-- The error bars are not defined in the legend of figure EV3b."
-- The scale bar is missing for figure 6c.
-- Please indicate what red and yellow arrowheads represent in the legend of Figure EV1d

- The funding information listed in the submission system needs to match that listed in the text. Currently there is duplicated info and a couple of mistakes in the submission system: Deutsche Forschungsgemeinschaft (DFG) 240245660 and 278001972 (240245660 listed 3 times); UF | UF Health | College of Medicine, University of Florida (UF College of Medicine) start-up package (there is a typo in "start-up pachage"); in the manuscript file: grant number 240245660 is listed twice with different project numbers (SFB 1129 and SFB 1129 TP11). Please make sure that all information is accurate.

- Please provide 5 keywords.

- The References should be formatted according to the Molecular Systems Biology reference style (i.e., ordered alphabetically and listing the first 10 authors followed by et al).

- Please include the information about the availability of the mass spectrometry data in the Data Availability section, including the accession number and the link to the dataset. (Most authors use PRIDE or MASSIVE).
This section needs to be formatted according to the example below:
The datasets and computer code produced in this study are available in the following databases:
- Chip-Seq data: Gene Expression Omnibus GSE46748 (https://www.ncbi.nlm.nih.gov/geo/query/acc.cgi?acc=GSE46748)
- Modeling computer scripts: GitHub (https://github.com/SysBioChalmers/GECKO/releases/tag/v1.0)
- [data type]: [full name of the resource] [accession number/identifier] ([doi or URL or identifiers.org/DATABASE:ACCESSION])

- The section Conflict of Interest should be renamed to "Disclosure and Competing Interests Statement".

- Please note that our editorial policy does not allow "Data not Shown" (currently there is such a statement on page 8).

- The callouts for EV figures in the text should be corrected to Figure EV1, Figure EV2 ... Figure EV6. A callout for Dataset EV1is missing and needs to be included.

- Please include in each of the EV Datasets a description of the dataset as a separate sheet in the Excel file. The Datasets should be labelled and called out as Dataset EV1, Dataset EV2 etc.

- Please provide a "standfirst text" summarizing the study in one or two sentences (approximately 250 characters), three to four "bullet points" highlighting the main findings and a "synopsis image" (550px width and max 400px height, jpeg format) to highlight the paper on our homepage.

Please resubmit your revised manuscript online, with a cover letter listing amendments and responses to each point raised by the referees. Please resubmit the paper **within one month** and ideally as soon as possible. If we do not receive the revised manuscript within this time period, the file might be closed and any subsequent resubmission would be treated as a new manuscript. Please use the Manuscript Number (above) in all correspondence.

Click on the link below to submit your revised paper.

https://msb.msubmit.net/cgi-bin/main.plex

Thank you for submitting this paper to Molecular Systems Biology.

Kind regards,

Maria

Maria Polychronidou, PhD
Senior Editor
Molecular Systems Biology

If you do choose to resubmit, please click on the link below to submit the revision online before 7th Jan 2024.

https://msb.msubmit.net/cgi-bin/main.plex

IMPORTANT:
Please note that corresponding authors are required to supply an ORCID ID for their name upon submission of a revised manuscript (EMBO Press signed a joint statement to encourage ORCID adoption).
(https://www.embopress.org/page/journal/17444292/authorguide#editorialprocess)
Currently, our records indicate that the ORCID for your account is 0000-0001-8614-4993.

Please click the link below to modify this ORCID:
Link Not Available

As a matter of course, please make sure that you have correctly followed the instructions for authors as given on the submission website.

*** PLEASE NOTE *** As part of the EMBO Press transparent editorial process initiative (see our Editorial at https://dx.doi.org/10.1038/msb.2010.72 , Molecular Systems Biology will publish online a Review Process File to accompany accepted manuscripts. When preparing your letter of response, please be aware that in the event of acceptance, your cover letter/point-by-point document will be included as part of this File, which will be available to the scientific community. More information about this initiative is available in our Instructions to Authors. If you have any questions about this initiative, please contact the editorial office (msb@embo.org).

Reviewer #1:

In this re-submission, Metz-Zumaran et al have provided major changes to the manuscript. The additional experiments provided in the main and supplemental figures provided more convincing evidence demonstrating the importance of cellular density for regulating magnitude of induction of interferon responses in vitro. Their data indicates this phenotype is abrogated by basolateral stimulation with data suggesting a role of basolateral receptor localization in its regulation. While the data is convincing in supporting the claims regarding the cell density and receptor localization, they are less so for the claims related to the role of population context on single cell interferon responses.

Major points:
(i) The questions the authors have outlined in the introduction about the source of cell-cell heterogeneity is an important biological question, but most of the analysis provided does not allow for conclusions about heterogeneity due to single cell responses. While the imaging figures presented in Figs 1, 2, 7 provide some data that showcase the cell-to-cell variability in the single cell response, most of the quantitative data analysis are averages of subsets of the population based on spatial location or density (e.g. MFI of radial segmentations, fold change from bulk data). For more robust analysis of cell-to-cell heterogeneity in the various conditions, single cell tracking of the fluorescence intensity could be used and the distributions of the cell responses plotted.
(ii) Conclusions about heterogeneous responses have been made based on the characterization of cells as responders or non-responders. This is a binary categorization that lumps cells into two broad categories without considering the variability within the categories. Does the distribution of the magnitude of response within responders remain the same between edge and center cells?
(iii) I appreciate the fact that the authors have attempted to avoid including text in the manuscript that would claim that cell density and population context are the sole factor for heterogeneous IFN responses. The high percentage of non-responding edge or single cells (~40% for IFNβ1 and ~80% for IFNλ1-3 stimulations) even with a saturating dose of stimuli should still be addressed in the text since the focus of the manuscript stated in the introduction is to "understand population context" and "the

origins of cell-to-cell variability".
(iv) In the abstract section, the authors make the conclusion that the "cells embedded within cellular colonies are not protected by IFNs", but this conclusion is too general and fails to indicate that this population context phenotype did not occur when cells were stimulated on the basolateral side, an experimental set-up that is more physiologic. Including text in the abstract, similar to what was done in the conclusion, that discuss the importance of these findings for in vitro experimental set-ups should be addressed.
(v) The authors state in the discussion that their tools allow them to "better deconvolve stochastic or deterministic origin of cell-to-cell variability". This is overstated as none of the analysis sought to determine or compare the stochastic and deterministic sources of noise in the system.

Minor points:
(i) A few minor typos on page 5: "where even very high concentrations type III IFNs" and "for cell populations allows to tune geometry and size".
(ii) Supplemental Fig 3B: the y-axis label of "cell number" although it was a percentage was a bit confusing.

Reviewer #2:

The authors have been extremely responsive to the initial reviews. This includes adding the additional epithelial and fibroblast cell lines and the human ileum-derived organoids, as well as the time-resolved measurements. This strengthens their conclusion that population context explains a significant amount of the heterogeneity observed in the interferon response of epithelial cells specifically. They also significantly revised the introduction and discussion in response to comments raised.

Minor point: In the end, the authors conclude that population context is one factor "influencing" cell-to-cell heterogeneity in the epithelial IFN response. To make a stronger statement, the authors could consider quantifying the extent to which cell-to-cell heterogeneity is reduced once population context is accounted for. A larger point of this study is that non-genetic heterogeneity can sometimes be largely explained by unmeasured differences in biology, and thus some biological processes are more deterministic than they appear to be.

Reviewer #3:

Metz-Zumaran et al. significantly revised their manuscript to address key concerns about extensibility of findings, qualifying their claims about the importance of the population context, providing additional temporal data on IFN responses, addressing the effects of ZO1-KO on IFN stimulation, as well as performing more quantitative analyses. Importantly, by confirming major findings in several different cell lines (epithelial cells of different origin, nonepithelial cell line, and 3D organoids) they better solidify their argument that the population context is an important determinant of response to IFNg and could also apply to in vivo contexts.

Before final publication, the authors should address several minor points related to analysis and presentation of existing data.

1. In Figure 1E, the authors use linear regression to quantify the correlation between edge degree and % positive cells. However, % positive cells is a continuous variable while edge degree is a discrete variable. Alternatively, the authors should use Poisson regression or Negative Binomial regression, depending on the edge degree distribution (i.e., if mean = variance use Poisson regression).
2. In Figure 1 the authors quantify IFN response by % positive cells (i.e., % responders), while in Figure 2 this is quantified by mean fluorescence. As mean fluorescence averages the fluorescent signal of responders and non-responders, the authors should show if the change in mean fluorescence is due to a change in % responders or a change in the mean fluorescence of the responder population. For example, adding an extra panel to Supplementary Figure 1 with the mean fluorescence of responders corresponding to the same conditions as Figure 1E. And having another supplementary figure with % responders and mean fluorescence of responders corresponding to the same conditions as Figure 2C&D.
3. To improve the clarity of Figure 4 for the reader, include a title for each panel specifying the corresponding cell line used to generate the data.
4. The layout of Figure 5 as presented is confusing.
a. Could the authors move Panel 5B to the supplementary material and just add an equation for the AP/BL ratio that includes the biotinylation/no biotinylation concept.
b. Figure 5C shows that the p-value for IFNAR2 is not significant. Could the authors acknowledge this somewhere in the text and provide a potential explanation.
c. The vertical lines corresponding to 80% enrichment should be dropped because they don't seem to convey relevant information for interpretation of the plot with respect to IFNAR2 and IL10RB.
d. It would also be helpful to have a panel with a bar graph of fold change for AP/BL ratio for IFNAR2 and IL10RB compared to apical and basal control markers. It is difficult to parse this information from the volcano plot.

Dear MSB editorial team and reviewers,

We thank both the reviewers and editors for the time they have invested in reviewing this manuscript (MSB-2023-11778RR) and for their valuable inputs. We feel that this review process allowed us to significantly improve the clarity of the overall manuscript.

Please find below our point-by-point answer to both the editorial issues and reviewer comments.

Our answers will be color-coded in blue.

All the best,

Steeve Boulant and Camila Metz Zumaran in the name of all co-authors

Editorial issues

a. Our data editors have noticed some unclear or missing information in the figure legends.

- Please indicate the statistical test used for data analysis in the legends of figures 5b-c.
- Information related to n is missing in the legend of figures 5b-c; figure EV3b
- The error bars are not defined in the legend of figure EV3b.
- The scale bar is missing for figure 6c.
- Please indicate what red and yellow arrowheads represent in the legend of Figure EV1d

Done.

b. The funding information listed in the submission system needs to match that listed in the text. Currently there is duplicated info and a couple of mistakes in the submission system: Deutsche Forschungsgemeinschaft (DFG) 240245660 and 278001972 (240245660 listed 3 times); UF | UF Health | College of Medicine, University of Florida (UF College of Medicine) start-up package (there is a typo in "start-up pachage"); in the manuscript file: grant number 240245660 is listed twice with different project numbers (SFB 1129 and SFB 1129 TP11). Please make sure that all information is accurate.

Done.

c. Please provide 5 keywords.

Interferons, Heterogeneity, Population context, Epithelium, Polarity

d. The References should be formatted according to the Molecular Systems Biology reference style (i.e., ordered alphabetically and listing the first 10 authors followed by et al).

Done.

e. Please include the information about the availability of the mass spectrometry data in the Data Availability section, including the accession number and the link to the dataset. (Most authors use PRIDE or MASSIVE).
This section needs to be formatted according to the example below:
The datasets and computer code produced in this study are available in the following databases:
- Chip-Seq data: Gene Expression Omnibus GSE46748

(https://www.ncbi.nlm.nih.gov/geo/query/acc.cgi?acc=GSE46748)
- Modeling computer scripts: GitHub
(https://github.com/SysBioChalmers/GECKO/releases/tag/v1.0)
- [data type]: [full name of the resource] [accession number/identifier] ([doi or URL or identifiers.org/DATABASE:ACCESSION])

Done.

f. The section Conflict of Interest should be renamed to "Disclosure and Competing Interests Statement".

Done.

g. Please note that our editorial policy does not allow "Data not Shown" (currently there is such a statement on page 8).

Done.

h. The callouts for EV figures in the text should be corrected to Figure EV1, Figure EV2 ... Figure EV6. A callout for Dataset EV1is missing and needs to be included.

Done.

i. Please include in each of the EV Datasets a description of the dataset as a separate sheet in the Excel file. The Datasets should be labelled and called out as Dataset EV1, Dataset EV2 etc.

Done.

j. Please provide a "standfirst text" summarizing the study in one or two sentences (approximately 250 characters), three to four "bullet points" highlighting the main findings and a "synopsis image" (550px width and max 400px height, jpeg format) to highlight the paper on our homepage.

Standfirst text: The heterogeneous response to interferons in epithelial cells is determined by the population context through cell polarization and basolateral localization of the interferon receptor.

Bullet points:

- Isogenic epithelial cells respond in a heterogeneous manner to type I and type III interferons.
- The population context (position of a cell within a population) drives the heterogeneous response to interferons, with cells located at the edge of a population being more responsive than cells located in the center of a population.
- Cells located in the center of a population are refractive to interferons treatment from their apical side due to the polarized basolateral distribution of the interferon receptors.
- By controlling response to interferon treatment, the population context impacts susceptibility to viral infection.

Synopsis image:

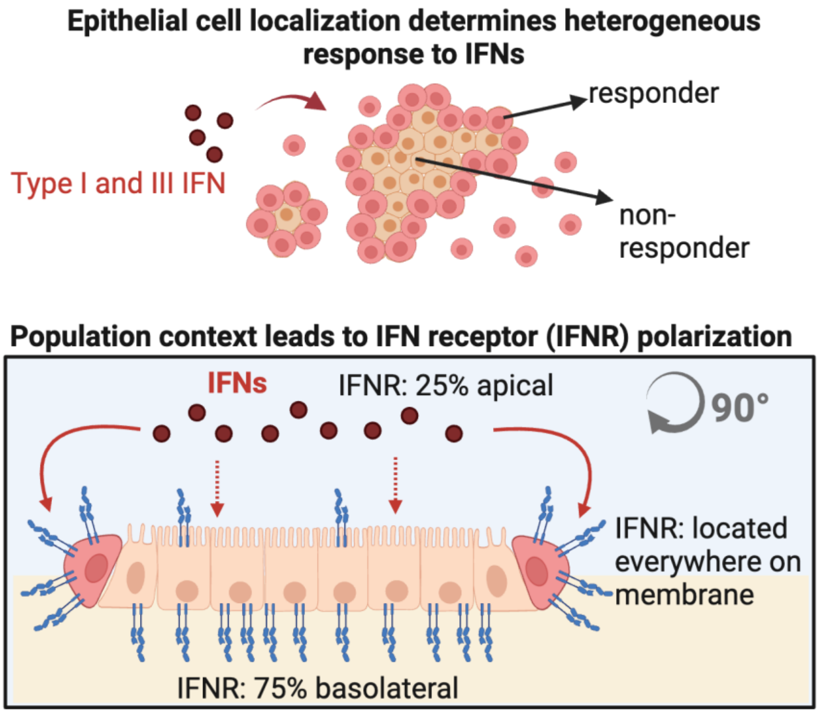

Answer to the reviewers

Reviewer #1:

In this re-submission, Metz-Zumaran et al have provided major changes to the manuscript. The additional experiments provided in the main and supplemental figures provided more convincing evidence demonstrating the importance of cellular density for regulating magnitude of induction of interferon responses in vitro. Their data indicates this phenotype is abrogated by basolateral stimulation with data suggesting a role of basolateral receptor localization in its regulation. While the data is convincing in supporting the claims regarding the cell density and receptor localization, they are less so for the claims related to the role of population context on single cell interferon responses.

Major points:
(i) The questions the authors have outlined in the introduction about the source of cell-cell heterogeneity is an important biological question, but most of the analysis provided does not allow for conclusions about heterogeneity due to single cell responses. While the imaging figures presented in Figs 1, 2, 7 provide some data that showcase the cell-to-cell variability in the single cell response, most of the quantitative data analysis are averages of subsets of the population based on spatial location or density (e.g. MFI of radial segmentations, fold change from bulk data). For more robust analysis of cell-to-cell heterogeneity in the various conditions, single cell tracking of the fluorescence intensity could be used and the distributions of the cell responses plotted.

This is a valid point raised by the reviewer. Indeed, in the introduction we did focus on cell-to-cell heterogeneity during IFN signaling in an isogenic cell population. However, the aim of this paper was not to study cell-to-cell variability itself but to understand whether the population context is contributing to a heterogeneous response within the population. We believe that our analyses on subsets of the population (based on spatial location or density) rather than analysis of single cells are adequate to address the importance of the population context. We understand that the way we insisted on the cell-to-cell variability in our introduction may lead to some confusions for the readers. As such, to increase the readability of our manuscript, we have modified the title, abstract and the main body (mostly the introduction), thereby shifting the focus of the study to the origins of heterogeneity rather than the single cell variability itself.

(ii) Conclusions about heterogeneous responses have been made based on the characterization of cells as responders or non-responders. This is a binary categorization that lumps cells into two broad categories without considering the variability within the categories. Does the distribution of the magnitude of response within responders remain the same between edge and center cells?

This is an excellent point raised by the reviewer. Indeed, in Figure 1 we characterized the heterogeneous response to IFNs by classifying into responder and non-responder cells, without directly addressing the variability within the responder population. To address this point, we now plotted the normalized fluorescence of every responder cell located at the edge or at the center of a population (graphs below). Using an unpaired t test with Welch's correction, we can show that responder edge cells showed an overall higher normalized fluorescence as compared to cells located at the center of a population. Altogether we can conclude that the distribution of the magnitude of response within responder cells is similar between edge and center, with edge cells having an overall higher normalized fluorescence.

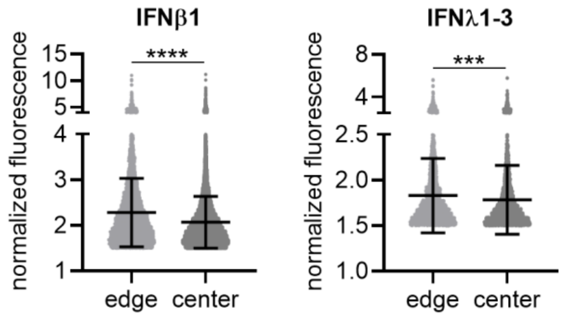

(iii) I appreciate the fact that the authors have attempted to avoid including text in the manuscript that would claim that cell density and population context are the sole factor for heterogeneous IFN responses. The high percentage of non-responding edge or single cells (~40% for IFNβ1 and ~80% for IFNλ1-3 stimulations) even with a saturating dose of stimuli should still be addressed in the text since the focus of the manuscript stated in the introduction is to "understand population context" and "the origins of cell-to-cell variability".

This is an important point and is now directly addressed in the discussion. Please find below the citation:

"Although the responsiveness of epithelial cells in this study can be partially ascribed to the basolateral localization of IFN receptors, this explanation does not fully account for the observed variations in the amplitude of individual cell responses (reflected by the intensity of fluorescent ISG reporter expression) when subjected to apical and basolateral treatments (Fig. 1-2, Fig. 4, and Figure EV1). Moreover, although within our model we anticipate that every edge and single cell would respond to apical IFN treatment, a considerable proportion of these cells still do not exhibit a response even at saturating dose of stimuli (Fig. 1). For example, around 20% of single cells did not respond to apical IFNβ1 treatment and 60% did not respond to apical IFNλ1-3 treatment (Fig. 1F). These observations strongly suggest that additional mechanisms drive a heterogeneous response in epithelial cells, which could originate from stochastic events as described before (Maier et al, 2022; Rand et al, 2012). For example, abundance of signaling molecules as the IFN receptor or STATs could play a role in making cells in a population refractory to IFN signaling. We suggest that, in addition to the population context, other factors, whether of deterministic or stochastic origin, influence cell-to-cell variability in isogenic epithelial cell populations."

(iv) In the abstract section, the authors make the conclusion that the "cells embedded within cellular colonies are not protected by IFNs", but this conclusion is too general and fails to indicate that this population context phenotype did not occur when cells were stimulated on the basolateral side, an experimental set-up that is more physiologic. Including text in the abstract, similar to what was done in the conclusion, that discuss the importance of these findings for in vitro experimental set-ups should be addressed.

Thank you for the comment. We changed the abstract accordingly.

(v) The authors state in the discussion that their tools allow them to "better deconvolve stochastic or deterministic origin of cell-to-cell variability". This is overstated as none of the analysis sought to determine or compare the stochastic and deterministic sources of noise in the system.

We agree with the reviewers comment and removed the statement from the discussion.

Minor points:
(i) A few minor typos on page 5: "where even very high concentrations type III IFNs" and "for cell populations allows to tune geometry and size".

Thank you, we fixed the typos.

(ii) Supplemental Fig 3B: the y-axis label of "cell number" although it was a percentage was a bit confusing.
We changed it to cell percentage.

Reviewer #2:

The authors have been extremely responsive to the initial reviews. This includes adding the additional epithelial and fibroblast cell lines and the human ileum-derived organoids, as well as the time-resolved measurements. This strengthens their conclusion that population context explains a significant amount of the heterogeneity observed in the interferon response of epithelial cells specifically. They also significantly revised the introduction and discussion in response to comments raised.

Minor point: In the end, the authors conclude that population context is one factor "influencing" cell-to-cell heterogeneity in the epithelial IFN response. To make a stronger statement, the authors could consider quantifying the extent to which cell-to-cell heterogeneity is reduced once population context is accounted for. A larger point of this study is that non-genetic heterogeneity can sometimes be largely explained by unmeasured differences in biology, and thus some biological processes are more deterministic than they appear to be.

Thank you very much for this comment. We believe that accounting for the populations context will explain a considerable amount of the cell-to-cell heterogeneity after IFN treatment. With our current work, we can explained 80% of heterogeneity for IFNβ1 treatment, since only 20% of single cells do not respond after the treatment (and we cannot explain why these 20% do not respond). However, it gets complicated when we look at edge cells, where only 65% respond after IFNβ1 treatment, although we expect full responsiveness. Moreover, this doesn't account for the magnitude in response, which also is an important form of heterogeneity. By using mathematical models, we could indeed quantify the extent to which cell-to-cell heterogeneity is reduced when accounting for the population context, however we feel that this approach would constitute a complete new study. We have addressed this point in the discussion, please see the citation below:

"With our study we identified a parameter (the population context) that drives the response to IFNs in epithelial cells. Once factoring the population context into an experiment, it will significantly diminish the level of 'unexplained' heterogeneity. This outlines that non-genetic heterogeneity can sometimes be largely explained by yet unmeasured differences in biology, and thus some biological processes are more deterministic than initially thought."

Reviewer #3:

Metz-Zumaran et al. significantly revised their manuscript to address key concerns about extensibility of findings, qualifying their claims about the importance of the population context, providing additional temporal data on IFN responses, addressing the effects of ZO1-

KO on IFN stimulation, as well as performing more quantitative analyses. Importantly, by confirming major findings in several different cell lines (epithelial cells of different origin, nonepithelial cell line, and 3D organoids) they better solidify their argument that the population context is an important determinant of response to IFNg and could also apply to in vivo contexts.

Before final publication, the authors should address several minor points related to analysis and presentation of existing data.

1. In Figure 1E, the authors use linear regression to quantify the correlation between edge degree and % positive cells. However, % positive cells is a continuous variable while edge degree is a discrete variable. Alternatively, the authors should use Poisson regression or Negative Binomial regression, depending on the edge degree distribution (i.e., if mean = variance use Poisson regression).

We thank the reviewer for this important comment. The reviewer is correct that the used Pearson correlation is not appropriate given the combination of ordinal (edge degree) and continuous (% of positive cells) data. However, the suggested Poisson or Negative-Binomial regression are also not fully applicable as we have to work with the % of positive cells, and not absolute count data, in order to appropriately account for the heterogeneity between different fields of view that potentially comprise slightly different cellular densities. We therefore re-did the analysis using the non-parametric Spearman-Correlation, which is appropriate for the given types of data, to evaluate the relationship between the % of positive cells and the edge degree distribution. We observed a significant ($p<0.0001$) negative correlation between the edge degree and the percentage of cells responding to both IFNs ($\rho=0.8266$ for IFNβ1 and $\rho=0.8571$ for IFNλ1-3 treatment).

2. In Figure 1 the authors quantify IFN response by % positive cells (i.e., % responders), while in Figure 2 this is quantified by mean fluorescence. As mean fluorescence averages the fluorescent signal of responders and non-responders, the authors should show if the change in mean fluorescence is due to a change in % responders or a change in the mean fluorescence of the responder population. For example, adding an extra panel to Supplementary Figure 1 with the mean fluorescence of responders corresponding to the same conditions as Figure 1E. And having another supplementary figure with % responders and mean fluorescence of responders corresponding to the same conditions as Figure 2C&D.

This is an excellent comment. To address this, we plotted the normalized fluorescence of the responder cells for each edge degree upon type I and III IFN treatment (corresponding to Figure 1E). This is visualized in Figure EV1C and explained in the results section. Interestingly, we observe a decrease in the normalized fluorescence with increased edge degree for type I IFN treatment, but not for type III IFN treatment. This suggest that the change in the normalized fluorescence in Figure 1D (and most probably also for the micropatterned cell populations of Figure 2C) is due to a change of both % and magnitude of response for type I IFN treatment, but only of % of responder cells for type III IFN treatment. Unfortunately, due to technical limitations we cannot segment each individual cells seeded on micropatterns to obtain a single cell resolution (the cells are so dense, that it is not possible to reliably segment them to calculate their position). Hence, we cannot determine the percentage of responder cells nor the mean fluorescence of those responder population of the data from Figure 2C&D. However, we believe that by providing the data depicted in Figure EV1C, we at least partially addressed the reviewer comment.

3. To improve the clarity of Figure 4 for the reader, include a title for each panel specifying the corresponding cell line used to generate the data.

Thank you for raising that point, we now added a title for each panel specifying the corresponding cell line.

4. The layout of Figure 5 as presented is confusing.

a. Could the authors move Panel 5B to the supplementary material and just add an equation for the AP/BL ratio that includes the biotinylation/no biotinylation concept.

Thank you for the comment. Panel 5B is now in the supplementary material as EV7.

Here is an explanation with equation for the AP/BL ratio that includes the biotinylation/no biotinylation concept: The biotinylated proteins are filtered in advance by using a non-biotinylated control sample (containing i.e. proteins that non-specifically bind the streptavidin beads used for purification, from a whole T84 cell lysate in the same transwell setting). This is done using the volcano plot function of the Perseus software, where Biotinylated apical and Biotinylated basolateral samples are matched against the non-biotinylated control using a t-test with FDR of 0.05 and an s0 = 0.1. Then two different databases are obtained, one with enriched apical biotinylated proteins and one with enriched basolateral biotinylated proteins. These databases are fused together by matching columns by name, duplicates are eliminated and then LFQ values for each apical and basolateral replicate are considered in the following equation $\frac{Apical\ LFQ\ value}{Apical\ LFQ\ value + Basolateral\ LFQ\ value} = Apical\ index$. A volcano plot is also generated by matching apical biotinylation samples against basolateral biotinylation samples using the same settings as above.

This explanation was also added in the manuscript.

b. Figure 5C shows that the p-value for IFNAR2 is not significant. Could the authors acknowledge this somewhere in the text and provide a potential explanation.

This is an important comment. Indeed, we detected some variability in the expression in the different replicates. While on the apical side the protein was recovered only in 2 out of 4 total samples, it has always been found in the basolateral samples (4 out of 4), however with different levels of expression (see table below). This variability of expression on the basolateral side in the different replicates leads to the non-significant enrichment of the protein according to the t-test with FDR 0.05 used in the volcano plot. We now acknowledged this in the manuscript, also providing this potential explanation.

Type	LFQ intensity Apical_...	LFQ intensity Apical_...	LFQ intensity Apical_...	LFQ intensity Apical_...	LFQ intensity Apical_...	LFQ intensity Apical_...	LFQ intensity Backgr...	LFQ intensity Backgr...	LFQ intensity Backgr...	LFQ intensity Backgr...	LFQ intensity Backgr...	LFQ intensity Backgr...	LFQ intensity Basola...	LFQ intensity Basola...	LFQ intensity Basola...	LFQ intensity Basola...	LFQ intensity Basola...	LFQ intensity Basola...
	Main	Main	Main	Main	Main	Main	Main	Main	Main	Main	Main	Main	Main	Main	Main	Main	Main	Main
1197	26.1228	26.4714	26.4298	25.3752	26.4141	24.4818	NaN	NaN	NaN	NaN	NaN	NaN	26.192	27.5428	27.0357	28.4845	28.6542	28.8217
1198	NaN	23.2385	23.0438	NaN	NaN	NaN	NaN	NaN	NaN	NaN	NaN	NaN	23.4955	24.7218	23.5443	23.7494	23.8432	23.5677
1199	25.4932	25.2025	25.2978	24.8355	24.8098	25.0978	25.5968	25.5197	25.233	25.1098	25.2979	NaN	25.1365	24.3646	24.4315	25.0367	25.1886	24.9963
1200	NaN	NaN	NaN	NaN	NaN	NaN	NaN	NaN	NaN	NaN	NaN	NaN	NaN	NaN	NaN	NaN	NaN	NaN
1201	23.0921	NaN	NaN	23.9541	NaN	23.5676	NaN	NaN	NaN	NaN	NaN	NaN	23.3508	NaN	NaN	NaN	NaN	NaN
1202	29.164	29.1002	28.9466	29.0843	28.98	29.3406	28.9462	28.4906	28.4049	28.8653	29.1431	28.7066	29.3653	29.2275	29.2079	29.0332	29.4709	29.3696
1203	24.3086	24.5411	24.4352	24.6927	24.8028	25.0934	NaN	NaN	NaN	NaN	NaN	NaN	25.5232	24.5056	24.1673	24.4923	24.6772	NaN
1204	26.7867	26.0995	26.4083	26.0811	26.0813	26.3935	NaN	NaN	25.7616	NaN	25.6275	NaN	26.1437	26.1939	26.1152	26.538	26.3725	26.2938
1205	25.8564	26.1071	26.2713	25.8438	25.6268	25.9171	NaN	NaN	NaN	24.3096	NaN	NaN	25.786	25.364	25.9771	25.0876	25.3976	25.259

	C: Potential contam...	N: Peptides	N: Razor + unique peptides	N: Unique peptides	N: Seque... covera...	N: Unique + razor	N: Unique sequen...	N: Mol. weight [kDa]	N: Q-value	N: Score	N: Intensity	N: iBAQ peptides	N: MS/MS count	T: Protein IDs	T: Majority protein	T: Protein names	T: Gene names	T: id
Type	Catego...	Numeric	Numeric	Numeric	Numeric	Numeric	Numeric	Numeric	Numeric	Numeric	Numeric	Numeric	Numeric	Text	Text	Text	Text	Text
1197		7	7	7	20.9	20.9	20.9	28.295	0	61.869	353590...	10	33	P48509	P48509	CD151...	CD151	1341
1198		3	3	3	7.4	7.4	7.4	57.758	0	10.415	203040...	16	8	P48551	P48551	Interfe...	IFNAR2	1342
1199		7	7	7	26.9	26.9	26.9	39.611	0	22.29	869040...	20	38	P48556	P48556	26S pr...	PSMD8	1343
1200		14	1	1	37.2	4.9	4.9	44.853	0	5.3495	0	21	1	P48594	P48594	Serpin...	SERPI...	1344
1201		2	2	2	7.8	7.8	7.8	52.384	0.0006...	3.1445	169580...	27	12	P48637	P48637	Glutath...	GSS	1345
1202		30	30	30	73.4	73.4	73.4	59.67	0	323.31	146430...	33	452	P48643	P48643	T-com...	CCT5	1346
1203		4	4	4	10.4	10.4	10.4	55.527	0	7.0948	539930...	23	23	P48651	P48651	Phosp...	PTDSS1	1347
1204		12	12	12	43	43	43	38.914	0	23.106	181610...	19	52	P4872...	P48729	Casein...	CSNK1...	1348
1205		12	12	4	34	34	14	47.33	0	23.762	118890...	23	40	P48730	P48730	Casein...	CSNK1...	1349

c. The vertical lines corresponding to 80% enrichment should be dropped because they don't seem to convey relevant information for interpretation of the plot with respect to IFNAR2 and IL10RB.

We dropped the vertical lines of the plot (now Figure 5B).

d. It would also be helpful to have a panel with a bar graph of fold change for AP/BL ratio for IFNAR2 and IL10RB compared to apical and basal control markers. It is difficult to parse this information from the volcano plot.

Thank you for the comment. In Figure 5C we now provide a bar graph with the apical index for IFNAR2 and IL10RB compared to the apical marker ANPEP and the basolateral marker ATP1A1.

4th Jan 2024

Manuscript number: MSB-2023-11778RRR
Title: The population context is a driver of the heterogeneous response of epithelial cells to interferons

Dear Steeve,

Thank you again for sending us your revised manuscript. We are now satisfied with the modifications made and I am pleased to inform you that your paper has been accepted for publication.

Your manuscript will be processed for publication by EMBO Press. It will be copy edited and you will receive page proofs prior to publication. Please note that you will be contacted by Springer Nature Author Services to complete licensing and payment information.

You may qualify for financial assistance for your publication charges - either via a Springer Nature fully open access agreement or an EMBO initiative. Check your eligibility: https://www.embopress.org/page/journal/17444292/authorguide#chargesguide

Should you be planning a Press Release on your article, please get in contact with embo_production@springernature.com as early as possible in order to coordinate publication and release dates.

If you have any questions, please do not hesitate to contact the Editorial Office. Thank you for your contribution to Molecular Systems Biology.

Best wishes and Happy New Year,

Maria

Maria Polychronidou, PhD
Senior Editor
Molecular Systems Biology

>>> Please note that it is Molecular Systems Biology policy for the transcript of the editorial process (containing referee reports and your response letter) to be published as an online supplement to each paper. If you do NOT want this, you will need to inform the Editorial Office via email immediately. More information is available here: https://www.embopress.org/transparent-process#Review_Process

EMBO Press Author Checklist

Corresponding Author Name: Steeve Boulant	
Journal Submitted to: Molecular Systems Biology	
Manuscript Number: MSB-2023-11778R-Q	

USEFUL LINKS FOR COMPLETING THIS FORM
The EMBO Journal - Author Guidelines
EMBO Reports - Author Guidelines
Molecular Systems Biology - Author Guidelines
EMBO Molecular Medicine - Author Guidelines

Reporting Checklist for Life Science Articles (updated January
This checklist is adapted from Materials Design Analysis Reporting (MDAR) Checklist for Authors. MDAR establishes a minimum set of requirements in transparent reporting in the life sciences (see Statement of Task: 10.31222/osf.io/9sm4x). Please follow the journal's guidelines in preparing your
Please note that a copy of this checklist will be published alongside your article.

Abridged guidelines for figures
1. Data
The data shown in figures should satisfy the following conditions:
- the data were obtained and processed according to the field's best practice and are presented to reflect the results of the experiments in an accurate and unbiased manner.
- ideally, figure panels should include only measurements that are directly comparable to each other and obtained with the same assay.
- plots include clearly labeled error bars for independent experiments and sample sizes. Unless justified, error bars should not be shown for technical
- if n<5, the individual data points from each experiment should be plotted. Any statistical test employed should be justified.
- Source Data should be included to report the data underlying figures according to the guidelines set out in the authorship guidelines on Data

2. Captions
Each figure caption should contain the following information, for each panel where they are relevant:
- a specification of the experimental system investigated (eg cell line, species name).
- the assay(s) and method(s) used to carry out the reported observations and measurements.
- an explicit mention of the biological and chemical entity(ies) that are being measured.
- an explicit mention of the biological and chemical entity(ies) that are altered/varied/perturbed in a controlled manner.
- the exact sample size (n) for each experimental group/condition, given as a number, not a range;
- a description of the sample collection allowing the reader to understand whether the samples represent technical or biological replicates (including how many animals, litters, cultures, etc.).
- a statement of how many times the experiment shown was independently replicated in the laboratory.
- definitions of statistical methods and measures:
  - common tests, such as t-test (please specify whether paired vs. unpaired), simple χ2 tests, Wilcoxon and Mann-Whitney tests, can be unambiguously identified by name only, but more complex techniques should be described in the methods section;
  - are tests one-sided or two-sided?
  - are there adjustments for multiple comparisons?
  - exact statistical test results, e.g., P values = x but not P values < x;
  - definition of 'center values' as median or average;
  - definition of error bars as s.d. or s.e.m.

Please complete ALL of the questions below.
Select "Not Applicable" only when the requested information is not relevant for your study.

Materials

Newly Created Materials	Information included in the manuscript?	In which section is the information available? (Reagents and Tools Table, Materials and Methods, Figures, Data Availability Section)
New materials and reagents need to be available; do any restrictions apply?	Yes	T84 ZO1 KO cell line was newly created. It is described in the Material and Methods section and available upon request.

Antibodies	Information included in the manuscript?	In which section is the information available? (Reagents and Tools Table, Materials and Methods, Figures, Data Availability Section)
For **antibodies** provide the following information:<br>- Commercial antibodies: RRID (if possible) or supplier name, catalogue number and or/clone number<br>- Non-commercial: RRID or citation	Yes	Material and Methods section

DNA and RNA sequences	Information included in the manuscript?	In which section is the information available? (Reagents and Tools Table, Materials and Methods, Figures, Data Availability Section)
**Short novel DNA or RNA including primers, probes**: provide the sequences.	Yes	Table 2 in the Materials and Methods section

Cell materials	Information included in the manuscript?	In which section is the information available? (Reagents and Tools Table, Materials and Methods, Figures, Data Availability Section)
**Cell lines:** Provide species information, strain. Provide accession number in repository **OR** supplier name, catalog number, clone number, and/**OR** RRID.	Yes	Material and Methods section
**Primary cultures:** Provide species, strain, sex of origin, genetic modification status.	Yes	Material and Methods section
Report if the cell lines were recently **authenticated** (e.g., by STR profiling) and tested for mycoplasma contamination.	Yes	Material and Methods section

Experimental animals	Information included in the manuscript?	In which section is the information available? (Reagents and Tools Table, Materials and Methods, Figures, Data Availability Section)
**Laboratory animals or Model organisms:** Provide species, strain, sex, age, genetic modification status. Provide accession number in repository **OR** supplier name, catalog number, clone number, **OR** RRID.	Not Applicable	
**Animal observed in or captured from the field:** Provide species, sex, and age where possible**.**	Not Applicable	
Please detail **housing and husbandry conditions**.	Not Applicable	

Plants and microbes	Information included in the manuscript?	In which section is the information available? (Reagents and Tools Table, Materials and Methods, Figures, Data Availability Section)
**Plants:** provide species and strain, ecotype and cultivar where relevant, unique accession number if available, and source (including location for collected wild specimens).	Not Applicable	
**Microbes:** provide species and strain, unique accession number if available, and source.	Yes	Material and methods section

Human research participants	Information included in the manuscript?	In which section is the information available? (Reagents and Tools Table, Materials and Methods, Figures, Data Availability Section)
If collected and within the bounds of privacy constraints report on age, sex and gender or ethnicity for all study participants.	Not Applicable	

Core facilities	Information included in the manuscript?	In which section is the information available? (Reagents and Tools Table, Materials and Methods, Figures, Data Availability Section)
If your work benefited from core facilities, was their service mentioned in the acknowledgments section?	Yes	Acknowledgments section

Design

Study protocol	Information included in the manuscript?	In which section is the information available? (Reagents and Tools Table, Materials and Methods, Figures, Data Availability Section)
If study protocol has been **pre-registered, provide DOI in the manuscript**. For clinical trials, provide the trial registration number **OR** cite DOI.	Not Applicable	
Report the **clinical trial registration number** (at ClinicalTrials.gov or equivalent), where applicable.	Not Applicable	

Laboratory protocol	Information included in the manuscript?	In which section is the information available? (Reagents and Tools Table, Materials and Methods, Figures, Data Availability Section)
Provide DOI OR other citation details if **external detailed step-by-step protocols** are available.	Yes	Material and Methods section

Experimental study design and statistics	Information included in the manuscript?	In which section is the information available? (Reagents and Tools Table, Materials and Methods, Figures, Data Availability Section)
Include a statement about **sample size** estimate even if no statistical methods were used.	Yes	Figure Legends
Were any steps taken to minimize the effects of subjective bias when allocating animals/samples to treatment (e.g. **randomization procedure**)? If yes, have they been described?	Not Applicable	
Include a statement about **blinding** even if no blinding was done.	Not Applicable	
Describe **inclusion/exclusion criteria** if samples or animals were excluded from the analysis. Were the criteria pre-established?<br><br>If sample or data points were omitted from analysis, report if this was due to attrition or intentional exclusion and provide justification.	Not Applicable	
For every figure, are **statistical tests** justified as appropriate? Do the data meet the assumptions of the tests (e.g., normal distribution)? Describe any methods used to assess it. Is there an estimate of variation within each group of data? Is the variance similar between the groups that are being statistically compared?	Yes	Figure Legends

Sample definition and in-laboratory replication	Information included in the manuscript?	In which section is the information available? (Reagents and Tools Table, Materials and Methods, Figures, Data Availability Section)
In the figure legends: state number of times the experiment was **replicated** in laboratory.	Yes	Figure Legends
In the figure legends: define whether data describe **technical or biological replicates**.	Yes	Figure Legends

Ethics

Ethics	Information included in the manuscript?	In which section is the information available? (Reagents and Tools Table, Materials and Methods, Figures, Data Availability Section)
Studies involving **human participants**: State details of **authority granting ethics approval** (IRB or equivalent committee(s), provide reference number for approval.	Not Applicable	
Studies involving **human participants**: Include a statement confirming that **informed consent** was obtained from all subjects and that the experiments conformed to the principles set out in the WMA Declaration of Helsinki and the Department of Health and Human Services Belmont Report.	Not Applicable	
Studies involving **human participants:** For publication of **patient photos**, include a statement confirming that consent to publish was obtained.	Not Applicable	
Studies involving experimental **animals**: State details of **authority granting ethics approval** (IRB or equivalent committee(s), provide reference number for approval. Include a statement of compliance with ethical regulations.	Not Applicable	
Studies involving **specimen and field samples:** State if relevant **permits** obtained, provide details of authority approving study; if none were required, explain why.	Yes	Material and Methods section

Dual Use Research of Concern (DURC)	Information included in the manuscript?	In which section is the information available? (Reagents and Tools Table, Materials and Methods, Figures, Data Availability Section)
Could your study fall under dual use research restrictions? Please check biosecurity documents and list of **select agents and toxins** (CDC): https://www.selectagents.gov/sat/list.htm	Not Applicable	
If you used a select agent, is the security level of the lab appropriate and reported in the manuscript?	Not Applicable	
If a study is subject to dual use research of concern regulations, is the name of the **authority granting approval and reference number** for the regulatory approval provided in the manuscript?	Not Applicable	

Reporting

The MDAR framework recommends adoption of discipline-specific guidelines, established and endorsed through community initiatives. Journals have their own policy about requiring specific guidelines and recommendations to complement MDAR.

Adherence to community standards	Information included in the manuscript?	In which section is the information available? (Reagents and Tools Table, Materials and Methods, Figures, Data Availability Section)
State if relevant guidelines or checklists (e.g., **ICMJE, MIBBI, ARRIVE, PRISMA**) have been followed or provided.	Not Applicable	
For **tumor marker prognostic studies,** we recommend that you follow the **REMARK** reporting guidelines (see link list at top right). See author guidelines, under 'Reporting Guidelines'. Please confirm you have followed these guidelines.	Not Applicable	
For **phase II and III randomized controlled trials**, please refer to the **CONSORT** flow diagram (see link list at top right) and submit the CONSORT checklist (see link list at top right) with your submission. See author guidelines, under 'Reporting Guidelines'. Please confirm you have submitted this list.	Not Applicable	

Data Availability

Data availability	Information included in the manuscript?	In which section is the information available? (Reagents and Tools Table, Materials and Methods, Figures, Data Availability Section)
Have **primary datasets** been deposited according to the journal's guidelines (see 'Data Deposition' section) and the respective accession numbers provided in the Data Availability Section?	Yes	Mass spectrometry data: Results section, Dataset EV1 and Data Availability Section
Were **human clinical and genomic datasets** deposited in a public access-controlled repository in accordance to ethical obligations to the patients and to the applicable consent agreement?	Not Applicable	
Are **computational models** that are central and integral to a study available without restrictions in a machine-readable form? Were the relevant accession numbers or links provided?	Not Applicable	
If publicly available data were reused, provide the respective **data citations in the reference list**.	Not Applicable	
